# Feeding, caregiving practices, and developmental delay among children under five in lowland Nepal: a community-based cross-sectional survey

**DOI:** 10.1186/s12889-022-13776-8

**Published:** 2022-09-10

**Authors:** Sophiya Dulal, Audrey Prost, Surendra Karki, Dafna Merom, Bhim Prasad Shrestha, Bishnu Bhandari, Dharma S. Manandhar, David Osrin, Anthony Costello, Naomi M. Saville

**Affiliations:** 1 Western Sydney University, School of Health Sciences, Locked Bag 1797, Penrith, Sydney, NSW 2571 Australia; 2 UCL Institute for Global Health, 30 Guilford Street, London, WC1N 1EH UK; 3 School of Population Health, University of New South Wales, Sydney, Australia; 4 Research and Development, Australian Red Cross Lifeblood, Sydney, Australia; 5 Mother and Infant Research Activities (MIRA), GPO Box 921, Kathmandu, Nepal

**Keywords:** Infant, Young children, Feeding, Caregiving, Early child development, Nepal

## Abstract

**Background:**

Nurturing care, including adequate nutrition, responsive caregiving and early learning, is critical to early childhood development. In Nepal, national surveys highlight inequity in feeding and caregiving practices for young children. Our objective was to describe infant and young child feeding (IYCF) and cognitive and socio-emotional caregiving practices among caregivers of children under five in Dhanusha district, Nepal, and to explore socio-demographic and economic factors associated with these practices.

**Methods:**

We did a cross-sectional analysis of a subset of data from the MIRA Dhanusha cluster randomised controlled trial, including mother-child dyads (*N* = 1360), sampled when children were median age 46 days and a follow-up survey of the same mother-child dyads (*N* = 1352) when children were median age 38 months. We used World Health Organization IYCF indicators and questions from the Multiple Indicator Cluster Survey-4 tool to obtain information on IYCF and cognitive and socio-emotional caregiving practices. Using multivariable logistic regression models, potential explanatory household, parental and child-level variables were tested to determine their independent associations with IYCF and caregiving indicators.

**Results:**

The prevalence of feeding indicators varied. IYCF indicators, including ever breastfed (99%), exclusive breastfeeding (24-hour recall) (89%), and vegetable/fruit consumption (69%) were common. Problem areas were early initiation of breastfeeding (16%), colostrum feeding (67%), no pre-lacteal feeding (53%), timely introduction of complementary feeding (56%), minimum dietary diversity (49%) and animal-source food consumption (23%). Amongst caregiving indicators, access to 3+ children’s books (7%), early stimulation and responsive caregiving (11%), and participation in early childhood education (27%) were of particular concern, while 64% had access to 2+ toys and 71% received adequate care. According to the Early Child Development Index score, only 38% of children were developmentally on track. Younger children from poor households, whose mothers were young, had not received antenatal visits and delivered at home were at higher risk of poor IYCF and caregiving practices.

**Conclusions:**

Suboptimal caregiving practices, inappropriate early breastfeeding practices, delayed introduction of complementary foods, inadequate dietary diversity and low animal-source food consumption are challenges in lowland Nepal. We call for urgent integrated nutrition and caregiving interventions, especially as interventions for child development are lacking in Nepal.

**Supplementary Information:**

The online version contains supplementary material available at 10.1186/s12889-022-13776-8.

## Key points

What is already known about this topic?

- Inequity in nutritional intake, parental caregiving practices, and early learning opportunities for young children are evident in Nepal.
- Currently, Nepal has no caregiving interventions for children under 3 years of age.
- Systematic reviews have emphasised the benefits of integrated nutrition and caregiving interventions for children’s development in early life.

What does this study add?

- Sub-optimal caregiving practices, inappropriate early breastfeeding practices, delayed introduction of complementary foods, inadequate dietary diversity and low animal-source food consumption are challenges in lowland Nepal.
- Factors associated with poor IYCF and caregiving practices included younger children, those from poor households, whose mothers were young, had received less than three antenatal visits and delivered at home.

What do the new findings imply?

- Integrated nutrition and caregiving interventions for children’s development should prioritise young children in the first 3 years of life, particularly those from disadvantaged groups and improve health services for adolescent and young mothers to optimise feeding and caregiving practices in lowland Nepal.

## Background

Early Childhood Development (ECD) provides a critical foundation for long-term health, well-being, and productivity [1, 2]. Fostering nurturing care is a priority as it enhances the development of young children [3]. Nurturing care is characterised as a stable environment established by parents and other caregivers to promote aspects of ECD. These include children’s good health and adequate nutrition, safety and security, responsive caregiving and early learning through interaction opportunities, emotional support [2, 3].

Adequate nutrition and caregiving during early childhood is essential to ensure the healthy development of young children [4, 5]. Current recommendations for Infant and Young Child Feeding (IYCF) practices include breastfeeding within the first hour of birth, exclusive breastfeeding of infants under 6 months and then providing nutritionally adequate and safe complementary foods at sufficient frequency while breastfeeding up to 2 years of age [6]. Likewise, caregiving is a multidimensional construct. Two critical dimensions are cognitive and socio-emotional caregiving [7]. Cognitive caregiving refers to the strategies parents can use to help children engage with their environment; for example, describing objects, demonstrating activities and offering learning opportunities [7]. Socio-emotional caregiving includes physical and verbal actions that stimulate children’s attention, performance and experiences [7, 8].

Recent systematic reviews emphasise the benefits of integrated nutrition and caregiving (including responsive caregiving and early learning) interventions for children’s development in early life [9–11]. Moreover, a global commitment to promote ECD advocates integrating nutrition and caregiving interventions to establish a holistic early childhood care programme to enhance children’s development [2, 3, 11, 12]. In Nepal, caregiving interventions primarily focus on increasing education access for children aged 3 to 5 years through Early Childhood Education (ECE) [13]. In contrast, Nepal does not have any caregiving interventions focusing on children below the age of three [13], which is a critical phase for child development [3, 4]. An opportunity exists to scale up caregiving interventions by integrating them with current nutrition programmes. Large-scale nutrition programmes led by the governmental and non-governmental organisations in Nepal are in place to address undernutrition in children under 5 years [13–15]. However, to effectively inform and improve the integration of nutrition and caregiving interventions, a deeper understanding of the common underlying factors impeding both IYCF and cognitive and socio-emotional caregiving practices for young children is necessary. Our study aimed to address this need.

In 2019, Nepal’s nationally representative Multiple Indicator Cluster Survey (MICS) showed inequity in the nutritional intake, parental caregiving practices, and early learning opportunities for young children, particularly among disadvantaged groups and in resource-constrained settings [16]. Children in Province 2 (Madhesh Pradesh) have the greatest disadvantages in terms of IYCF and caregiving practices [16, 17] but the socio-demographic and economic factors driving these practices or how factors affecting poor IYCF and caregiving practices interact remain unknown. To address this gap, we (1) documented practices related to IYCF and cognitive and socio-emotional caregiving among caregivers of children under age five in Dhanusha district, Nepal, and (2) examined socio-demographic and economic factors at household, parental, and child-level associated with recommended IYCF and caregiving practices.

## Methods

### Study setting

We conducted our study in Dhanusha district in Province 2 in the *Terai* (lowland) region of Nepal. The district covers 1180 km^2^ and has a population of 754,777 [17]. According to the 2011 census, most people live in rural areas (89%) and over half of households are involved in agriculture [18]. The average household size is 5.5, with 35% having access to toilet facilities; only 13% having access to clean drinking water and 40% of women are illiterate [18]. The common language is Maithili and the main religion is Hinduism (89%), followed by Islam (9%).

### Study design, sample size and participants

#### The MIRA Dhanusha cluster randomised controlled trial (cRCT)

Data for the cross-sectional analyses came from the MIRA Dhanusha Trial, a community-based factorial cRCT, that was implemented between 2006 and 2011 in 60 Village Development Committees (VDCs) (now restructured into eleven urban- and six rural-municipalities) in Dhanusha district by a Nepalese non-government organisation, Mother and Infant Research Activities (MIRA), and University College London. The main aim of the trial was to test the effect of community mobilisation through women’s groups following a Participatory Learning and Action (PLA) cycle and community-based neonatal sepsis management on neonatal mortality, home care and health care seeking, maternal and infant nutrition-related behaviour and anthropometric status [19]. Between September 2006 and June 2011, data on mother-child dyads who participated in the trial were collected by a team of 39 trained interviewers at 6 weeks postpartum (6-week questionnaire).

#### Cross-sectional follow-up survey of children born to mothers participating in the cRCT

A random sample of 1365 out of 35,208 mother-child dyads was drawn from the trial dataset and followed by seven female interviewers between 16 September and 15 December 2011, when the children were between 7 and 62 months old. We generated a list of participants from the main trial with a minimum sample of 20 eligible respondents in each of 60 clusters. Interviewers then exhaustively sampled 20 to 31 respondents per cluster from this list, depending on the number found at home when the follow-up visits were made. This resulted in a total sample of 1365 children over 60 study clusters.

For both the 6-week questionnaire and follow-up surveys, informed verbal consent was obtained from all participants. The structured pre-coded questionnaire was pre-tested with mothers to ensure that the questions and response-formats were clear to minimise information bias. All interviews were conducted in participants’ homes where caregivers felt comfortable and where interviewers could observe the environment and probe to validate answers. Interviewers were experienced and had been trained on protocols to minimise recall and social desirability bias. Almost all interviews were conducted in Maithili (98.7%).

For the study, we used a subset of data obtained from the 6-week questionnaire (*N* = 1360) between 21 February 2007 and 10 July 2008 and a follow-up survey of the same mother-child dyads (*N* = 1352). The median age of children was 46 days (range 0–12 months) at the 6-week questionnaire and 38 months (range 7–59 months) at follow-up. We excluded five children from the 6-week postpartum interview aged over 12 months because they did not meet the age range criteria for inclusion (*n* = 2) or had missing age data (*n* = 3). For similar reasons, we removed 13 children aged over 59 months from the follow-up survey (did not meet the inclusion criteria (*n* = 12) and missing age data (*n* = 1)). Supplementary Fig. 1 shows the participant flow diagram.

### Study measures

#### Selection of outcome variables

We used the World Health Organization (WHO) indicators to obtain information on IYCF practices [20] and questions from the United Nations Children Fund’s (UNICEF) MICS-4 survey tools to obtain information on cognitive and socio-emotional caregiving practices [21]. Supplementary Table 1 summarises the indicators available. At the postpartum 6-week questionnaire, we gathered information about breastfeeding immediately after birth (time of breastfeeding initiation, colostrum feeding and no pre-lacteal feeding) and other feeding behaviours in the past 24-hours when children were median age 46 days. We obtained data on complementary feeding practices (recall for all children sampled) and cognitive and socio-emotional caregiving practices from the follow-up survey when children were median age 38 months.

#### Outcome variables

##### IYCF indicators

All IYCF indicators were constructed according to WHO 2021 recommendations [6], except for Minimum Dietary Diversity (MDD). The eight food groups in the recent WHO recommendation include (i) breast milk, (ii) grains, roots, tubers, and plantains, (iii) pulses, nuts and seeds, (iv) dairy products (milk, yoghurt cheese), (v) flesh foods, (meat, fish, poultry, and liver/organ meats), (vi) eggs, (vii) vitamin-A rich fruits and vegetables and (viii) other fruits and vegetables. The new group “breastfed in recall period” was not available in our dataset. We used the following definitions of IYCF indicators: (1) Ever breastfed: proportion of children (median age 46 days) who were ever breastfed at the time of the 6-week questionnaire; (2) Early initiation of breastfeeding: proportion of newborns who were put to the breast within 1 h of birth; (3) exclusive breastfeeding for the past 24-hours: proportion of children aged 0–5 months who were exclusively breastfed for the past 24-hours in the 6-week questionnaire; (4) timing of introduction of complementary foods: proportion of children aged 7–59 months who received solid, semi-solid or soft foods within 6–8 months of age; (5) MDD: proportion of children 7–59 months of age who received foods from at least four out of seven food groups in the past 24-hours; (6) consumption of animal-source foods: proportion of children aged 7–59 months who had consumed eggs or flesh foods (meat and fish) in the past 24-hours, and (7) consumption of fruits and vegetables: proportion of children aged 7–59 months who had consumed any fruits or vegetables in the past 24-hours.

We used the WHO indicators for children aged 6–23 months to describe the diets of children aged 7–59 months, which has been done previously [22, 23]. We also included, from the 6-week questionnaire, colostrum feeding (feeding child with the first yellowish human breast milk) and no pre-lacteal feeding (not providing any food except mother’s milk to a newborn before initiating breastfeeding) because these are significant barriers to exclusive breastfeeding in Nepal [24, 25]. Supplementary Table 2 summarises the timing of data collection for each indicator and the age ranges over which we present results.

##### Cognitive and socio-emotional caregiving indicators

Cognitive and socio-emotional caregiving indicators included (1) access to learning materials, defined as the proportion of children under age five who had three or more children’s books and two or more types of toys at home; (2) early stimulation and responsive care, defined as the proportion of children aged 24–59 months with whom mother/father/other adults had engaged in four or more activities such as reading, storytelling, singing, taking the child outside, playing or counting and drawing at home in the past 3 days; (3) adequate supervision, defined as the proportion of children under age five who were not left alone or in the company of another child younger than 10 years for more than an hour at least once in the last week; (4) participation in ECE, defined as the proportion of children aged 36–59 months who attended some form of ECE programme outside the home; and (5) Early Child Development Index (ECDI) score, defined as the proportion of children aged 36–59 months who were developmentally on track in three of the four domains described in Table 1.

**Table 1:** Definitions of developmental domains for children aged 36–59 months

Developmental domains	Definition
**Literacy and numeracy**	A child can read at least four simple, well-known words and know the name and recognise the symbols of all numbers from 1 to 5.
**Physical**	A child can pick up a small object like a stick or stone, with two fingers from the ground, and the mother/caregiver does not indicate that the child is sometimes too sick to play.
**Socio-emotional**	A child gets along well with other children, does not kick, bite or hit other children, and does not get distracted easily.
**Learning**	Ability to follow a simple direction to do something correctly, and when instructed to do something, a child can perform it independently.

#### Explanatory variables

Data for explanatory variables were collected in the 6-week questionnaire and included information on the socio-demographic and economic characteristics of children and their parents. We selected factors that had been found to be associated with IYCF or caregiving in the literature [24–30] and which were available in our dataset. We categorised explanatory variables into household, parental, and child-level. Household-level variables included wealth quintiles, months of adequate household food provisioning (MAHFP), migration of any household member, household size (adult family members), access to health care, ethnicity, and religion. We derived a household wealth index using principal component analysis from household characteristics and ownership of household assets and then divided the wealth score into five equal quintiles [31]. Parental level variables included mother’s education, father’s education, mother’s age (in years), parity, antenatal visits and place of delivery. Individual child-level variables included age (in months) and sex. Supplementary Table 3 provided detailed information on definition and categorisation of the potential explanatory variables.

### Statistical analyses

**Fig. 1:**
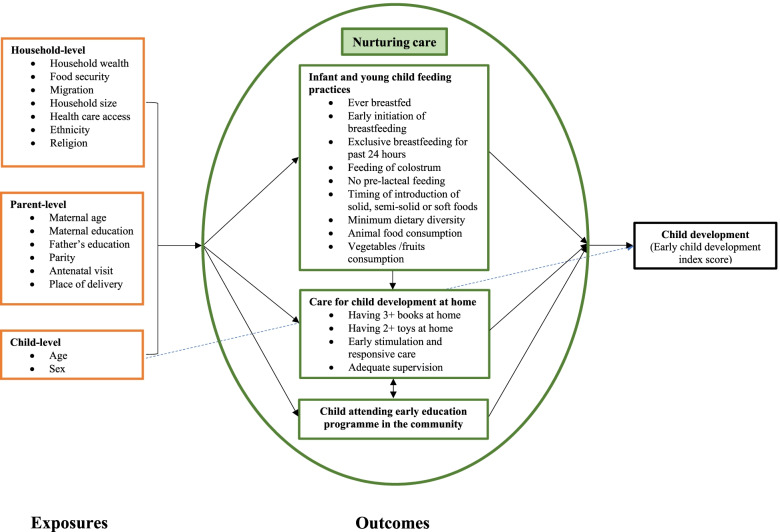
Conceptual framework for determinants of feeding and caregiving practices. Single-headed solid black and dotted blue arrows represent directional paths, and double-headed arrows indicate the variables that are assumed to be correlated

We adapted the *Nurturing Care Framework* published in the Lancet series “Advancing ECD: from science to scale” [3] and limited to factors available in our data, to develop an analytical framework to structure the variables selected for the analysis (Fig. 1). Our study looks at three components of nurturing care: nutrition, responsive caregiving and early learning. The conceptual framework consisted of household-, parental- and child-level explanatory variables, which were assumed to influence IYCF and cognitive and socio-emotional caregiving practices directly or indirectly. The age and sex of the child were also considered to independently affect the developmental outcome (ECDI score). Groups of explanatory variables were entered in hierarchical order into a multivariable modelling procedure shown in Supplementary Fig. 2 and described below. We conducted the statistical analysis using STATA 16.1.

We used descriptive statistics to assess household, parental and child characteristics and the prevalence of feeding and caregiving practices across the sample. First, we assessed the association between variables within the three categories with the outcome variables using univariable analyses. Then, we examined the role of factors using multivariable mixed-effects logistic regression. As a standard approach, we used *p* < 0.2 as a threshold to select candidate variables from the univariable analyses to enter the multivariable regression models [25, 32]. All multivariable models were adjusted for study design by adding cluster (VDC) as a random effect, trial arm as a fixed effect and for non-modifiable explanatory variables child age and sex. Similarly, we pre-identified essential predictors of feeding practices (mother’s age and education) [25, 26] and caregiving (mother’s education and household wealth index) [27–29] and included them in all multivariable models.

We ran three different multivariable logistic regression models separately for each outcome variable (except the ever breastfed indicator since almost all children were breastfed). Model 1 included household-level variables with *p* < 0.2 in univariable analyses. We retained household variables with *p* < 0.05 in Model 1 in the second set of multivariable models (Model 2). Model 2 included household variables kept from Model 1 and parental-level variables with *p* < 0.2 in univariable analyses. The final fully adjusted model (Model 3) included household and parental-level variables with *p* < 0.05 in Model 2. We report the Adjusted Odds Ratio (AOR) for variables retained in Model 3 with 95% Confidence Interval (CI). A *p-*value < 0.05 was considered statistically significant in all models. Finally, we used a Venn diagram to present the cluster of factors associated with both optimal feeding and cognitive and socio-emotional caregiving practices.

## Results

### Participant characteristics

All participants completed the follow-up survey. Participant characteristics are summarised in Table 2. In the year preceding the 6-week questionnaire, 64% of mothers reported adequate household food provisioning for up to 12 months. Over half (51%) had a family member working away, and most lived in households with more than six members (77%). Only 14% of mothers and children had access to health care. Almost a third (31%) were from the most disadvantaged groups (Dalits/Muslims), and nearly all were Hindu (88%). Mothers’ mean age was 26.5 years. Only 19% of mothers had attended school and 82% of them could not read or read with some difficulty. Nearly two-thirds of fathers (64%) had not attended school. Around a third of mothers were primiparous (32%); over a third had not received antenatal visits, and home delivery was common (78%). There were more boys (54%) than girls (47%) in our sample.

**Table 2:** Participant Characteristics

Variables	n (%)
**Household characteristics**^a^	
Wealth Quintile (*N* = 1299)	
Lowest	243 (18.7)
Second	260 (20.0)
Middle	292 (22.5)
Fourth	243 (18.7)
Highest	261 (20.1)
Months of adequate food provisioning (MAHFP) (*N* = 1347)	
For up to 7 months	169 (12.5)
For 8 to 11 months	312 (23.2)
For 12 months	866 (64.3)
Migration of at least one household member (*N* = 1343)	
No	654 (48.7)
Yes	689 (51.3)
Household size (*N* = 1340)	
1–5 members	311 (23.2)
6–10 members	806 (60.1)
≥ 11 members	223 (16.6)
Health care access (*N* = 1250)	
No	1077 (86.2)
Yes	173 (13.8)
Ethnicity/Caste (*N* = 1352)	
Dalit/Muslim (most disadvantaged group)	418 (30.9)
Janajati/other terai caste (Sudi/Teli)	639 (47.3)
Yadav/Brahmin (least disadvantaged group)	295 (21.8)
Religion (*N* = 1352)	
Hindu	1194 (88.3)
Non-Hindu (mostly Muslim)	158 (11.7)
**Parental characteristics**^a^	
Maternal age, mean (SD) (*N* = 1351)	26.5 (5.0)
15–24 years	521 (38.6)
25–34 years	729 (54.0)
35–45 years	101 (7.5)
No of previous pregnancies (*N* = 1351)	
One	430 (31.8)
Two	366 (27.1)
Three	256 (18.9)
Four or more	299 (22.1)
Maternal education (*N* = 1352)	
Never went to school	1097 (81.1)
Primary	98 (7.2)
Secondary and above	157 (11.6)
Mother’s literacy (*N* = 1345)	
Cannot read or with some difficulty	1107 (82.3)
Can read easily	238 (17.7)
Father’s education (*N* = 1352)	
Never went to school	867 (64.1)
Primary	161 (11.9)
Secondary and above	324 (24.0)
Antenatal visits (*N* = 1352)	
None	435 (32.2)
1–3 visits	642 (47.5)
4 +	275 (20.3)
Place of delivery (*N* = 1326)	
Home	1038 (78.3)
Health facility	288 (21.7)
**Child characteristics**	
Child sex (*N* = 1352)	
Male	723 (53.5)
Female	629 (46.5)
Child age group in days at 6-week interview ^a^, median (IQR)^b^ (*N* = 1360)	46 (34)
< 46 days	678 (49.9)
46 to 91 days	503 (37.0)
92 to 182 days (3–5 months)	135 (9.9)
183 to 366 days (6–12 months)	44 (3.2)
Children age in months at follow-up^c ^median (IQR) (*N* = 1352)	38 (29.7)
7–11	124 (9.2)
12–23	288 (21.3)
24–35	219 (16.2)
36–47	300 (22.2)
48–59	421 (31.1)
**Trial allocation (*N*** **= 1352)**	
Control	459 (34.0)
Women’s group only	372 (28.0)
Women’s group and sepsis	320 (23.7)
Sepsis only	201 (14.9)

^a^Collected in the post-neonatal period when the index child was median 46 days old

^b^IQR Interquartile Range

^c^Collected in the follow-up cross-sectional survey questionnaire when the child was median age 38 month

### Infant and young child feeding practices

Table 3 describes caregivers’ recall of breastfeeding practices for children sampled at median age 46 days and complementary feeding practices for children sampled at median age 38 months. Almost all children aged 0–12 months were ever breastfed (99%); however, only 16% were breastfed within 1 h of birth; 67% of children were fed colostrum and 53% were not given pre-lacteal feeding. Most children aged 0–5 months (89%) were exclusively breastfed for the 24-hours preceding the survey. Slightly more than half of children aged 7–59 months (56%) received complementary foods at 6–8 months. The median number of food groups consumed by children aged 7–23 months in the last 24-hours (at median age 14 months) was 3.4 and only 49% of children aged 7–23 months had achieved 24-hour MDD (≥4 items). Consumption of vegetables and fruits at 7–23 months was higher (69%) than animal-source foods (23%). Consumption of fruits and vegetables increased from 44% at 7–11 months to 80% at 12–23 months. Consumption of animal-source foods increased from 13 to 27% over the same age periods. After 24 months, a small gradual increase in consumption with age was found (Supplementary Fig. 3).

**Table 3:** Infant and young child feeding practices for children under 59 months in Dhanusha district

Indicators collected when children were median age 46 days n (%)		n (%)
Ever breastfed (*N* = 1358)		
No	8 (0.6)	
Yes	1350 (99.3)	
Breastfeeding initiation (recall of the first days of life) (*N* = 1350)		
Within 1 hour of birth	215 (15.9)	
1 to 24-hours	348 (25.8)	
After 24-hours	787 (58.3)	
Colostrum feeding (recall of the first days of life) (*N* = 1343)		
No	449 (33.4)	
Yes	894 (66.6)	
No pre-lacteal feeding (recall of the first days of life) (*N* = 1342)		
No	627 (46.7)	
Yes	715 (53.3)	
Exclusive breastfeeding for past 24-hours amongst children 0–5 months at interview (at median age 45 days) (*N* = 1314)		
No	141 (10.7)	
Yes	1173 (89.3)	
**Indicators collected when children were median age 38 months (7–59 months) at follow-up**		
Timing of introduction of solid-semi-solid or soft foods (recall for all children sampled), median (IQR)^a^ **(***N* = 1313)	8 (4)	
Before six months	53 (4.0)	
6–8 months	731 (55.7)	
At nine months and after	529 (40.3)	
	**All children 7 to 59 months (median 38 m) (*N*** **= 1352)**	**Children 7 to 23 months only (median 14 m) (*N*** **= 412)**
Minimum dietary diversity achieved in past 24-hours		
mean (SD)	3.8 (1.2)	3.4 (1.4)
No	506 (37.4)	211 (51.2)
Yes	846 (62.6)	201 (48.8)
Consumption of animal-source foods (eggs and flesh food) in the past 24-hours (*N* = 1352)		
No	961 (71.1)	317 (77)
Yes	391 (28.9)	95 (23.1)
Consumption of fruits and vegetables in past 24-hours (*N* = 1352)		
No	214 (15.8)	127 (30.8)
Yes	1138 (84.2)	285 (69.2)

^a^IQR Interquartile Range

### Cognitive and socio-emotional caregiving practices

Table 4 and Supplementary Fig. 4 describe cognitive and socio-emotional caregiving practices for children aged 7–59 months. Most children (91%) used household objects as toys and 69% had toys from shops. Fewer children (12%) had homemade toys, and very few (7%) had three or more children’s books. The absence of books and mothers’ low literacy rates meant that few children aged 7–59 months were read to (14%). Reading (2%), storytelling (8%) and counting and drawing (5%) by caregivers were uncommon in the 7–23 months age group, but more common in the 24–59 months age group (19, 31, and 27%, respectively). More than two-thirds (69%) of children were never played with and most (72%) were never sung to by caregivers. Taking children outside (65%) was the most common activity and was primarily done by mothers; other activities such as reading (7%), playing (15%), counting, and drawing (13%) for children were done chiefly by their older siblings and storytelling and singing were done mainly by other adults (including grandparents) (13%). Only 11% of children aged 7–59 months received any kind of early stimulation or responsive care from any caregiver in the 3 days preceding the survey, and three-quarters of children (71%) received adequate supervision.

**Table 4:** Cognitive and socio-emotional caregiving practices for children under 59 months in Dhanusha district

Indicators	7–23 months	24–59 months	7–59 months
	n (%)	n (%)	n (%)
Access to children’s books	**412**	**940**	**1352**
Has less than three children’s books	376 (91.3)	833 (93.9)	1259 (93.1)
Has three or more children’s books	36 (8.7)	57 (6.1)	93 (6.9)
Types of toys	**412**	**940**	**1352**
Homemade toys	17 (4.5)	134 (14.6)	151 (11.6)
Toys from shop	277 (73.5)	623 (67.7)	900 (69.4)
Household objects used as toys	322 (85.4)	852 (92.6)	1174 (90.5)
Access to toys	**412**	**940**	**1352**
Has less than two toys	181 (43.9)	306 (32.6)	487 (36.0)
Has two or more toys	231 (56.1)	634 (67.5)	865 (64.0)
Support for learning			
Child read to	**378**	**921**	**1299**
No	370 (98)	750 (81.4)	1120 (86.2)
Yes	8 (2.1)	171 (18.6)	179 (13.8)
Child told stories	**378**	**919**	**1297**
No	349 (92.3)	634 (69)	983 (75.8)
Yes	29 (7.7)	285 (31)	314 (24.2)
Child sung to	**378**	**921**	**1299**
No	321 (84.9)	619 (67.2)	940 (72.4)
Yes	57 (15.1)	302 (32.8)	359 (27.6)
Child taken outside	**377**	**920**	**1297**
No	163 (43.2)	291 (31.6)	454 (35)
Yes	214 (56.8)	629 (68.4)	843 (65)
Child engaged in playing	**378**	**920**	**1298**
No	302 (79.9)	587 (63.8)	889 (68.5)
Yes	76 (20.1)	333 (36.2)	409 (31.5)
Child engaged in counting or drawing	**378**	**921**	**1299**
No	361 (95.5)	672 (73)	1033 (79.5)
Yes	17 (4.5)	249 (27)	266 (20.5)
Early stimulation and responsive caregiving ^a^	**412**	**940**	**1352**
No	401 (97.3)	804 (85.3)	1205 (89.1)
Yes	11 (2.7)	136 (14.5)	147 (10.9)
Adequate supervision ^**b**^	**378**	**921**	**1299**
No	83 (22)	300 (32.6)	383 (29.5)
Yes	295 (78)	621 (67.4)	916 (70.5)

^a^Mother/Father/Adult (adult include maternal and paternal grandparents) has engaged with children in four or more support for learning activities in the last three days

^b^Children were not left alone or in the company of another child younger than 10 years for more than one hour at least once in the previous week

About three quarters (74%) of children aged 36–59 months did not participate in ECE, and over half (62%) were not on developmental track according to the ECDI score (Table 5). Supplementary Fig. 5 shows differences in ECDI scores by wealth quintile disaggregated by sex. A higher proportion of girls were on track than boys in the lowest (39% vs 30%), second (39% vs 29%), and fourth (48% vs 30%) wealth quintiles.

**Table 5:** Early childhood development index score for children aged 36–59 months in Dhanusha district

Indicators	n (%)
Participate in early childhood education (attending playgroup/ community childcare) (*N* = 717)	
No	527 (73.5)
Yes	190 (26.5)
Early childhood development index (ECDI)	
Children on track in (*N* = 721)	
Literacy-numeracy domain (can count to 5 and say four simple words)	
No	346 (48)
Yes	375 (52)
Physical domain (pick up a small object with two fingers and is not too sick to play)	
No	363 (50.4)
Yes	358 (49.7)
Socio-emotional domain (gets along with others/ does not kick or bite/ not distracted)	
No	520 (72.1)
Yes	201 (27.9)
Learning domain (follows simple directions and can do something independently)	
No	42 (5.8)
Yes	679 (94.2)
On track in at least 3 of 4 domains	
No	444 (61.6)
Yes	277 (38.4)

### Factors associated with infant and young child feeding indicators

**Fig. 2:**
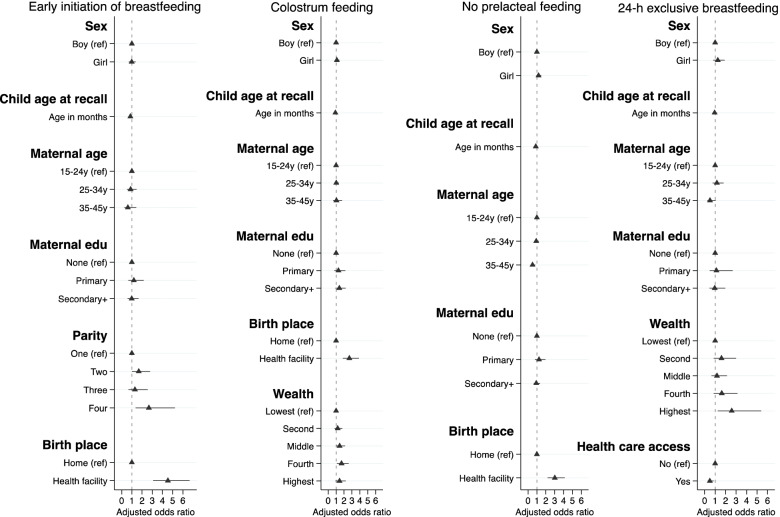
Final adjusted model showing factors affecting breastfeeding practices among children aged 0–12 months. The median age of children for three breastfeeding indicators - early time of initiation of breastfeeding, colostrum feeding and no pre-lacteal feeding were 46 days (range 0–12 months) and for exclusive breastfeeding (24-hours recall) was 45 days (range 0–5 months). Each model was adjusted for the effect of clustering using cluster sites as a random effect and study trial arms as a fixed effect. We adjusted all breastfeeding indicators by child’s age in months at recall as a continuous variable in the multivariable regression models

**Fig. 3:**
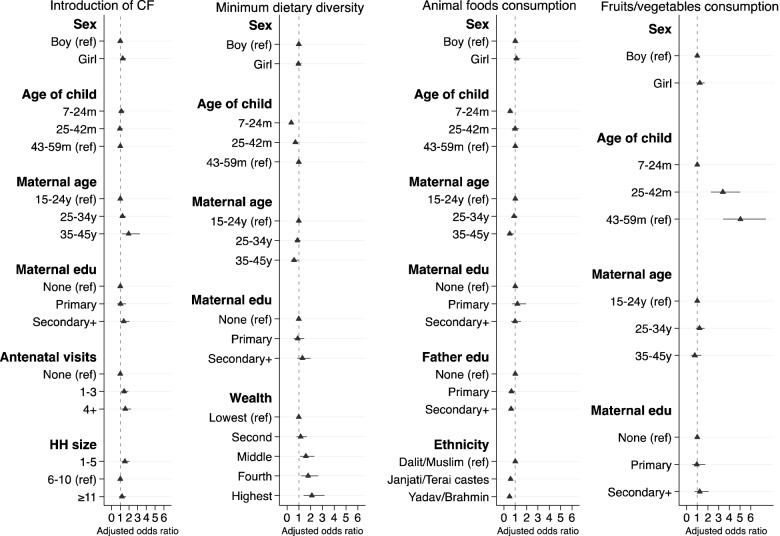
Final adjusted model showing factors affecting complementary feeding practices among children of median age 38 months (range 7–59 months). Each model was adjusted for the effect of clustering using cluster sites as a random effect and study trial arms as a fixed effect. CF: Complementary Feeding

Figures 2 and 3 present the variables’ independent associations with each IYCF indicator. Supplementary Tables 4–11 provide numerical details of regression output.

The odds of breastfeeding initiation within 1 h of birth were greater in children whose mothers had four or more previous births (AOR 2.66; 95% CI 1.35, 5.23, ref.: one) and who were born in a health facility (AOR 4.53; 95% CI 3.09, 6.65, ref.: home). The odds of feeding colostrum were greater in children from the fourth quintile (AOR 1.69; 95% CI 1.10, 2.60, ref.: lowest quintile) and those born in a health facility (AOR 2.68; 95% CI 1.85, 3.88, ref.: home). The odds of not giving pre-lacteal feeding were greater in those born in a health facility (AOR 3.02; 95% CI 2.20, 4.14, ref.: home) and lower in children of older mothers (35–45 years, AOR 0.50; 95% CI 0.31, 0.81, ref.: 15- 24 years). The odds of breastfeeding initiation within 1 h of birth, feeding colostrum and not giving pre-lacteal feeding in the first days of life were lower as child’s age in months at the time of recall increased (see supplementary Table 4, 5 and 6). The odds of exclusive breastfeeding recalled over the past 24-hours before the survey when children were 0–5 months were greater in the highest wealth quintile (AOR 2.58; 95% CI 1.24, 5.39, ref.: lowest quintile) and, unexpectedly, lower for children who had access to health care (AOR 0.50; 95% CI 0.29, 0.85, ref.: no access).

The odds of children receiving complementary foods at 6–8 months age were greater in girls (AOR 1.29; 95% CI 1.01, 1.64, ref.: boys), in children of older mothers (35–45 years, AOR 1.95; 95% CI 1.18, 3.22, ref.: 15-24 years), whose mothers had antenatal visits (1–3 visits, AOR 1.45; 95% CI 1.09, 1.91; 4 or more visits, AOR 1.58; 95% CI 1.11, 2.24, ref.: none) or came from smaller households (1–5 members, AOR 1.54; 95% CI 1.15, 2.06, ref.: 6-10 members).

The odds of achieving MDD among children aged 7–59 months increased with wealth to a maximum of 2.12 in the highest quintile. Younger children (7–24 months, AOR 0.36; 95% CI 0.27, 0.48; 25–42 months, AOR 0.70; 95% CI 0.52, 0.95, ref.: 43-59 months) and those whose mothers were aged 35–45 years (AOR 0.60; 95% CI 0.38, 0.97, ref.: 15-24 years) were less likely to achieve MDD. The odds of consumption of animal-source foods were lower in younger children (7–24 months, AOR 0.55; 95% CI 0.40, 0.75, ref.: 43 - 59 months), and amongst those whose mothers were aged 35–45 years (AOR 0.52; 95% CI 0.30, 0.90, ref.: 15-24 years), whose fathers had higher education (primary, AOR 0.66; 95% CI 0.43, 1.00; secondary and above, AOR 0.63; 95% CI 0.45, 0.87; ref.: none) and were from relatively advantaged caste groups (Janjati/other terai, AOR 0.58; 95% CI 0.43, 0.78; Yadav/Brahmin, AOR 0.49; 95% CI 0.33, 0.71, ref.: Dalit/Muslim). The odds of consuming fruits and vegetables were much lower in younger children (AOR 0.20; 95% CI 0.13, 0.29, ref.: 43 - 59 months) than in the oldest age group.

### Factors associated with cognitive and socio-emotional caregiving practices

**Fig. 4:**
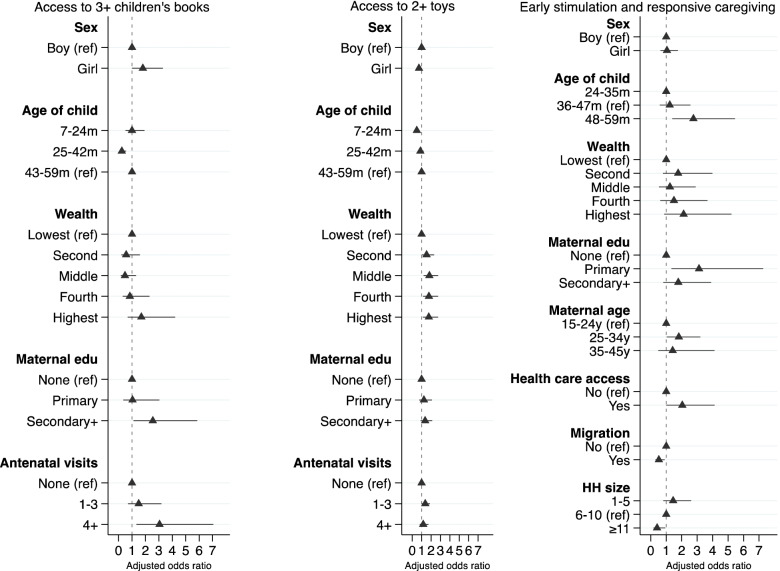
Final adjusted model showing factors affecting caregiving practices among children of median age 38 months (range 7–59 months). Each model was adjusted for the effect of clustering using cluster sites as a random effect and study trial arms as a fixed effect. The age denominator for access to 3+ children’s books and 2+ toys is 7–59 months; support for learning is 24–59 months

**Fig. 5:**
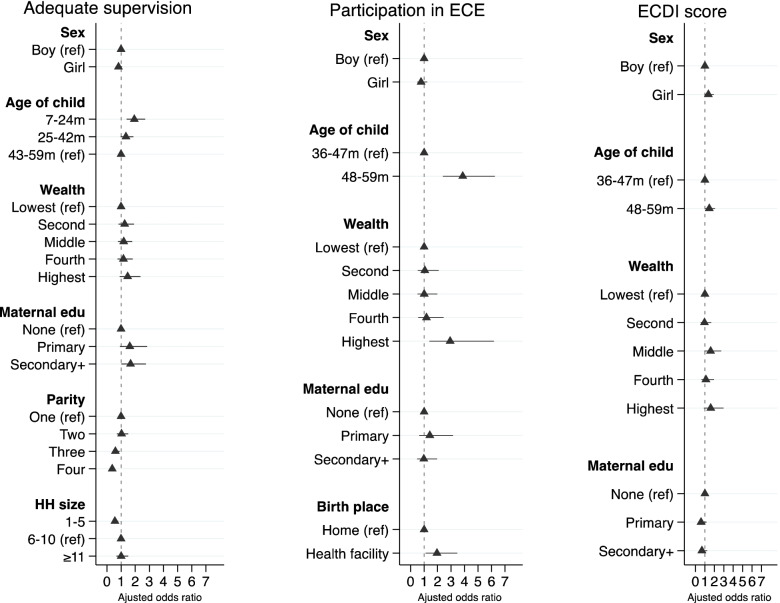
Final adjusted model showing factors affecting caregiving practices among children aged 7–59 months. Each model was adjusted for the effect of clustering using cluster sites as a random effect and study trial arms as a fixed effect. ECE: Early Childhood Education; ECDI: Early Childhood Development Index. The age denominator for adequate supervision is 7–59 months; participation in ECE and ECDI score is 36–59 months

Figures 4 and 5 present the variables’ independent associations with each cognitive and socio-emotional caregiving practice indicator. Supplementary Tables 12–17 provide numerical details of regression output.

The odds of having access to three or more children’s books were 2.6 times greater among children whose mothers had higher education and three times greater in those whose mothers had four or more antenatal visits. The odds of having access to three or more children’s books were lower for younger children (AOR 0.24; 95% CI 0.10, 0.55, ref.: 43-59 months). The odds of access to two or more toys were two times greater for children in all wealth quintiles relative to the poorest and whose mothers had 1–3 antenatal visits (AOR 1.38; 95% CI 1.02, 1.88, ref.: none). Girls (AOR 0.72; 95% CI 0.55, 0.94, ref.: boys) and younger children (AOR 0.49; 95% CI 0.36, 0.66; ref.: 43-59 months) were less likely to have access to two or more toys.

Children whose mothers were aged 25–34 years (AOR 1.82; 95% CI 1.03, 3.21, ref.:15-24 years), had received at least primary education (AOR 3.12; 95% CI 1.34, 7.27, ref.: none), had access to health care (AOR 2.04; 95% CI 1.01, 4.13, ref.: no access) and were aged between 48 and 59 months (AOR 2.76; 95% CI 1.40, 5.45, ref.:24-35 months) were more likely to receive early stimulation and responsive caregiving. Children from large households with 11 or more members (AOR 0.41; 95% CI 0.18, 0.94, ref.: 6-10 members) and where at least one member had migrated (AOR 0.52; 95% CI 0.31, 0.90, ref.: none) had lower odds of receiving early stimulation and responsive caregiving.

Younger children aged 7–24 months (AOR 1.94; 95% CI 1.39, 2.70; ref.: 43-59 months) and those whose mothers had higher education (AOR 1.67; 95% CI 1.02, 2.75, ref.: none) had higher odds of receiving adequate supervision. Children whose mothers had three (AOR 0.60; 95% CI 0.40, 0.89) or more (AOR 0.38; 95% CI 0.26, 0.55, ref.: one) previous births and were from households with 1–5 members (AOR 0.57; 95% CI 0.40, 0.79, ref.: 6-10 members) had lower odds of receiving adequate supervision.

Children from wealthier households (highest quintile AOR 2.93; 95% CI 1.39, 6.19, ref.: lowest quintile), born in a health facility (AOR 1.95; 95% CI 1.10, 3.46, ref.: home) and aged 48–59 months (AOR 3.87; 95% CI 2.40, 6.24, ref.: 36-47 months) were more likely to participate in ECE. Similarly, children aged 48–59 months had greater odds of being developmentally on track according to the ECDI score (AOR 1.46; 95% CI 1.02, 2.08, ref.: 36-47 months).

### Factors affecting both optimal feeding and caregiving practices

**Fig. 6:**
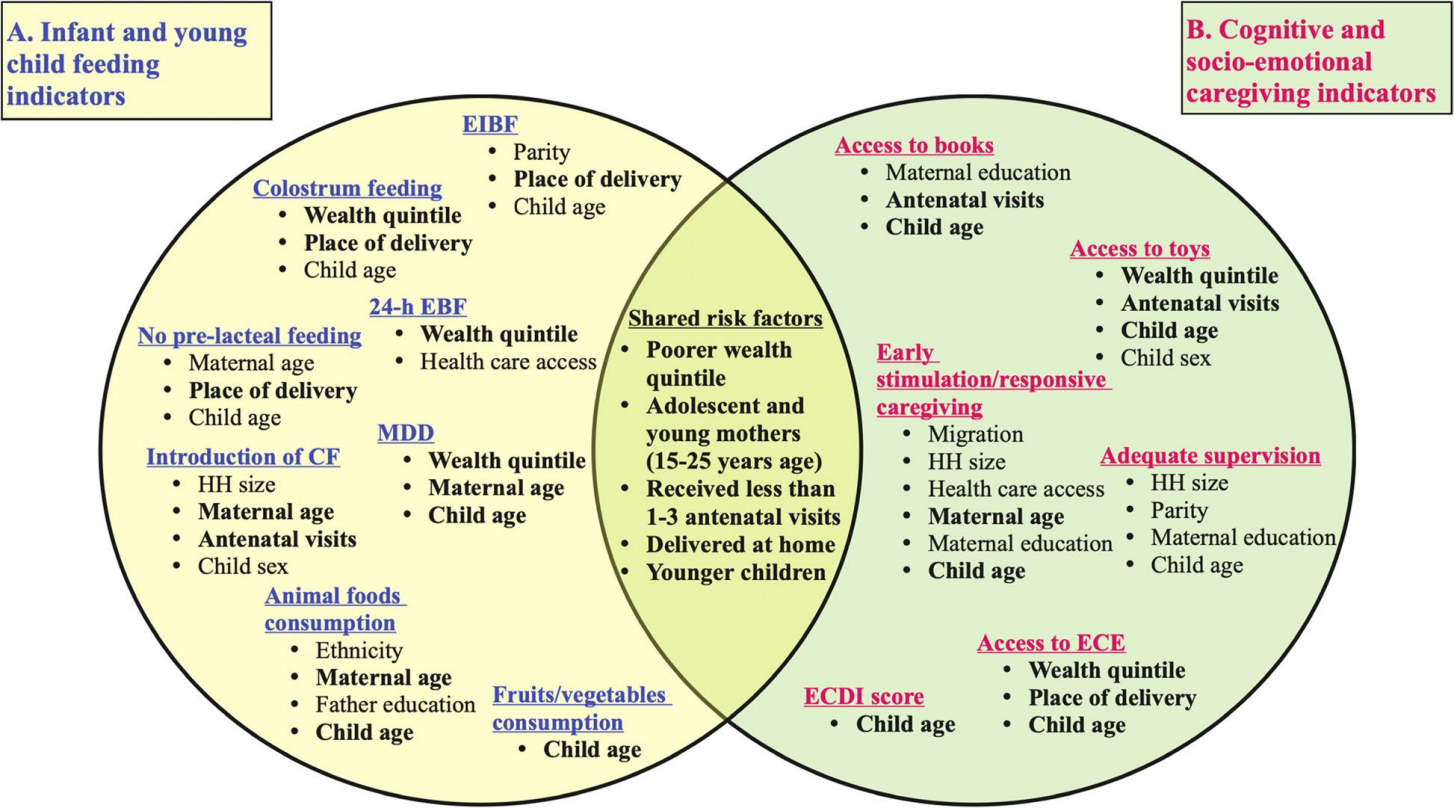
Venn diagram showing shared risk factors between feeding and caregiving indicators. The area where the circles overlap denote factors commonly identified as significant factors for each infant and young child feeding and cognitive and socio-emotional caregiving indicator showing the same direction of association for each outcome in multivariable regression analysis Model 3. EIBF Early Initiation of Breastfeeding, EBF Exclusive Breastfeeding, CF Complementary Feeding, HH Household, MDD Minimum Dietary Diversity, ECE Early Childhood Education, ECDI Early Childhood Development Index

Figure 6 maps the common risk factors associated with IYCF and caregiving outcomes in multivariable regression analysis Model 3. The common risk factors affected both indicators in the same direction, indicating an overlapping risk group (Supplementary Table 18). Children with a higher risk of poor feeding and caregiving practices were younger, from poor households, and were born to adolescent and young mothers (15–24 years) who received few or no antenatal visits and delivered at home.

## Discussion

Our study profiled the prevalence of IYCF and cognitive and socio-emotional caregiving practices among children below 5 years of age in a highly disadvantaged area of the Nepal plains and examined a range of socio-demographic and economic factors associated with IYCF and caregiving practices. Our findings highlight relatively good adherence to IYCF practices for two indicators: exclusive breastfeeding for the past 24-hours at the 6-week questionnaire and consumption of fruits and vegetables. However, IYCF indicators such as early initiation of breastfeeding, colostrum feeding, no pre-lacteal feeds, timely introduction of complementary foods, sufficient dietary diversity and consumption of animal-sourced food were suboptimal in Dhanusha district. Moreover, the prevalence of appropriate cognitive and socio-emotional caregiving practices was low for all indicators, including access to children’s books and toys, early stimulation and responsive care, adequate supervision, participation in ECE and ECDI scores. Overall, only 38% of children in this study were developmentally on track in at least three of the four domains as assessed by the ECDI scores. Younger children from poor households whose mothers were younger, had not made antenatal visits and were delivered at home were the most at risk of poor feeding and caregiving practices.

We compared our findings to regional and national estimates in the Nepal Demographic Health Survey (NDHS) in 2011 and 2016. The prevalence of mothers initiating breastfeeding within 1 h of birth was extremely low (16% vs 31% & 45%), and of children who were not given pre-lacteal feeding was slightly higher in our sample (53% vs 47% & 52%) relative to regional and provincial estimates in NDHS 2011 and 2016, respectively [17, 18]. Colostrum feeding was marginally lower (67% vs 84%) than the national estimates reported by Bhandari et al. [25]. These findings corroborate prior research indicating that optimal breastfeeding practices were less prevalent in the Terai [25, 30]. Similarly, we found that the prevalence of children receiving complementary foods at 6–8 months was lower (56% vs 66% & 84%) than NDHS 2011 and 2016 national estimates [17, 18], whilst children achieving MDD was higher (49% vs 12% & 30%) than the regional average in NDHS 2011 and 2016, respectively [17, 18]. Although the PLA group intervention tested in the trial could have increased child dietary diversity, we adjusted the analysis for any potential effect of the trial intervention. The low prevalence of recommended feeding behaviours relative to national and regional averages highlights the limited progress in improving IYCF practices in the Terai, indicating inadequate coverage or ineffectiveness of IYCF programmes, as highlighted by Cunningham et al. [33].

Concerning caregiving practices, the regional estimate for access to three or more children’s books was lower in MICS 2014 and 2019 (2% & 1%) than in Dhanusha district (7%) [16, 34]. However, Dhanusha district was behind for all other indicators, with fewer children receiving early stimulation and responsive caregiving (11% vs 64% & 68%), adequate supervision (71% vs 84% & 82%), participating in ECE (27% vs 29% & 39%) and developmentally on track according to ECDI score (38% vs 46% & 56%) than in regional and provisional estimates in MICS 2014 and 2019, respectively [16, 34]. The low ECDI scores may be explained by the low proportion of children who are ‘on track’ in the socio-emotional domain, which includes items that may be strongly influenced by social norms that require children to be calm and compliant. Moreover, a recent study on the cognitive testing of ECDI2030 highlights the development and refinement of population-level measures of ECD should be culturally relevant to reduce response bias [35].

Improved feeding and caregiving practices were associated with wealth in our setting. Children from wealthier households were more likely to receive colostrum feeding, be exclusively breastfed for the past 24-hours, achieve MDD (≥4), have access to two or more toys and participate in ECE. Our findings are consistent with other studies that report that children from the poorest wealth quintile were not fed colostrum [25, 36], non-exclusively breastfed [37], and did not meet the MDD [38–42]. Low-income families tend to have limited access to education resources and poor utilisation of health services, affecting IYCF practices. Likewise, poverty is associated with poor child development indicators in low- and middle-income countries (LMICs) [4, 28, 29, 43]. Previous studies in Nepal have found household wealth to be a strong predictor of ECD [27, 44]. However, the adverse effects of poverty on child development can be mitigated through parents’ engagement with their children in cognitive and socio-emotional caregiving practices [45, 46]. Ensuring parents’ awareness and engagement in IYCF and caregiving activities needs to be a programmatic priority [3, 45].

Our study highlights the association of lack of antenatal visits and home delivery among young mothers with poor feeding and caregiving practices. Children of adolescent and young mothers were more likely to have delayed or early introduction of complementary foods, a finding consistent with a previous study from Nepal [38]. Adolescent and young mothers are physiologically and socio-economically disadvantaged because of early marriage and childbearing, with lower education levels, high financial dependency, low self-esteem, and low decision-making autonomy [47]. Despite efforts to reduce child marriage in Nepal, it remains a pervasive public health issue [48], with more than half (52%) of women aged 25–49 having married before the age of 18 and nearly a quarter of them (17%) becoming mothers before the age of 18 [17]. The problem is pronounced in Dhanusha district and other areas of Province 2, where 88% of women aged 20–30 years are married before 18 [49]. Many adolescent mothers have restricted access to maternal health services, impacting their knowledge and awareness of nutrition and caregiving practices. Programmes and policies to strengthen efforts to prevent early marriage and childbearing and improve knowledge and practice about optimal IYCF and caregiving practices among adolescent and young mothers need to be prioritised in this context.

We found that antenatal visits were associated with the timely introduction of complementary foods, as supported by previous studies from Nepal [38, 50], Sri Lanka [51] and Pakistan [52]. More antenatal visits were associated with greater access to three or more children’s books and two or more toys, suggesting that mothers receive information and counselling about their value for ECD during antenatal visits. Alternatively, wealthier, more educated women living in more accessible areas who are more likely to have antenatal care may be able to afford books and toys for their children. Our finding that delivering in a health facility was associated with better breastfeeding practices echoes prior studies from Nepal [26, 53] and India [54]. Improved access to community health care services may enable the transfer of nutrition and caregiving information, especially to young mothers. Hence, existing antenatal, delivery, and postnatal contact points with mothers provide an opportunity to reach out to this vulnerable group at scale and counsel them on optimal feeding and caregiving practices.

In our study, younger children were more likely to fail to achieve MDD and consumed fewer animal-source foods, vegetables, and fruits, as reported in studies from Ethiopia [42] and India [54]. One possible reason for this is the widespread cultural belief in Nepal that cereal foods are sufficient for young children [55]. Furthermore, cultural taboos dictate that children are not offered eggs and flesh foods until they have their first tooth, usually at the age of 1 year [56]. Compared with children aged 43–59 months, younger children were at higher risk of poor access to three or more children’s books, two or more toys, ECE and early stimulation and responsive caregiving, and at higher risk of poor development. This highlights the lack of awareness among parents and caregivers that most development occurs in the early years of life and that optimal feeding and caregiving play a crucial role in promoting ECD. This resonates with prior research from Burkina Faso and Malawi [57, 58], which found a lack of knowledge and awareness among caregivers about the relationship between caregiving practices for younger children and child development.

The association of feeding and caregiving practices with the sex of the child was not in the same direction. Boys were less likely to receive complementary foods at 6–8 months of age, and girls were less likely to have access to two or more toys. Boys, on average, are at higher risk of undernutrition than girls [59, 60]. Lower respiratory infections, diarrhoeal disease, malaria and preterm birth were observed to occur more frequently in boys than in girls [61]. These conditions increase boys’ susceptibility to undernutrition, which may impact the initiation of complementary feeding. Girls’ low access to toys may be explained by their greater engagement in household chores from an early age and parents’ preferential treatment of boys. Other studies showed inconsistent gender differences: Bornstein et al. (2012) found no difference in caregiving practices between genders in 38 LMICs [7], while data across 35 LMICs using the ECDI scores showed that boys scored lower than girls [62]. Further exploration of gender-based feeding and caregiving practices in Nepal is warranted to reduce disparity.

Prior research consistently shows that maternal education is a strong predictor of optimal feeding [38, 40, 54, 63] and caregiving practices [28, 64–66]. The underlying mechanism explaining the association between maternal education and improved child developmental outcomes in LMICs is responsive caregiving behaviour, including early stimulation and learning support [67]. Mothers with more education can foster an enabling environment at home to promote child development [28, 68]. Our finding that maternal education was not a common factor affecting both practices may be explained by the lack of variability in the level of education in our sample, with only 12% having attended secondary school or beyond.

### Strengths and limitations

Our study has some limitations. First, the cross-sectional design precludes causal inferences. Second, while our results shed light on the factors affecting IYCF and caregiving practices in lowland Nepal, our findings may not be generalisable to other areas of Nepal due to cultural and geographical variation. Third, the information on feeding and caregiving practices was self-reported by mothers, which may be liable to recall bias and social desirability bias. Fourth, the developmental potential of children at 36–59 months of age was assessed using the ECDI score, which has limited coverage of cognition and culturally specific developmental milestones [35, 62]. Fifth, we did not analyse the factors affecting maternal and paternal caregiving practices separately. Mothers and fathers play critical but distinct roles in a child’s development. Studies have found that fathers’ engagement in stimulating activities is more influential to child development than mothers’ engagement in similar activities [27, 67]. Similarly, the inclusion of the explanatory variables was limited to those indicators that were available and it is possible that we could have missed other important predictors of IYCF and caregiving*.* Sixth, while we used a wide range of indicators embedded within the *Nurturing Care Framework*, we missed health and safety and security. To address these limitations, future studies should use age-appropriate and culture-sensitive population measures to gauge child development, examine separately the potential factors influencing the parenting practices of both mothers and fathers at different levels in the surrounding environment and explore all aspects of nurturing care to inform a holistic approach to child development. Finally, whilst our data date back to 2012, the gap in IYCF and caregiving practices may still exist; significant interventions, particularly on responsive caregiving and early learning for children under 3 years, have not been implemented in the area since then.

### Recommendations and future directions

Our findings provide context-based evidence to guide the Government of Nepal, international agencies, non-governmental organisations and public health experts to inform the design of interventions to improve ECD. Young children of adolescent and young mothers with limited access to health services during pregnancy and those from low-income families should be targeted for interventions to reduce the gap in child development in lowland Nepal. This strategy must be strengthened at the local level, given the country’s recent transition to a decentralised federal structure. Identifying those at risk of poor feeding and caregiving practices has further highlighted the need for an integrated approach to reduce the adverse consequences of poor practices on ECD. Existing interventions to enhance ECD are delivered in silos or with only partial integration of health and nutrition or nutrition and caregiving interventions [13, 69, 70]. Local, state, and federal governments need to focus on bringing different sectors together and designing interventions that incorporate all aspects of nurturing care for a child’s holistic development. Governments should increase investment in parenting programmes to support parents and caregivers to improve their feeding and caregiving practices for young children to reduce inequalities in child development [12, 71]. Likewise, the Government of Nepal and other key stakeholders should engage in social and behaviour change communication at household and community level to increase awareness of the optimal feeding and caregiving practices that support ECD.

## Conclusions

Our study demonstrates that sub-optimal caregiving practices, inappropriate early breastfeeding practices, delayed introduction of complementary foods, inadequate dietary diversity and low animal-source food consumption are challenges in lowland Nepal and call for urgent integrated nutrition and caregiving interventions, especially as interventions to promote ECD are lacking. To optimise feeding and caregiving practices in lowland Nepal, we encourage health service providers, policymakers and public health researchers to focus on young children in their first 3 years of life, particularly those from disadvantaged groups, and to improve health services for adolescent and young mothers. A holistic approach to child development should be prioritised to support parents and caregivers in resource-constrained settings to help improve their understanding of feeding and caregiving practices and promote ECD.

## Supplementary Information

**Additional file 1: Supplementary Figure 1.** Flow chart of study participants. **Supplementary Figure 2.** Multivariable modelling procedure. **Supplementary Figure 3.** Complementary feeding practices in the past 24-hours by age when children were 7 to 59 months. **Supplementary Figure 4.** Percentage of children aged 7 to 59 months who experienced types of learning activities with different caregivers *other adults refers to maternal and paternal grandparents. **Supplementary Figure 5.** Early childhood index score by gender and wealth index when children were 36 -59 months.

**Additional file 2: Supplementary Table 1.** A summary of indicators available from each of 6-week questionnaire and the follow-up survey sample.

**Additional file 3: Supplementary Table 2.** The timing of the data collection for each of the IYCF indicators and the age range.

**Additional file 4: Supplementary Table 3.** Definition and categorisation of potential explanatory variables used in the study.

**Additional file 5: Supplementary Table 4.** Multivariable logistic regression analysis of factors associated with timely initiation of breastfeeding (recall of the first days of life) among children aged 0–12 months. **Supplementary Table 5.** Multivariable logistic regression analysis of factors associated with colostrum feeding (recall of the first days of life) among children aged 0–12 months. **Supplementary Table 6.** Multivariable logistic regression analysis of factors associated with no pre-lacteal feeding (recall of the first days of life) among children aged 0–12 months. **Supplementary Table 7.** Multivariable logistic regression analysis of factors associated with exclusive breastfeeding in past 24 hours before survey among children aged 0–5 months. **Supplementary Table 8.** Multivariable logistic regression analysis of factors associated with timing of the introduction of solid, semi-solid or soft foods among children aged 7–59 months. **Supplementary Table 9.** Multivariable logistic regression analysis of factors associated with minimum dietary diversity among children aged 7–59 months. **Supplementary Table 10.** Multivariable logistic regression analysis of factors associated with consumption of animal foods among children aged 7–59 months. **Supplementary Table 11.** Multivariable logistic regression analysis of factors associated with consumption of fruits and vegetables among children aged 7–59 months.

**Additional file 6: Supplementary Table 12.** Multivariable logistic regression analysis of factors associated with access to three or more books among children aged 7–59. **Supplementary Table 13.** Multivariable logistic regression analysis of factors associated with access to two or more toys among children aged 7–59. **Supplementary Table 14.** Multivariable logistic regression analysis of factors associated with early stimulation and responsive caregiving by mother/father/other adults to children aged 24–59 months. **Supplementary Table 15.** Multivariable logistic regression analysis of factors associated with adequate supervision of children aged 7–59 months. **Supplementary Table 16.** Multivariable logistic regression analysis of factors associated with attendance of early childhood education by children aged 36–59 months. **Supplementary Table 17.** Multivariable logistic regression analysis of factors associated with developmentally on track according to ECDI score of children aged 36–59 months.

**Additional file 7: Supplementary Table 18.** Matrix summarising the multivariable analysis in the Model 3 for each indicator*.

## Data Availability

The data supporting this study’s findings are available from the corresponding author upon reasonable request.

## Abbreviations

AOR: Adjusted Odds Ratio
cRCT: cluster Randomised Controlled Trial
CI: Confidence Interval
ECD: Early Childhood Development
ECDI: Early Child Development Index
ECE: Early Childhood Education
IYCF: Infant and Young Child Feeding
LMIC: Low-and Middle-Income Countries
MDD: Minimum Dietary Diversity
PLA: Participatory Learning and Action
MAHFP: Months of Adequate Household Food Provisioning
MICS: Multiple Indicator Cluster Survey
MIRA: Mother and Infant Research Activities
NDHS: Nepal Demographic Health Survey
UNICEF: United Nations Children Fund’s
VDC: Village Development Committees
WHO: World Health Organization

## References

[1] Shonkoff JP, Richter L, van der Gaag J, Bhutta ZA (2012). An integrated scientific framework for child survival and early childhood development. Pediatrics.

[2] Britto PR, Lye SJ, Proulx K, Yousafzai AK, Matthews SG, Vaivada T (2017). Nurturing care: promoting early childhood development. Lancet.

[3] Black MM, Walker SP, Fernald LCH, Andersen CT, DiGirolamo AM, Lu C (2017). Early childhood development coming of age: science through the life course. Lancet.

[4] Grantham-McGregor S, Cheung YB, Cueto S, Glewwe P, Ritcher L, Strupp B (2007). Developmental potential in the first 5 years for children in developing countries. Lancet.

[5] Walker SP, Wachs TD, Grantham-McGregor S, Black MM, Nelson CA, Huffman SL (2011). Child development: risk factors for adverse outcomes in developing countries. Lancet.

[6] Indicators for assessing infant and young child feeding practices: definitions and measurement methods. Geneva: World Health Organization and the United Nations Children’s Fund (UNICEF); 2021. Licence: CC BY- NC-SA 3.0 IGO. Available from: https://creativecommons.org/licenses/by-nc-sa/3.0/igo. Cited 2021 Oct 4.

[7] Bornstein MH, Putnick DL (2012). Cognitive and socioemotional caregiving in developing countries. Child Dev.

[8] Bornstein MH, Putnick DL, Lansford JE, Deater-Deckard K, Bradley RH (2015). A developmental analysis of caregiving modalities across infancy in 38 low- and middle-income countries. Child Dev.

[9] Dulal S, Prost A, Karki S, Saville N, Merom D (2021). Characteristics and effects of integrated nutrition and stimulation interventions to improve the nutritional status and development of children under 5 years of age: a systematic review and meta-analysis. BMJ Glob Health.

[10] World Health Organization (2020). Improving early childhood development: WHO guideline.

[11] Prado EL, Larson LM, Cox K, Bettencourt K, Kubes JN, Shankar AH (2019). Do effects of early life interventions on linear growth correspond to effects on neurobehavioural development? A systematic review and meta-analysis. Lancet Glob Health.

[12] Engle PL, Fernald LCH, Alderman H, Behrman J, O’Gara C, Yousafzai A (2011). Strategies for reducing inequalities and improving developmental outcomes for young children in low-income and middle-income countries. Lancet.

[13] Oxford Policy Management (2018). Evaluation of the national early childhood development program.

[14] National Planning Commission (2017). Multi-sector nutrition plan: 2018–2022.

[15] Helen Keller International (2020). Suaahara II Good Nutrition Program: Annual Survey year three (2019).

[16] Government of Nepal, National Planning Commission & Central Bureau of Statistics. Monitoring the situation of children and women: Multiple Indicator Cluster Survey 2019. UNICEF; 2020.

[17] Ministry of Health (2017). Nepal; New ERA; and ICF. Nepal Demographic and Health Survey 2016.

[18] Central Bureau of Statistics (2012). National Population and housing census 2011, National Report.

[19] Shrestha BP, Bhandari B, Manandhar DS, Osrin D, Costello A, Saville N (2011). Community interventions to reduce child mortality in Dhanusha, Nepal: study protocol for a cluster randomised controlled trial. Trials.

[20] Indicators for assessing infant and young child feeding practices: Part 1 definitions. Geneva: World Health Organization and the United Nations Children’s Fund (UNICEF), 2008. Available from: http://apps.who.int/iris/bitstream/handle/10665/43895/9789241596664_eng.pdf;jsessionid=B4EBBA0A8270C6B67155BC9179B4FFB7?sequence=1. Cited 2021 August 21.

[21] United Nations Children’s Fund (2019). The formative years: UNICEF’s work on measuring early childhood development.

[22] Modjadji P, Molokwane D, Ukegbu PO (2020). Dietary diversity and nutritional status of preschool children in north West Province, South Africa: a cross sectional study. Children.

[23] Busert BK, Neuman M, Rehfuess EA, Dulal S, Harthan J, Chaube SS (2016). Dietary diversity is positively associated with deviation from expected height in rural Nepal. J Nutr.

[24] Khanal V, Sauer K, Zhao Y (2013). Determinants of complementary feeding practices among Nepalese children aged 6–23 months: findings from demographic and health survey. 2011. BMC Pediatrics.

[25] Bhandari S, Thorne-Lyman AL, Shrestha B, Neupane S, Nonyane BAS, Manohar S (2019). Determinants of infant breastfeeding practices in Nepal: a national study. Int Breastfeed J.

[26] Acharya P, Khanal V (2015). The effect of mother’s educational status on early initiation of breastfeeding: further analysis of three consecutive Nepal demographic and health surveys. BMC Public Health.

[27] Rayhan S, Banerjee A, Mishra R, Barua S. Quality of care and early childhood developmental status in Nepal: a multilevel analysis. Early Child Dev Care. 2019;1-14. 10.1080/03004430.2019.1570503.

[28] Sun J, Liu Y, Chen EE, Rao N, Liu H (2016). Factors related to parents’ engagement in cognitive and socio-emotional caregiving in developing countries: results from multiple Indicator cluster survey 3. Early Child Res Q.

[29] Tran TD, Luchters S, Fisher J (2017). Early childhood development: impact of national human development, family poverty, parenting practices and access to early childhood education. Child Care Health Dev.

[30] Pandey S, Tiwari K, Upul S, Agho KE, Dibley MJ (2010). Determinants of infant and young child feeding practices in Nepal: secondary data analysis of demographic and health survey 2006. Food Nutr Bull.

[31] Rutstein SO, Johnson K (2004). The DHS wealth index.

[32] Hosmer DW, Lemeshow S, Sturdivant RX (2013). Applied logistic regression.

[33] Cunningham K, Headey D, Singh A, Karmacharya C, Rana PP (2017). Maternal and child nutrition in Nepal: examining drivers of progress from the mid-1990s to 2010s. Global. Food Security.

[34] Central Bureau of Statistics (2015). Nepal multiple Indicator cluster survey 2014: final report.

[35] Cappa C, Petrowski N, De Castro EF, Geisen E, LeBaron P, Allen-Leigh B (2021). Identifying and minimizing errors in the measurement of early childhood development: lessons learned from the cognitive testing of the ECDI2030. Int J Environ Res Public Health.

[36] Kumar A, Unisa S, Sharma B (2017). Infant and young child feeding practices in India: a comparison of empowered action group (EAG) and non-EAG states. Soc Sci Spectrum.

[37] Khatun H, Comins CA, Shah R, Munirul Islam M, Choudhury N, Ahmed T (2018). Uncovering the barriers to exclusive breastfeeding for mothers living in Dhaka’s slums: a mixed-method study. Int Breastfeed J.

[38] Na M, Aguayo VM, Arimond M, Dahal P, Lamichhane B, Pokharel R (2018). Trends and predictors of appropriate complementary feeding practices in Nepal: an analysis of national household survey data collected between 2001 and 2014. Matern Child Nutr.

[39] Baek Y, Chitekwe S (2019). Socio-demographic factors associated with inadequate food group consumption and dietary diversity among infants and young children in Nepal. Plos One.

[40] Ali NB, Tahsina T, Hoque DME, Hasan MM, Iqbal A, Huda TM (2019). Association of food security and other socio-economic factors with dietary diversity and nutritional statuses of children aged 6-59 months in rural Bangladesh. PLoS One.

[41] Shaker-Berbari L, Qahoush Tyler V, Akik C, Jamaluddine Z, Ghattas H. Predictors of complementary feeding practices among children aged 6–23 months in five countries in the Middle East and North Africa region. Matern Child Nutr. 2021:e13223. 10.1111/mcn.13223. 10.1111/mcn.13223 PMC8476411 34137179

[42] Woldegebriel AG, Desta AA, Gebreegziabiher G, Berhe AA, Ajemu KF, Woldearegay TW (2020). Dietary diversity and associated factors among children aged 6-59 months in Ethiopia: analysis of Ethiopian demographic and health survey 2016 (EDHS 2016). Int J Pediatr.

[43] Lu C, Cuartas J, Fink G, McCoy D, Liu K, Li Z (2020). Inequalities in early childhood care and development in low/middle-income countries: 2010-2018. BMJ Glob Health.

[44] Patel SA, Murray-Kolb LE, LeClerq SC, Khatry SK, Tielsch JM, Katz J (2013). Household wealth and neurocognitive development disparities among school-aged children in Nepal. Pediatr Perinat Epidemiol.

[45] Sun J, Lau C, Sincovich A, Rao N (2018). Socioeconomic status and early child development in East Asia and the Pacific: the protective role of parental engagement in learning activities. Child Youth Serv Rev.

[46] Frongillo EA, Kulkarni S, Basnet S, de Castro F (2017). Family care behaviors and early childhood development in low- and middle-income countries. J Child Fam Stud.

[47] Shahabuddin ASM, Delvaux T, Nöstlinger C, Sarker M, Bardají A, Sharkey A (2019). Maternal health care-seeking behaviour of married adolescent girls: a prospective qualitative study in Banke District, Nepal. Plos One.

[48] Scott S, Nguyen PH, Neupane S, Pramanik P, Nanda P, Bhutta ZA (2021). Early marriage and early childbearing in South Asia: trends, inequalities, and drivers from 2005 to 2018. Ann N Y Acad Sci.

[49] Marphatia AA, Saville NM, Manandhar DS, Cortina-Borja M, Reid AM, Wells JCK (2021). Independent associations of women’s age at marriage and first pregnancy with their height in rural lowland Nepal. Am J Phys Anthropol.

[50] Joshi N, Agho KE, Dibley MJ, Senarath U, Tiwari K (2012). Determinants of inappropriate complementary feeding practices in young children in Nepal: secondary data analysis of demographic and health survey 2006. Matern Child Nutr.

[51] Senarath U, Godakandage SSP, Jayawickrama H, Siriwardena I, Dibley MJ (2012). Determinants of inappropriate complementary feeding practices in young children in Sri Lanka: secondary data analysis of demographic and health survey 2006–2007. Matern Child Nutr.

[52] Na M, Aguayo VM, Arimond M, Stewart CP (2017). Risk factors of poor complementary feeding practices in Pakistani children aged 6–23 months: a multilevel analysis of the demographic and health survey 2012–2013. Matern Child Nutr.

[53] Pagel C, Prost A, Hossen M, Azad K, Kuddus A, Roy SS (2014). Is essential newborn care provided by institutions and after home births? Analysis of prospective data from community trials in rural South Asia. BMC Pregnancy Childbirth.

[54] Dhami MV, Ogbo FA, Diallo TMO, Olusanya BO, Goson PC, Agho KE (2021). On behalf of the global maternal and child Health Research collaboration (GloMACH). Infant and young child feeding practices among adolescent mothers and associated factors in India. Nutrients.

[55] Gautam KP, Adhikari M, Khatri RB, Devkota MD (2016). Determinants of infant and young child feeding practices in Rupandehi. Nepal BMC Research Notes.

[56] Locks LM, Pandey PR, Osei AK, Spiro DS, Adhikari DP, Haselow NJ (2015). Using formative research to design a context-specific behaviour change strategy to improve infant and young child feeding practices and nutrition in Nepal. Matern Child Nutr.

[57] Hollowell J, Dumbaugh M, Belem M, Kousse S, Swigart T, Korsaga C (2019). ‘Grandmother, aren’t you going to sing for us?’ Current childcare practices and caregivers’ perceptions of and receptivity to early childhood development activities in rural Burkina Faso. BMJ Glob Health.

[58] Gladstone M, Phuka J, Mirdamadi S, Chidzalo K, Chitimbe F, Koenraads M, et al. The care, stimulation and nutrition of children from 0-2 in Malawi-perspectives from caregivers; “Who’s holding the baby?”. Plos One. 2018;13(6):e0199757. 10.1371/journal.pone.0199757. 10.1371/journal.pone.0199757 PMC6021079 29949636

[59] Thurstans S, Opondo C, Seal A, Wells J, Khara T, Dolan C (2020). Boys are more likely to be undernourished than girls: a systematic review and meta-analysis of sex differences in undernutrition. BMJ Glob Health.

[60] Saville NM, Harris- Fry H, Marphatia A, Reid A, Cortina-Borja M, Manandhar DS, et al. Differences in maternal and early child nutritional status by offspring sex in lowland Nepal. Am J Hum Biol. 2021:e23637. 10.1002/ajhb.23637. 10.1002/ajhb.23637 PMC12086752 34228379

[61] Hawkes S, Buse K (2013). Gender and global health: evidence, policy, and inconvenient truths. Lancet.

[62] McCoy DC, Peet ED, Ezzati M, Danaei G, Black MM, Sudfeld CR (2016). Early childhood developmental status in low- and middle-income countries: national, regional, and global prevalence estimates using predictive modeling. Plos Med.

[63] Neves PAR, Barros AJD, Gatica-Domínguez G, Vaz JS, Baker P, Lutter CK (2021). Maternal education and equity in breastfeeding: trends and patterns in 81 low- and middle-income countries between 2000 and 2019. Int J Equity Health.

[64] Zhang C, Zhao C, Liu X, Wei Q, Luo S, Guo S (2017). Inequality in early childhood neurodevelopment in six poor rural counties of China: a decomposition analysis. Int J Equity Health.

[65] Mazharul Islam M, Khan JR, Kabir A, Rahman Khan MZ, Islam M (2021). M associations of socio-demographic and environmental factors with the early development of young children in Bangladesh. International journal of early. Childhood.

[66] Donald KA, Wedderburn CJ, Barnett W, Nhapi RT, Rehman AM, Stadler JAM (2019). Risk and protective factors for child development: an observational south African birth cohort. PLoS Med.

[67] Jeong J, McCoy DC, Fink G (2017). Paternal and maternal education, caregivers’ support for learning, and early child development in 44 low- and middle- income countries. Early Child Res Q.

[68] Cuartas J, Jeong J, Rey-Guerra C, McCoy DC, Yoshikawa H (2020). Maternal, paternal, and other caregivers’ stimulation in low- and- middle-income countries. Plos One.

[69] Aboud FE, Yousafzai AK (2015). Global health and development in early childhood. Annu Rev Psychol.

[70] Grantham-McGregor S, Fernald LC, Kagawa RM, Walker S (2014). Effects of integrated child development and nutrition interventions on child development and nutritional status. Ann N Y Acad Sci.

[71] Jeong J, Franchett EE, Ramos de Oliveira CV, Rehmani K, Yousafzai AK (2021). Parenting interventions to promote early child development in the first three years of life: a global systematic review and meta-analysis. Plos Med.

