# Supplementary material for: Feeding, caregiving practices, and developmental delay among children under five in lowland Nepal: a community-based cross-sectional survey
Source: BMC Public Health. 2022 Sep 10;22:1721. doi: 10.1186/s12889-022-13776-8 (PMC9464411; doi:10.1186/s12889-022-13776-8)
Supplement: Supplementary file 1 — Additional file 1: Supplementary Figure 1. Flow chart of study participants. Supplementary Figure 2. Multivariable modelling procedure. Supplementary Figure 3. Complementary feeding practices in the past 24-hours by age when children were 7 to 59 months. Supplementary Figure 4. Percentage of children aged 7 to 59 months who experienced types of learning activities with different caregivers *other adults refers to maternal and paternal grandparents. Supplementary Figure 5. Early childhood index score by gender and wealth index when children were 36 -59 months. [file 12889_2022_13776_MOESM1_ESM.pdf]

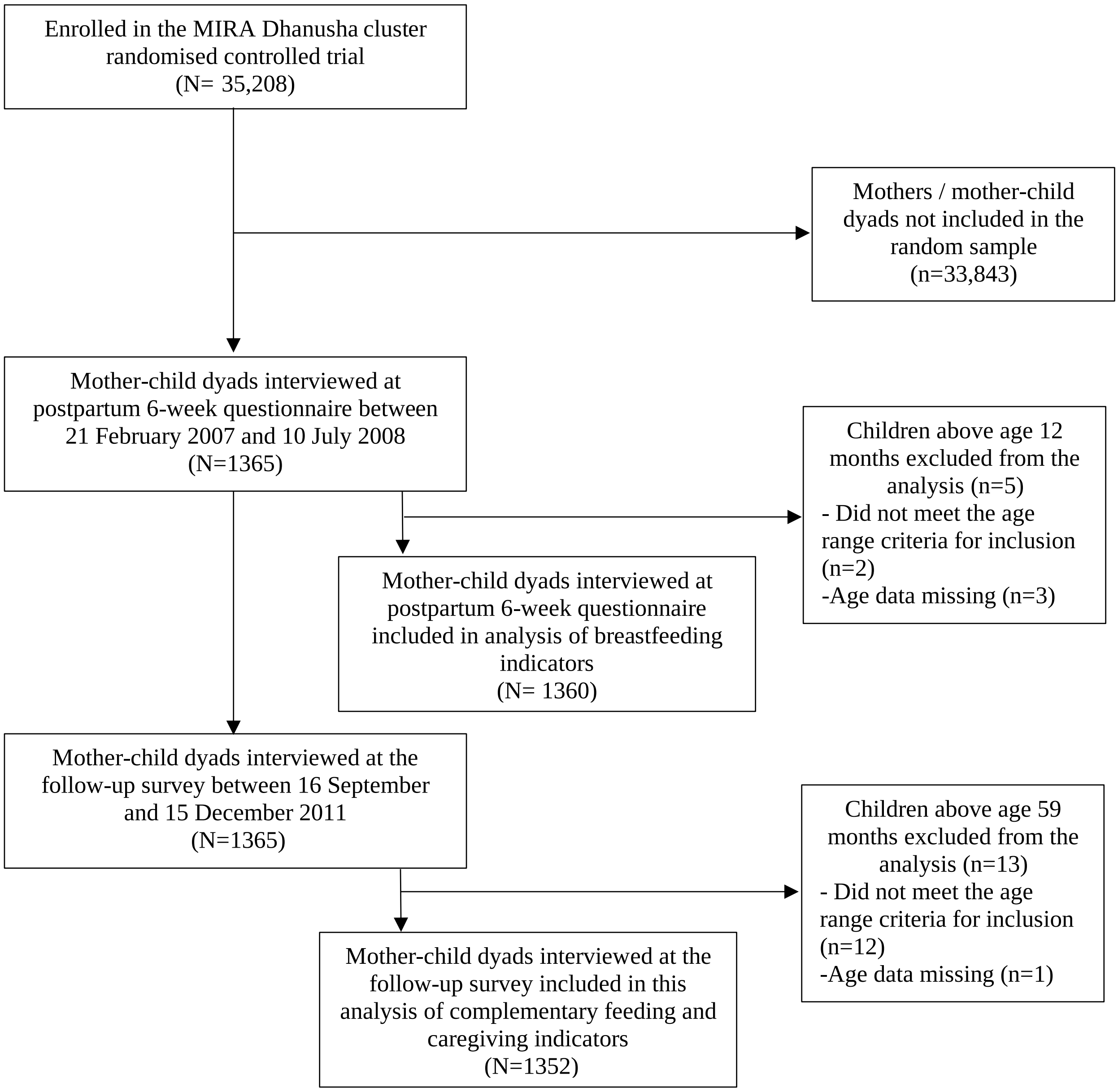

Supplementary figure 1 Flow chart of study participants

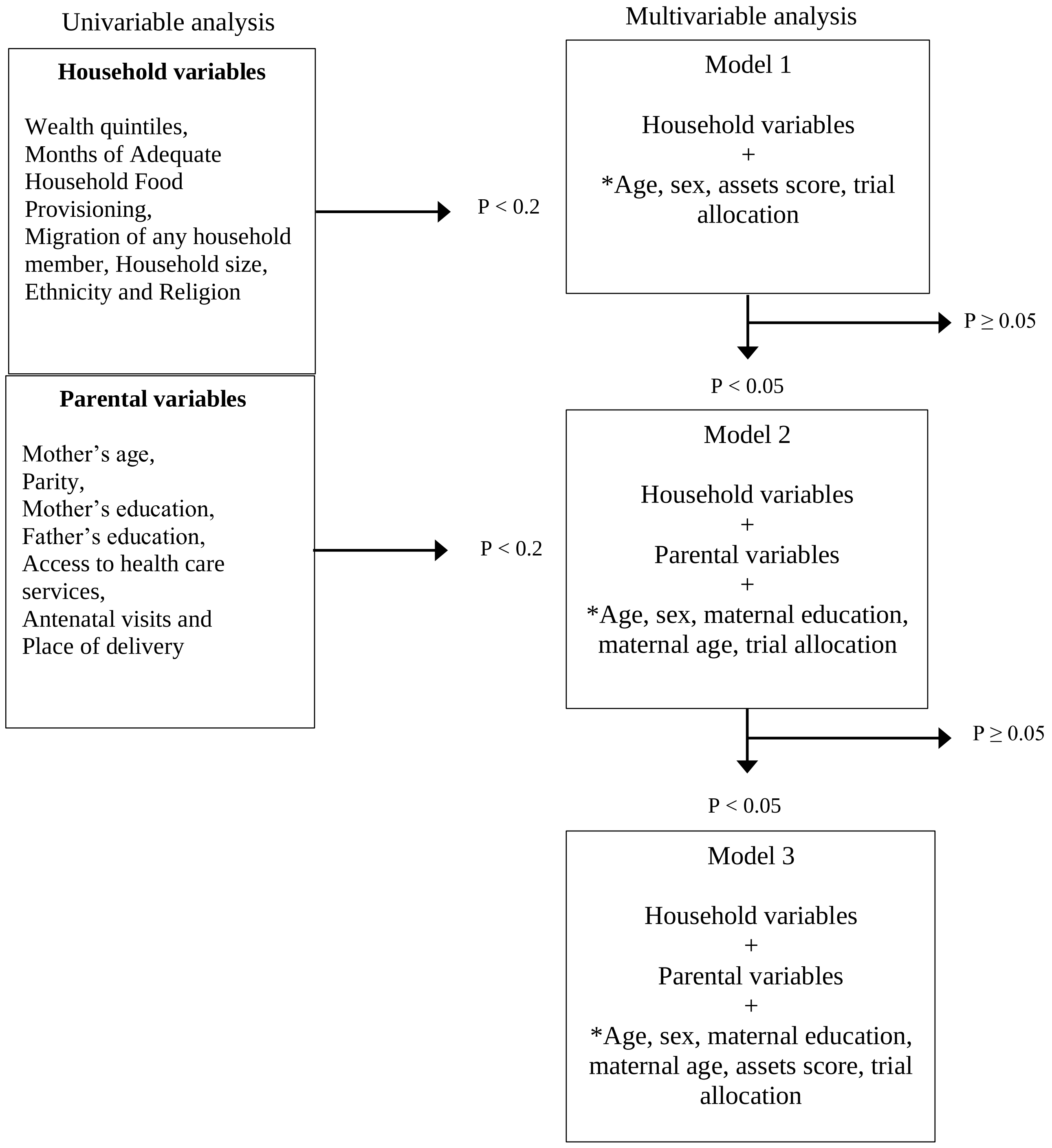

Supplementary figure 2: Multivariable modelling procedure

Model 1 (M1) includes all the *pre-identified (household-level) variables + household-level variables with $p < 0.2$ in univariable analysis; Model 2 (M2) includes all the *pre-identified (parental-level) variables + parental-level variables with $p < 0.2$ in univariable analyses + all the pre-identified (household-level) variables from M1+ household-level variables with $p < 0.05$ in M1; Model 3 (final model) includes all the *pre-identified (household or parental level) variables + parental-level and household-level variables with $p < 0.05$ in M2.

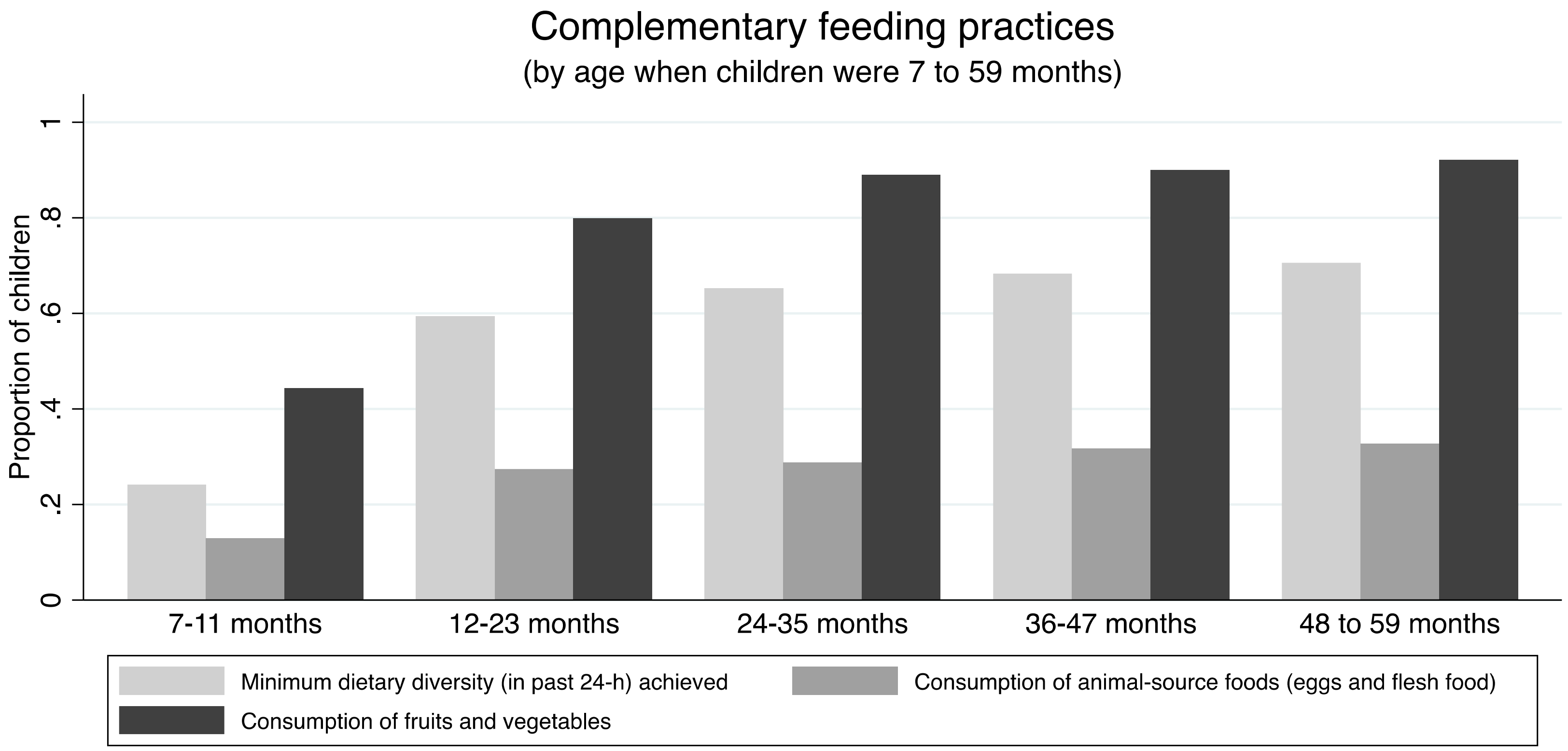

Supplementary figure 3: Complementary feeding practices in the past 24-hours by age when children were 7 to 59 months

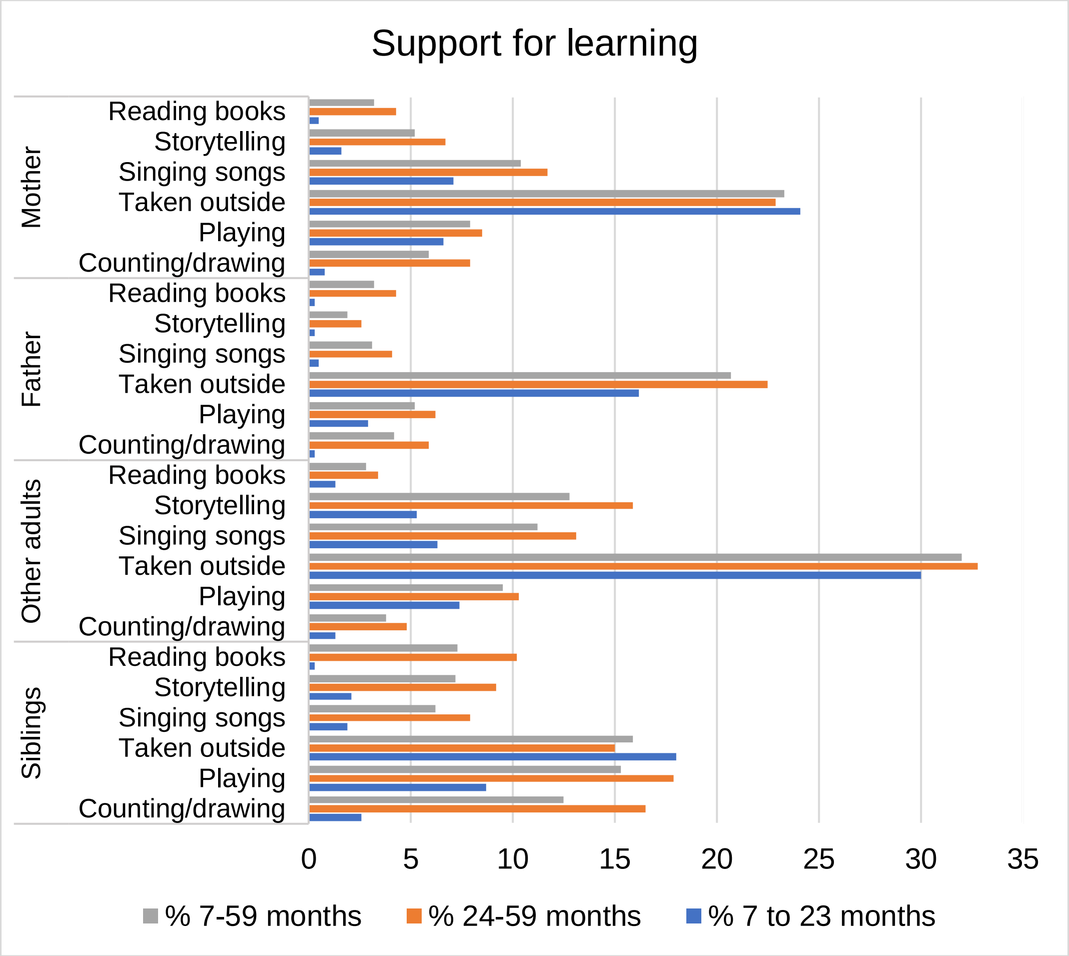

Supplementary figure 4: Percentage of children aged 7 to 59 months who experienced types of learning activities with different caregivers
*other adults refers to maternal and paternal grandparents.

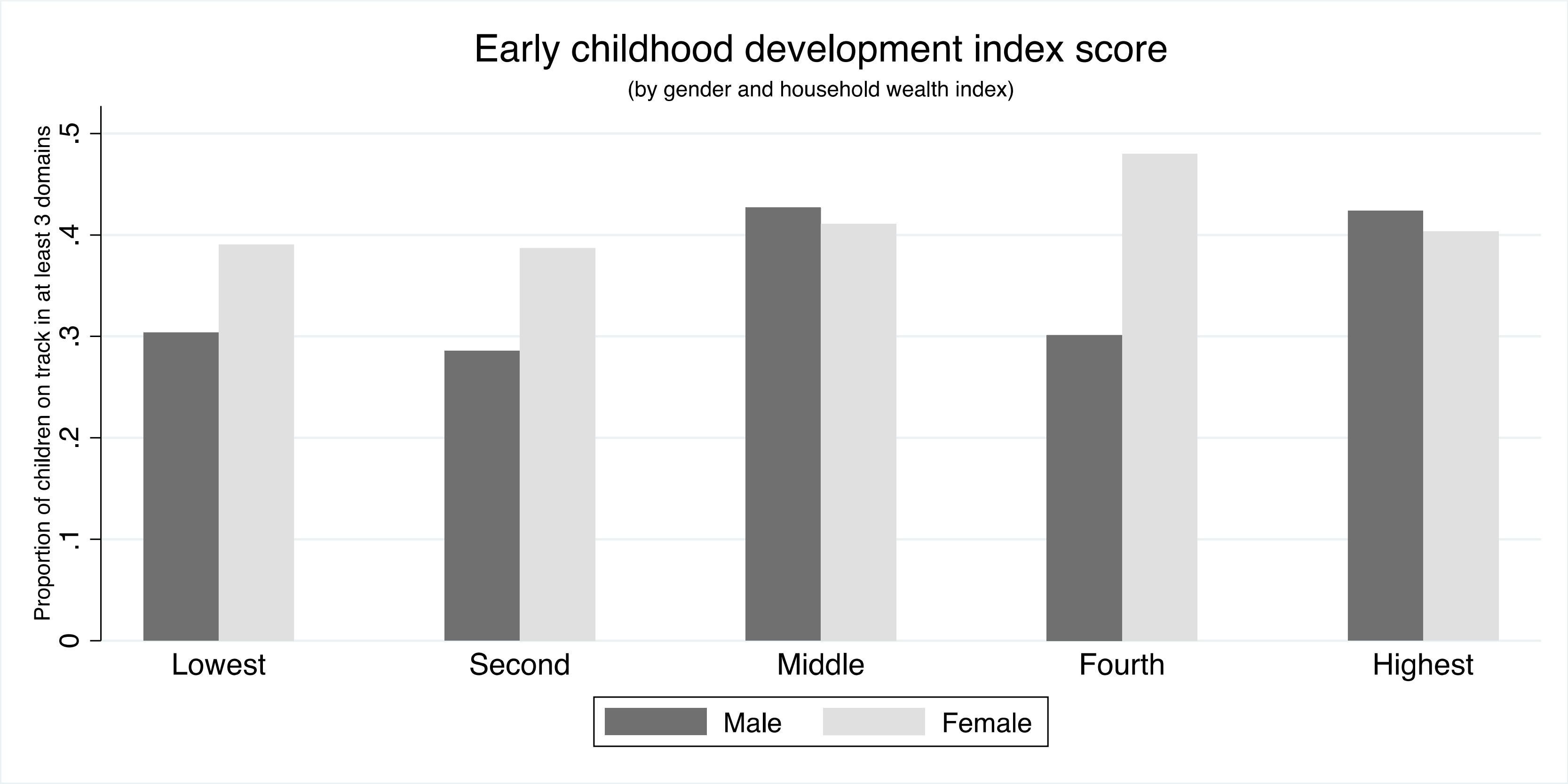

Supplementary figure 5: Early childhood index score by gender and wealth index when children were 36 -59 months
