# Supplementary material for: Feeding, caregiving practices, and developmental delay among children under five in lowland Nepal: a community-based cross-sectional survey
Source: BMC Public Health. 2022 Sep 10;22:1721. doi: 10.1186/s12889-022-13776-8 (PMC9464411; doi:10.1186/s12889-022-13776-8)
Supplement: Supplementary file 2 — Additional file 2: Supplementary Table 1. A summary of indicators available from each of 6-week questionnaire and the follow-up survey sample. [file 12889_2022_13776_MOESM2_ESM.pdf]

Supplementary table 1: A summary of indicators available from each of 6-week questionnaire and the follow-up survey sample

**6-week questionnaire (21 February 2007 - 10 July 2008)**			**Follow-up survey (16 September - 15 December 2011)**		
**Children aged 0-12 months**			**Children aged 7-59 months**		
**Exploratory variables**	**Breastfeeding indicators**		**Exploratory variables**	**Complementary feeding indicators**	**Cognitive and socio-emotional caregiving indicators**
Child's age	Ever breastfed		Child's age	Minimum dietary diversity in last 24 hours	Access to three or more children's books
Wealth index	Timing of breastfeeding Initiation		Child's Sex	Consumption of any animal foods in last 24 hours	Access to two or more toys
Months of Adequate Household Food Provisioning (MAHFP)	Exclusive breastfeeding in past 24 hours			Consumption of fruits and vegetables in last 24 hours	Adequate supervision
Migration of any household member	Colostrum feeding				Stimulation by mother, father, or other adult
Household size	No pre-lacteal feeding				Attendance of early childhood education
Access to health care services					Early Childhood Development Index scores
Ethnicity					
Religion					
Mother's age					
Mother's education					
Father's education					
Parity					
Antenatal visits					
Place of delivery					
