# Supplementary material for: Feeding, caregiving practices, and developmental delay among children under five in lowland Nepal: a community-based cross-sectional survey
Source: BMC Public Health. 2022 Sep 10;22:1721. doi: 10.1186/s12889-022-13776-8 (PMC9464411; doi:10.1186/s12889-022-13776-8)
Supplement: Supplementary file 3 — Additional file 3: Supplementary Table 2. The timing of the data collection for each of the IYCF indicators and the age range. [file 12889_2022_13776_MOESM3_ESM.pdf]

Supplementary table 2: The timing of the data collection for each of the IYCF indicators and the age range

Year	WHO indicator	WHO definition	Suggested age group for WHO indicator	Indicator used in our study	Time in the life course in our study	Age group suggested to sample for WHO indicators	Age group sampled in our study	n in the 6-week questionnaire of children aged 0-12 months	n in follow up survey of children aged 7 to 59 months
**BREASTFEEDING INDICATORS**									
**2021**	Ever breastfed	Percentage of children born in the last 24 months who were ever breastfed	Children born in the last 24 months	Ever breastfed	First 24 months but we have recall from first 6 weeks mostly	0 to 23 months	0 to 12 months (median age 46 days)	1358	
**2021**	Early initiation of breastfeeding	Percentage of children born in the last 24 months who were put to the breast within one hour of birth	Children born in the last 24 months	Detailed timing of breastfeeding initiation	First 3 days of life			1358	
				Colostrum feeding				1343	
				Pre-lacteal feeding				1342	
**2021**	Exclusively breastfed for the first two days after birth	Percentage of children born in the last 24 months who were fed exclusively with breast milk for the first two days after birth	Children born in the last 24 months	Exclusive breastfeeding for last 24 hours amongst children birth to 182 days at interview	Up to 6 months		0 to 5 months (median age 45 days)	1313	
						0 to 5 months			
**COMPLEMENTARY FEEDING INDICATORS**									
**2021**	Introduction of solid, semi-solid or soft foods 6–8 months	Percentage of infants 6–8 months of age who consumed solid, semi-solid or soft foods during the previous day	Children 6–23 months of age	Percentage of children group who were introduced solid-semi-solid or soft foods between 6-to-8-month age (recall for all children sampled)	6 months up to 1 year	6 to 23 months	7 to 59 months (ALL recalling the past) (median age 38 months)		1313

Year	WHO indicator	WHO definition	Suggested age group for WHO indicator	Indicator used in our study	Time in the life course in our study	Age group suggested to sample for WHO indicators	Age group sampled in our study	n in the 6-week questionnaire of children aged 0-12 months	n in follow up survey of children aged 7 to 59 months
**2008**	Minimum dietary diversity 6–23 months	Percentage of children 6–23 months of age who consumed foods and beverages from at least four out of seven defined food groups during the previous day	Children 6–23 months of age	Minimum dietary diversity achieved for children during the previous day	6 to 23 months but we have data 7 to 59 months	6 to 23 months	7 to 59 months (median age 38 months)		1352
**2021**	Egg and/or flesh food consumption 6–23 months	Percentage of children 6–23 months of age who consumed egg and/or flesh food during the previous day	Children 6–23 months of age	Consumption of animal foods (eggs and flesh food) during the previous day		6 to 23 months			1336
**2021**	Zero vegetable or fruit consumption 6–23 months	Percentage of children 6–23 months of age who did not consume any vegetables or fruits during the previous day	Children 6–23 months of age	Consumption of any fruits and vegetables during the previous day		6 to 23 months			1336
