# Supplementary material for: Feeding, caregiving practices, and developmental delay among children under five in lowland Nepal: a community-based cross-sectional survey
Source: BMC Public Health. 2022 Sep 10;22:1721. doi: 10.1186/s12889-022-13776-8 (PMC9464411; doi:10.1186/s12889-022-13776-8)
Supplement: Supplementary file 4 — Additional file 4: Supplementary Table 3. Definition and categorisation of potential explanatory variables used in the study. [file 12889_2022_13776_MOESM4_ESM.pdf]

Supplementary table 3 Definition and categorisation of potential explanatory variables used in the study

Variable	Variable definition	Variable type	Category
**Household level factors**			
**Wealth quintiles**	Asset based wealth quintile using DHS (PCA) method. (Quintiles were generated using the first principal component: highest quintile represents the wealthiest households).	Categorical	1 = Lowest; 2 = Second; 3 = Middle; 4 = Fourth; 5 = Highest
**Months of Adequate Household Food Provisioning (MAHFP)**	The food security in the 12-month period before main surveillance	Categorical	1 = For up to 7 months; 2 = For 8 to 11 months; 3 = For 12 months
**Migration of at least one household member**	Migration of any household member outside the country	Binary	1 = Yes; 0 = No
**Household size**	Number of people (adults and children) residing in the same HH grouped into categories	Categorical	1 = 1-5 members; 2 = 6-10 members; 3 = ≥11 members
**Access to health care services**	Child was seen/taken to health facility during the recall period	Binary	1= Has access; 0 = Does not have access
**Caste group**	Caste groups based on the socioeconomic status	Categorical	1 = Dalit/Muslim (most disadvantaged group); 2 = Janajati/other terai cast (Sudi/Teli); 3 = Yadav/Brahmin (least disadvantaged group)
**Religion**	Religion of the household	Binary	0 = Hindu; 1 = non-Hindu (mostly Muslims)
**Parent level factors**			
**Mother's age**	Mothers age during the follow-up survey	Categorical	1 = 15-24; 2 = 25-34; 3 = 5- 45 years
**Mother's education**	Education level of the child's mother	Categorical	0 = Never went to school; 1 = Primary; 2 = Secondary and above
**Mother's literacy**	Maternal reading ability	Binary	1 = Can read easily; 0 = Cannot read or with some difficulty
**Father's education**	Education level of the child's mother	Categorical.	0 = Never went to school; 1 = Primary; 2 = Secondary and above

**Parity**	Total number previous live births and stillbirths including the index child	Categorical	1 = One; 2 = Two; 3 = Three; 4 = Four or more
**Antenatal visits**	Total number of antenatal checkups by health professional	Categorical	0 = None; 1 = 1-3 visits; 2 = 4+
**Place of delivery**	Place of the delivery	Binary	0 = Home; 1 = Health facility
**Child level factors**			
**Age in days at 6-week interview**	Collected in the post-neonatal period when the index child was median 46 days old.	Categorical	1= <46 days; 2 = 46-91 days; 3 = 92-182 days (3-5 months); 4 = 183-366 days (6-12 months)
**Age in months at follow-up study**	Collected in the follow-up cross-sectional survey questionnaire when the child was median age 38 months.	Categorical	1 = 7-11 months; 2 = 12-23 months; 3 = 24-35 months; 4 = 36-37 months; 5 = 48-59 months
**Sex**	Sex of the child	Binary	1 = Female; 0 = Male
