# Supplementary material for: Feeding, caregiving practices, and developmental delay among children under five in lowland Nepal: a community-based cross-sectional survey
Source: BMC Public Health. 2022 Sep 10;22:1721. doi: 10.1186/s12889-022-13776-8 (PMC9464411; doi:10.1186/s12889-022-13776-8)
Supplement: Supplementary file 5 — Additional file 5: Supplementary Table 4. Multivariable logistic regression analysis of factors associated with timely initiation of breastfeeding (recall of the first days of life) among children aged 0–12 months. Supplementary Table 5. Multivariable logistic regression analysis of factors associated with colostrum feeding (recall of the first days of life) among children aged 0–12 months. Supplementary Table 6. Multivariable logistic regression analysis of factors associated with no pre-lacteal feeding (recall of the first days of life) among children aged 0–12 months. Supplementary Table 7. Multivariable logistic regression analysis of factors associated with exclusive breastfeeding in past 24 hours before survey among children aged 0–5 months. Supplementary Table 8. Multivariable logistic regression analysis of factors associated with timing of the introduction of solid, semi-solid or soft foods among children aged 7–59 months. Supplementary Table 9. Multivariable logistic regression analysis of factors associated with minimum dietary diversity among children aged 7–59 months. Supplementary Table 10. Multivariable logistic regression analysis of factors associated with consumption of animal foods among children aged 7–59 months. Supplementary Table 11. Multivariable logistic regression analysis of factors associated with consumption of fruits and vegetables among children aged 7–59 months. [file 12889_2022_13776_MOESM5_ESM.pdf]

Supplementary table 4: Multivariable logistic regression analysis of factors associated with timely initiation of breastfeeding (recall of the first days of life) among children aged 0-12 months

Indicators	Initiated breastfeed-ing within one hour (N=215) n (%)	Initiated breastfeed-ing after one hour (N=1135) n (%)	Unadjusted OR (95% CI)	*p* value	Model 1 AOR (95% CI)	*p* value	Model 2 AOR (95% CI)	*p* value	Model 3 AOR (95% CI)	*p* value
**HOUSEHOLD CHARACTERISTICS**										
Wealth Quintile										
Lowest (ref)	29 (11.9)	215 (88.1)	1		1					
Second	37 (14.3)	221 (85.7)	1.30 (0.74, 2.26)	0.360	1.27 (0.69, 2.36)	0.441				
Middle	49 (16.7)	244 (83.3)	1.56 (0.92, 2.64)	0.097	1.49 (0.80, 2.78)	0.208				
Fourth	44 (18.1)	199 (81.9)	1.62 (0.94, 2.79)	0.082	1.41 (0.72, 2.76)	0.321				
Highest	48 (18.4)	213 (81.6)	**1.79 (1.05, 3.07)**	**0.033**	1.60 (0.79, 3.24)	0.190				
Months of adequate food provisioning (MAHFP)										
For up to 7 months	20 (11.7)	151 (88.3)	0.69 (0.40, 1.17)	0.169	1.00 (0.52, 1.90)	0.994				
For 8 to 11 months	53 (17.0)	258 (83.0)	0.96 (0.65, 1.41)	0.833	1.08 (0.69, 1.71)	0.732				
For 12 months (ref)	140 (16.2)	722 (83.8)	1		1					
Migration of at least one household member										
No (ref)	105 (16.2)	543 (83.8)	1							
Yes	109 (15.7)	584 (84.3)	0.93 (0.68, 1.27)	0.642						
Household size										
1-5 members	45 (14.5)	266 (85.5)	0.77 (0.52, 1.34)	0.186	0.77 (0.50, 1.18)	0.234				
6-10 members (ref)	140 (17.4)	663 (82.6)	1		1					
>=11 members	29 (12.9)	195 (87.1)	0.70 (0.44, 1.11)	0.131	0.72 (0.44, 1.17)	0.184				
Healthcare access										
No (ref)	166 (15.4)	912 (84.6)	1		1		1			
Yes	39 (22.4)	135 (77.6)	**1.80 (1.17, 2.76)**	**0.008**	**1.85 (1.17, 2.93)**	**0.009**	1.55 (0.97, 2.48)	0.066		
Ethnicity/Caste										
Dalit/ Muslim (ref)	55 (13.1)	365 (86.9)	1		1					
Janjati/other terai caste	95 (15.0)	540 (85.0)	1.11 (0.75, 1.64)	0.599	0.88 (0.53, 1.46)	0.621				
Yadav/ Brahmin	65 (22.0)	230 (78.0)	**1.79 (1.16, 2.76)**	**0.009**	1.42 (0.80, 2.53)	0.235				
Religion										
Non-Hindu	17 (10.8)	141 (89.2)	0.67 (0.38, 1.17)	0.158	0.69 (0.33, 1.41)	0.305				
Hindu (ref)	198 (16.6)	994 (83.4)	1		1					
**PARENTAL CHARACTERISTICS**										
Maternal age										
15-24 years (ref)	82 (15.8)	436 (84.2)	1				1		1	
25-34 years	117 (16.0)	614 (84.0)	1.01 (0.72, 1.40)	0.968			0.84 (0.49, 1.42)	0.508	0.88 (0.53, 1.46)	0.617
35-45 years	16 (16.0)	84 (84.0)	1.01 (0.54, 1.89)	0.977			0.66 (0.27, 1.60)	0.358	0.61 (0.26, 1.43)	0.252
No of previous pregnancies										
One (ref)	63 (14.7)	366 (85.3)	1				1		1	
Two	68 (18.5)	299 (81.5)	1.23 (0.82, 1.84)	0.324			**1.76 (1.04, 3.00)**	**0.037**	1.67 (1.00, 2.79)	0.050
Three	30 (11.7)	227 (88.3)	0.76 (0.46, 1.24)	0.267			1.46 (0.72, 2.94)	0.293	1.30 (0.67, 2.55)	0.439

Indicators	Initiated breastfeed-ing within one hour (N=215) n (%)	Initiated breastfeed-ing after one hour (N=1135) n (%)	Unadjusted OR (95% CI)	*p* value	Model 1 AOR (95% CI)	*p* value	Model 2 AOR (95% CI)	*p* value	Model 3 AOR (95% CI)	*p* value
Four or more	54 (18.2)	242 (81.8)	1.41 (0.92, 2.15)	0.116			**2.71 (1.33, 5.52)**	**0.006**	**2.66 (1.35, 5.23)**	**0.005**
Maternal education										
Never went to school (ref)	172 (15.7)	922 (84.3)	1				1		1	
Primary	20 (20.2)	79 (79.8)	1.22 (0.70, 2.13)	0.479			1.26 (0.68, 2.33)	0.460	1.19 (0.66, 2.16)	0.561
Secondary or above	23 (14.6)	134 (85.4)	0.91 (0.55, 1.50)	0.708			1.00 (0.58, 1.73)	0.991	0.98 (0.58, 1.66)	0.951
Father's education										
Never went to school (ref)	134 (15.5)	730 (84.5)	1							
Primary	23 (14.5)	136 (85.5)	0.98 (0.59, 1.63)	0.931						
Secondary or above	58 (17.7)	269 (82.3)	1.18 (0.82, 1.70)	0.370						
Antenatal visits										
None (ref)	59 (13.6)	374 (86.4)	1				1			
1-3 visits	97 (15.2)	542 (84.8)	1.08 (0.74, 1.57)	0.703			1.05 (0.69, 1.61)	0.805		
4+ visits	59 (21.2)	219 (78.8)	**1.63 (1.06, 2.50)**	**0.025**			1.12 (0.67, 1.86)	0.674		
Place of delivery										
Home (ref)	123 (11.9)	914 (88.1)	1				1		1	
Health facility	88 (30.7)	199 (69.3)	**3.85 (2.69, 5.51)**	**<0.001**			**4.51 (2.99, 6.81)**	**<0.001**	**4.53 (3.09, 6.65)**	**<0.001**
**CHILD CHARACTERISTICS**										
Child sex										
Male (ref)	116 (16.2)	599 (83.8)	1		1		1		1	
Female	99 (15.6)	536 (84.4)	0.97 (0.71, 1.33)	0.860	1.04 (0.74, 1.46)	0.825	0.98 (0.69, 1.38)	0.894	0.98 (0.71, 1.37)	0.923
Child age at recall	**(Median, IQR)**	**(Median, IQR)**								
Age in months	1.5 (1.1-2.1)	1.5 (1.1-2.2)	**0.86 (0.76, 0.98)**	**0.021**	0.88 (0.77, 1.00)	0.051	**0.84 (0.74, 0.97)**	**0.015**	**0.85 (0.75, 0.98)**	**0.020**

For interpretation purposes, OR >1 indicates children are more likely to be breastfed within an hour of birth and OR<1 indicates children are less likely.
Model 1 (N= 1196) included child sex, trial allocation as a priori covariates plus wealth quintile, MAHFP, HH size, health care access, ethnicity, and religion from unadjusted analysis with a p<0.2.
Model 2 (N=1226) included maternal age, maternal education, child sex, trial allocation as a priori covariates plus parity, antenatal visits, place of delivery from unadjusted analysis with a p<0.2. plus, health care access from Model 1 with p<0.05.
Model 3 (N=1322) included maternal age, maternal education, child sex, trial allocation as a priori covariates plus parity and place of delivery from Model 2 with p<0.05.
Abbreviation: OR Odds Ratio, AOR Adjusted Odds Ratio, IQR Interquartile range

Supplementary table 5: Multivariable logistic regression analysis of factors associated with colostrum feeding (recall of the first days of life) among children aged 0-12 months

Indicators	Colostrum fed (N=894) n (%)	Colostrum not fed (N=449) n (%)	Unadjusted OR (95% CI)	*p* value	Model 1 AOR (95% CI)	*p* value	Model 2 AOR (95% CI)	*p* value	Model 3 AOR (95% CI)	*p* value
**HOUSEHOLD CHARACTERISTICS**										
Wealth Quintile										
Lowest (ref)	138 (56.3)	107 (43.7)	1		1		1		1	
Second	165 (64.0)	93 (36.0)	1.35 (0.92, 1.99)	0.121	1.30 (0.87, 1.94)	0.202	1.20 (0.81, 1.79)	0.360	1.20 (0.81, 1.78)	0.354
Middle	195 (67.2)	95 (32.8)	**1.68 (1.16, 2.45)**	**0.006**	**1.58 (1.05, 2.37)**	**0.029**	1.42 (0.96, 2.10)	0.075	1.45 (0.98, 2.13)	0.062
Fourth	174 (73.1)	64 (26.9)	**2.08 (1.39, 3.13)**	**<0.001**	**1.88 (1.19, 2.97)**	**0.006**	**1.70 (1.10, 2.61)**	**0.017**	**1.69 (1.10, 2.60)**	**0.016**
Highest	188 (72.0)	73 (28.0)	**2.13 (1.43, 3.17)**	**<0.001**	**2.02 (1.27, 3.22)**	**0.003**	1.44 (0.93, 2.24)	0.106	1.44 (0.93, 2.22)	0.103
Months of adequate food provisioning (MAHFP)										
For up to 7 months	97 (57.1)	73 (42.9)	**0.60 (0.41, 0.87)**	**0.007**	0.90 (0.58, 1.40)	0.648				
For 8 to 11 months	193 (62.3)	117 (37.7)	**0.67 (0.50, 0.90)**	**0.009**	0.85 (0.61, 1.20)	0.356				
For 12 months (ref)	600 (70.0)	257 (30.0)	1		1					
Migration of at least one household member										
No (ref)	427 (65.9)	221 (34.1)	1							
Yes	462 (67.2)	225 (32.8)	1.05 (0.82, 1.33)	0.722						
Household size										
1-5 members	208 (67.1)	102 (32.9)	0.98 (0.73, 1.32)	0.894						
6-10 members (ref)	539 (67.3)	262 (32.7)	1							
>=11 members	140 (63.1)	82 (36.9)	0.89 (0.64, 1.24)	0.479						
Healthcare access										
No (ref)	711 (66.2)	363 (33.8)	1							
Yes	118 (67.8)	56 (32.2)	1.12 (0.78, 1.62)	0.531						
Ethnicity/Caste										
Dalit/ Muslim (ref)	276 (66.3)	140 (33.7)	1							
Janjati/other terai caste	410 (64.5)	226 (35.5)	0.91 (0.69, 1.21)	0.531						
Yadav/ Brahmin	208 (71.5)	83 (28.5)	1.19 (0.83, 1.70)	0.335						
Religion										
Non-Hindu	101 (65.2)	54 (34.8)	1.00 (0.68, 1.47)	0.983						
Hindu (ref)	793 (66.8)	395 (33.2)	1							
**PARENTAL CHARACTERISTICS**										
Maternal age										
15-24 years (ref)	354 (68.5)	163 (31.5)	1				1		1	
25-34 years	477 (65.7)	249 (34.3)	0.90 (0.69, 1.16)	0.400			0.96 (0.65, 1.42)	0.842	1.02 (0.78, 1.35)	0.870
35-45 years	62 (62.6)	37 (37.4)	0.79 (0.49, 1.28)	0.340			0.86 (0.44, 1.67)	0.659	1.05 (0.63, 1.76)	0.858
No of previous pregnancies										
One (ref)	298 (69.8)	129 (30.2)	1				1			
Two	240 (65.9)	124 (34.1)	0.90 (0.65, 1.23)	0.500			1.05 (0.71, 1.56)	0.808		
Three	161 (62.9)	95 (37.1)	0.74 (0.52, 1.04)	0.083			1.00 (0.61, 1.62)	0.989		
Four or more	194 (65.8)	101 (34.2)	0.88 (0.63, 1.23)	0.449			1.28 (0.76, 2.16)	0.342		

Indicators	Colostrum fed (N=894) n (%)	Colostrum not fed (N=449) n (%)	Unadjusted OR (95% CI)	*p* value	Model 1 AOR (95% CI)	*p* value	Model 2 AOR (95% CI)	*p* value	Model 3 AOR (95% CI)	*p* value
Maternal education										
Never went to school (ref)	707 (65.0)	381 (35.0)	1				1		1	
Primary	70 (71.4)	28 (28.6)	1.48 (0.91, 2.41)	0.112			1.33 (0.79, 2.21)	0.281	1.30 (0.78, 2.17)	0.316
Secondary or above	117 (74.5)	40 (25.5)	**1.64 (1.10, 2.46)**	**0.015**			1.39 (0.89, 2.18)	0.151	1.41 (0.90, 2.20)	0.134
Father's education										
Never went to school (ref)	570 (66.2)	291 (33.8)	1							
Primary	106 (66.7)	53 (33.3)	1.01 (0.69, 1.48)	0.963						
Secondary or above	218 (67.5)	105 (32.5)	1.06 (0.79, 1.42)	0.699						
Antenatal visits										
None (ref)	265 (61.2)	168 (38.8)	1				1			
1-3 visits	436 (68.4)	201 (31.6)	**1.37 (1.04, 1.80)**	**0.026**			1.23 (0.91, 1.66)	0.173		
4+ visits	193 (70.7)	80 (29.3)	**1.48 (1.04, 2.10)**	**0.027**			1.00 (0.67, 1.49)	0.992		
Place of delivery										
Home (ref)	643 (62.3)	389 (37.7)	1				1		1	
Health facility	233 (81.5)	53 (18.5)	**2.79 (1.98, 3.95)**	**<0.001**			**2.76 (1.88, 4.06)**	**<0.001**	**2.68 (1.85, 3.88)**	**<0.001**
**CHILD CHARACTERISTICS**										
Child sex										
Male (ref)	468 (65.7)	244 (34.3)	1		1		1		1	
Female	426 (67.5)	205 (32.5)	1.09 (0.85, 1.39)	0.491	1.10 (0.85, 1.42)	0.466	1.10 (0.85, 1.42)	0.475	1.10 (0.85, 1.42)	0.483
Child age at recall	**(Median, IQR)**	**(Median, IQR)**								
Age in months	1.5 (1.1-2.1)	1.6 (1.1-2.4)	0.93 (0.86, 1.01)	0.075	**0.92 (0.85, 1.00)**	**0.048**	**0.92 (0.84, 1.00)**	**0.045**	**0.92 (0.84, 1.00)**	**0.038**

For interpretation purposes, OR >1 indicates children are more likely to be fed colostrum and OR<1 indicates children are less likely.
Model 1 (N= 1287) included child sex, trial allocation as a priori covariates plus wealth, MAHFP from unadjusted analysis with a p<0.2.
Model 2 (N=1268) included maternal age, maternal education, child sex, trial allocation as a priori covariates plus parity, antenatal visits, place of delivery from unadjusted analysis with a p<0.2 plus, wealth from Model 1 with p<0.05.
Model 3 (N=1268) included maternal age, maternal education, child sex, trial allocation as a priori covariates plus wealth, place of delivery from Model 2 with p<0.05.
Abbreviation: OR Odds Ratio, AOR Adjusted Odds Ratio, IQR Interquartile range

Supplementary table 6: Multivariable logistic regression analysis of factors associated with no pre-lacteal feeding (recall of the first days of life) among children aged 0-12 months

**Indicators**	**Yes - no pre-lacteal feeding (N=715) n (%)**	**No - pre-lacteal feeding (N=627) n (%)**	**Unadjusted OR (95% CI)**	***p* value**	**Model 1 AOR (95% CI)**	***p* value**	**Model 2 AOR (95% CI)**	***p* value**	**Model 3 AOR (95% CI)**	***p* value**
**HOUSEHOLD CHARACTERISTICS**										
Wealth Quintile										
Lowest (ref)	122 (50.6)	119 (49.4)	1		1					
Second	125 (48.4)	133 (51.6)	0.92 (0.64, 1.34)	0.676	0.88 (0.60, 1.30)	0.533				
Middle	159 (54.6)	132 (45.4)	1.19 (0.83, 1.71)	0.347	1.12 (0.76, 1.66)	0.573				
Fourth	125 (51.5)	117 (48.3)	1.01 (0.70, 1.48)	0.945	0.96 (0.63, 1.46)	0.853				
Highest	156 (59.5)	106 (40.5)	1.40 (0.97, 2.04)	0.075	1.34 (0.87, 2.07)	0.187				
Months of adequate food provisioning (MAHFP)										
For up to 7 months	85 (50.0)	85 (50.0)	0.84 (0.58, 1.19)	0.325	1.01 (0.66, 1.53)	0.970				
For 8 to 11 months	154 (49.8)	155 (50.2)	0.80 (0.61, 1.06)	0.128	0.95 (0.69, 1.30)	0.735				
For 12 months (ref)	472 (55.1)	385 (44.9)	1		1					
Migration of at least one household member										
No (ref)	332 (51.4)	314 (48.6)	1		1					
Yes	377 (54.9)	310 (45.1)	1.22 (0.97, 1.53)	0.091	1.21 (0.95, 1.53)	0.120				
Household size										
1-5 members	174 (56.7)	133 (43.3)	1.14 (0.87, 1.51)	0.327						
6-10 members (ref)	419 (52.4)	380 (47.6)	1							
>=11 members	117 (52.2)	107 (47.8)	1.13 (0.73, 1.36)	0.981						
Healthcare access										
No (ref)	562 (52.4)	510 (47.6)	1							
Yes	101 (58.0)	73 (42.0)	1.11 (0.78, 1.57)	0.557						
Ethnicity/Caste										
Dalit/ Muslim (ref)	216 (51.8)	201 (48.2)	1							
Janjati/other terai caste	332 (52.5)	300 (47.5)	0.99 (0.76, 1.30)	0.961						
Yadav/ Brahmin	167 (57.0)	126 (43.0)	1.23 (0.89, 1.70)	0.213						
Religion										
Non-Hindu	77 (48.4)	82 (51.6)	0.85 (0.59, 1.21)	0.357						
Hindu (ref)	638 (53.9)	545 (46.1)	1							
**PARENTAL CHARACTERISTICS**										
Maternal age										
15-24 years (ref)	301 (58.3)	215 (41.7)	1				1		1	
25-34 years	375 (51.7)	350 (48.3)	**0.77 (0.60, 0.97)**	**0.029**			0.85 (0.60, 1.22)	0.390	0.92 (0.72, 1.19)	0.542
35-45 years	39 (39.0)	61 (61.0)	**0.44 (0.28, 0.70)**	**0.001**			**0.43 (0.23, 0.79)**	**0.007**	**0.50 (0.31, 0.81)**	**0.005**
No of previous pregnancies										
One (ref)	247 (57.6)	182 (42.4)	1				1			
Two	199 (54.8)	164 (45.2)	0.88 (0.66, 1.19)	0.421			1.20 (0.83, 1.72)	0.337		
Three	126 (50.2)	125 (49.8)	**0.70 (0.50, 0.97)**	**0.031**			1.03 (0.66, 1.63)	0.883		
Four or more	143 (47.8)	156 (52.2)	**0.69 (0.51, 0.95)**	**0.021**			1.28 (0.79, 2.05)	0.317		

Indicators	Yes - no pre-lacteal feeding (N=715) n (%)	No - pre-lacteal feeding (N=627) n (%)	Unadjusted OR (95% CI)	*p* value	Model 1 AOR (95% CI)	*p* value	Model 2 AOR (95% CI)	*p* value	Model 3 AOR (95% CI)	*p* value
Maternal education										
Never went to school (ref)	572 (52.6)	515 (47.4)	1				1		1	
Primary	59 (60.2)	39 (39.8)	1.38 (0.89, 2.16)	0.154			1.23 (0.77, 1.95)	0.389	1.24 (0.78,1.96)	0.366
Secondary or above	84 (53.5)	73 (46.5)	1.00 (0.71, 1.43)	0.978			0.92 (0.63, 1.34)	0.667	0.93 (0.64, 1.35)	0.707
Father's education										
Never went to school (ref)	465 (54.0)	396 (46.0)	1							
Primary	83 (53.2)	73 (46.8)	1.02 (0.71, 1.46)	0.925						
Secondary or above	167 (51.4)	158 (48.6)	0.85 (0.65, 1.12)	0.248						
Antenatal visits										
None (ref)	208 (48.4)	222 (51.6)	1				1			
1-3 visits	331 (52.1)	304 (47.9)	1.07 (0.82, 1.39)	0.616			0.95 (0.72, 1.25)	0.693		
4+ visits	176 (63.5)	101 (36.5)	**1.72 (1.24, 2.39)**	**0.001**			1.23 (0.86, 1.77)	0.260		
Place of delivery										
Home (ref)	492 (47.7)	540 (52.3)	1				1		1	
Health facility	208 (73.0)	77 (27.0)	**2.99 (2.20, 4.06)**	**<0.001**			**2.90 (2.09, 4.03)**	**<0.001**	**3.02 (2.20, 4.14)**	**<0.001**
**CHILD CHARACTERISTICS**										
Child sex										
Male (ref)	372 (51.9)	345 (48.1)	1		1		1		1	
Female	343 (54.9)	282 (45.1)	1.15 (0.92, 1.44)	0.228	1.17 (0.92, 1.48)	0.192	1.19 (0.94, 1.51)	0.147	1.18 (0.93, 1.50)	0.164
Child age at recall	**(Median, IQR)**	**(Median, IQR)**								
Age in months	1.4 (1.1-2.1)	1.5 (1.1-2.4)	**0.89 (0.82, 0.96)**	**0.003**	**0.88 (0.81, 0.96)**	**0.002**	**0.87 (0.81, 0.95)**	**0.001**	**0.87 (0.80, 0.95)**	**0.001**

For interpretation purposes, OR >1 indicates children are more likely to not be given pre-lacteal feeding and OR<1 indicates children are less likely.

Model 1 (N= 1289) included child sex, trial allocation as a priori covariates plus wealth, MAHFP, migration of HH member from unadjusted analysis with a p<0.2.

Model 2 (N=1316) included maternal age, maternal education, child sex, trial allocation as a priori covariates plus parity, antenatal visits, place of delivery from unadjusted analysis with a p<0.2.

Model 3 (N=1316) included maternal age, maternal education, child sex, trial allocation as a priori covariates plus place of delivery from Model 2 with p<0.05.

Abbreviation: OR Odds Ratio, AOR Adjusted Odds Ratio, IQR Interquartile range

Supplementary table 7: Multivariable logistic regression analysis of factors associated with exclusive breastfeeding in past 24 hours before survey among children aged 0-5 months

**Indicators**	**Yes - exclusively breastfed (N=1173) n (%)**	**Not exclusively breastfed (N=141) n (%)**	**Unadjusted OR (95% CI)**	***p* value**	**Model 1 AOR (95% CI)**	***p* value**	**Model 2 AOR (95% CI)**	***p* value**	**Model 3 AOR (95% CI)**	***p* value**
**HOUSEHOLD CHARACTERISTICS**										
Wealth Quintile										
Lowest (ref)	200 (86.6)	31 (13.4)	1		1		1		1	
Second	227 (89.4)	27(10.6)	1.55 (0.86, 2.81)	0.148	1.74 (0.92, 3.29)	0.089	1.70 (0.90, 3.21)	0.100	1.61 (0.86, 3.00)	0.135
Middle	250 (88.3)	33 (11.7)	1.20 (0.68, 2.11)	0.536	1.25 (0.66, 2.38)	0.491	1.24 (0.68, 2.26)	0.484	1.17 (0.65, 2.13)	0.597
Fourth	212 (88.7)	27 (11.3)	1.38 (0.76, 2.51)	0.294	1.73 (0.85, 3.50)	0.130	1.88 (0.96, 3.69)	0.067	1.63 (0.85, 3.13)	0.138
Highest	239 (93.4)	17 (6.6)	**2.27 (1.18, 4.39)**	**0.015**	**2.48 (1.12, 5.47)**	**0.025**	**2.83 (1.34, 6.00)**	**0.006**	**2.58 (1.24, 5.39)**	**0.012**
Months of adequate food provisioning (MAHFP)										
For up to 7 months	146 (90.1)	16 (9.9)	0.89 (0.48, 1.63)	0.696	1.18 (0.57, 2.46)	0.657				
For 8 to 11 months	256 (85.6)	43 (14.4)	**0.63 (0.41, 0.98)**	**0.042**	0.78 (0.47, 1.32)	0.347				
For 12 months (ref)	766 (90.3)	82 (9.7)	1		1					
Migration of at least one household member										
No (ref)	570 (89.2)	69 (10.8)	1							
Yes	596 (89.5)	70 (10.5)	1.04 (0.71, 1.52)	0.841						
Household size										
1-5 members	272 (89.5)	32 (10.5)	1.15 (0.73, 1.81)	0.554	1.15 (0.70, 1.89)	0.586				
6-10 members (ref)	690 (88.6)	89 (11.4)	1		1					
>=11 members	200 (91.3)	19 (8.7)	0.51 (0.87, 2.63)	0.143	1.27 (0.70, 2.32)	0.430				
Healthcare access										
No (ref)	945 (89.7)	108 (10.3)	1		1		1		1	
Yes	135 (82.8)	28 (17.2)	**0.53 (0.32, 0.87)**	**0.013**	**0.50 (0.29, 0.85)**	**0.011**	**0.50 (0.10, 0.87)**	**0.013**	**0.50 (0.29, 0.85)**	**0.011**
Ethnicity/Caste										
Dalit/ Muslim (ref)	367 (90.0)	41 (10.0)	1							
Janjati/other terai caste	544 (88.2)	73 (11.8)	0.84 (0.54, 1.30)	0.425						
Yadav/ Brahmin	262 (90.7)	27 (9.3)	1.00 (0.58, 1.75)	0.988						
Religion										
Non-Hindu	133 (86.4)	21 (13.6)	0.82 (0.47, 1.41)	0.473						
Hindu (ref)	1040 (89.7)	120 (10.3)	1							
**PARENTAL CHARACTERISTICS**										
Maternal age										
15-24 years (ref)	450 (89.6)	52 (10.4)	1				1		1	
25-34 years	643 (90.3)	69 (9.7)	1.14 (0.76, 1.71)	0.531			0.81 (0.43, 1.50)	0.501	1.17 (0.75, 1.81)	0.495
35-45 years	80 (80.8)	19 (19.2)	**0.47 (0.25, 0.90)**	**0.022**			**0.30 (0.11, 0.80)**	**0.015**	0.51 (0.25, 1.04)	0.065
No of previous pregnancies										
One (ref)	370 (88.7)	47 (11.3)	1	1			1			
Two	314 (88.2)	42 (11.8)	1.02 (0.64, 1.64)	0.926			1.21 (0.65, 2.26)	0.549		
Three	228 (92.3)	19 (7.7)	1.59 (0.88, 2.86)	0.124			1.80 (0.80, 4.03)	0.158		
Four or more	260 (88.7)	33 (11.3)	1.07 (0.64, 1.77)	0.800			1.90 (0.84,4.28)	0.126		

Indicators	Yes - exclusively breastfed (N=1173) n (%)	Not exclusively breastfed (N=141) n (%)	Unadjusted OR (95% CI)	*p* value	Model 1 AOR (95% CI)	*p* value	Model 2 AOR (95% CI)	*p* value	Model 3 AOR (95% CI)	*p* value
Maternal education										
Never went to school (ref)	941 (88.8)	119 (11.2)	1				1		1	
Primary	89 (90.8)	9 (9.2)	1.22 (0.58, 2.58)	0.605			1.18 (0.49, 2.83)	0.712	1.13 (0.47, 2.68)	0.787
Secondary or above	143 (91.7)	13 (8.3)	1.28 (0.68, 2.41)	0.448			1.01(0.49, 2.08)	0.980	0.96 (0.47, 1.97)	0.910
Father's education										
Never went to school (ref)	758 (90.2)	82 (9.8)	1				1			
Primary	132 (85.2)	23 (14.8)	0.67 (0.39, 1.16)	0.153			086 (0.47, 1.58)	0.627		
Secondary or above	283 (88.7)	36 (11.3)	0.84 (0.54, 1.31)	0.455			0.73 (0.44, 1.21)	0.228		
Antenatal visits										
None (ref)	376 (89.5)	44 (10.5)	1							
1-3 visits	556 (89.1)	68 (10.9)	0.93 (0.60, 1.43)	0.736						
4+ visits	241 (89.3)	29 (10.7)	1.02 (0.59, 1.75)	0.943						
Place of delivery										
Home (ref)	891 (88.4)	117 (11.6)	1							
Health facility	259 (92.2)	22 (7.8)	1.35 (0.81, 2.25)	0.249						
**CHILD CHARACTERISTICS**										
Child sex										
Male (ref)	622 (88.2)	83 (11.8)	1		1		1		1	
Female	551 (90.5)	58 (9.5)	1.27 (0.87, 1.86)	0.218	1.22 (0.80, 1.86)	0.352	1.28 (0.83, 1.96)	0.260	1.25 (0.82, 1.91)	0.299
Child age at recall	**(Median, IQR)**	**(Median, IQR)**								
Age in months	1.5 (1.1-2.1)	1.5 (1.1-2.2)	0.99 (0.81, 1.20)	0.880	0.95 (0.76, 1.18)	0.639	0.95 (0.76, 1.19)	0.655	0.95 (0.76, 1.19)	0.648

For interpretation purposes, OR >1 indicates children are more likely to exclusive breastfeeding in the past 24 hours before survey and OR<1 indicates children are less likely.
Model 1 (N= 1162) included child sex, child age, trial allocation as a priori covariates plus wealth, MAHFP, HH size, health care access from unadjusted analysis with a p<0.2.
Model 2 (N=1173) included maternal age, maternal education, child sex, child age, trial allocation as a priori covariates plus parity, father's education from unadjusted analysis with a p<0.2. plus, wealth from Model 1 with p<0.05.
Model 3 (N= 1173) included maternal age, maternal education, child sex, child age, trial allocation as a priori covariates plus wealth and health care access from Model 2 with p<0.05.
Abbreviation: OR Odds Ratio, AOR Adjusted Odds Ratio, IQR Interquartile range

Supplementary table 8: Multivariable logistic regression analysis of factors associated with timing of the introduction of solid, semi-solid or soft foods among children aged 7-59 months

Indicators	Fed between 6-8 months (N=731) n (%)	Not fed at 6-8 months (N=582) n (%)	Unadjusted OR (95% CI)	*p* value	Model 1 AOR (95% CI)	*p* value	Model 2 AOR (95% CI)	*p* value	Model 3 AOR (95% CI)	*p* value
**HOUSEHOLD CHARACTERISTICS**										
Wealth Quintile										
Lowest (ref)	131 (18.7)	104 (18.4)	1							
Second	134 (19.1)	115 (20.4)	0.88 (0.60, 1.30)	0.525						
Middle	152 (21.7)	134 (23.8)	0.86 (0.59, 1.24)	0.410						
Fourth	130 (18.6)	108 (19.1)	0.95 (0.65, 1.40)	0.804						
Highest	153 (21.9)	103 (18.3)	1.21 (0.82, 1.78)	0.341						
Months of adequate food provisioning (MAHFP)										
For up to 7 months	91 (12.5)	72 (12.4)	1.03 (0.71, 1.49)	0.895	0.97 (0.66, 1.41)	0.866				
For 8 to 11 months	157 (21.6)	145 (25.0)	0.82 (0.62, 1.10)	0.188	0.82 (0.60, 1.10)	0.181				
For 12 months (ref)	480 (65.9)	363 (62.6)	1		1					
Migration of at least one household member										
No (ref)	362 (49.9)	270 (46.6)	1		1					
Yes	364 (50.1)	309 (53.4)	0.84 (0.66, 1.06)	0.138	0.83 (0.65, 1.06)	0.14				
Household size										
1-5 members	186 (25.7)	115 (19.9)	**1.50 (1.12, 2.00)**	**0.006**	**1.48 (1.10, 1.99)**	**0.009**	**1.53 (1.14, 2.05)**	**0.005**	**1.54 (1.15, 2.06)**	**0.004**
6-10 members (ref)	411 (56.8)	370 (64.1)	1		1		1		1	
>=11 members	127 (17.5)	92 (15.9)	1.23 (0.89, 1.70)	0.213	1.24 (0.89, 1.72)	0.205	1.19 (0.86, 1.65)	0.303	1.20 (0.86, 1.66)	0.283
Healthcare access										
No (ref)	582 (85.8)	460 (86.1)	1							
Yes	96 (14.2)	74 (13.9)	0.99 (0.69, 1.40)	0.941						
Ethnicity/Caste										
Dalit/ Muslim (ref)	227 (31.1)	181 (31.1)	1							
Janjati/other terai caste	343 (46.9)	274 (47.1)	1.08 (0.82, 1.42)	0.583						
Yadav/ Brahmin	161 (22.0)	127 (21.8)	1.12 (0.80, 1.56)	0.508						
Religion										
Non-Hindu	85 (11.6)	72 (12.4)	0.91 (0.63, 1.31)	0.603						
Hindu (ref)	646 (88.4)	510 (87.6)	1							
**PARENTAL CHARACTERISTICS**										
Maternal age										
15-24 years (ref)	265 (36.3)	235 (40.4)	1				1		1	
25-34 years	401 (54.9)	313 (53.8)	1.14 (0.89, 1.45)	0.310			1.24 (0.96, 1.61)	0.096	1.25 (0.96, 1.61)	0.092
35-45 years	64 (8.8)	34 (5.8)	1.61 (1.00, 2.61)	0.052			**1.94 (1.18, 3.21)**	**0.009**	**1.95 (1.18, 3.22)**	**0.009**
No of previous pregnancies										
One (ref)	223 (30.5)	189 (32.5)	1							
Two	193 (26.4)	169 (29.0)	0.94 (0.69, 1.27)	0.692						

**Indicators**	**Fed between 6-8 months (N=731) n (%)**	**Not fed at 6-8 months (N=582) n (%)**	**Unadjusted OR (95% CI)**	***p* value**	**Model 1 AOR (95% CI)**	***p* value**	**Model 2 AOR (95% CI)**	***p* value**	**Model 3 AOR (95% CI)**	***p* value**
Three	139 (19.0)	110 (18.9)	1.04 (0.74, 1.45)	0.826						
Four or more	176 (24.1)	114 (19.6)	1.23 (0.89, 1.71)	0.204						
Maternal education										
Never went to school (ref)	582 (79.6)	484 (83.2)	1				1		1	
Primary	56 (7.7)	40 (6.9)	1.06 (0.68, 1.68)	0.786			1.05 (0.66, 1.68)	0.839	1.04 (0.65, 1.65)	0.869
Secondary or above	93 (12.7)	58 (10.0)	1.44 (0.99, 2.09)	0.053			1.39 (0.94, 2.05)	1.101	1.41 (0.96, 2.06)	0.080
Father's education										
Never went to school (ref)	480 (65.7)	365 (62.7)	1				1			
Primary	78 (10.7)	79 (13.6)	0.75 (0.52, 1.08)	0.120			0.77 (0.53, 1.12)	0.175		
Secondary or above	173 (23.7)	138 (23.7)	1.01 (0.76, 1.34)	0.936			0.97 (0.72, 1.30)	0.837		
Antenatal visits										
None (ref)	207 (28.3)	209 (35.9)	1				1		1	
1-3 visits	366 (50.1)	263 (45.2)	**1.44 (1.10, 1.89)**	**0.008**			**1.45 (1.09, 1.91)**	**0.010**	**1.45 (1.09, 1.91)**	**0.010**
4+ visits	158 (21.6)	110 (18.9)	**1.52 (1.08, 2.12)**	**0.015**			**1.58 (1.11, 2.25)**	**0.010**	**1.58 (1.11, 2.24)**	**0.011**
Place of delivery										
Home (ref)	559 (77.5)	448 (79.0)	1							
Health facility	162 (22.5)	119 (21.0)	1.12 (0.84, 1.50)	0.439						
**CHILD CHARATCTERISTICS**										
Child sex										
Male (ref)	368 (50.3)	336 (57.7)	1		1		1		1	
Female	368 (49.7)	246 (42.3)	**1.34 (1.06, 1.69)**	**0.015**	**1.30 (1.02, 1.65)**	**0.032**	**1.28 (1.01, 1.63)**	**0.041**	**1.29 (1.01, 1.64)**	**0.038**
Child age group in months at follow-up survey										
7 to 24	244 (33.4)	178 (30.6)	1.08 (0.82, 1.42)	0.592	1.08 (0.82, 1.44)	0.583	1.10 (0.83, 1.47)	0.498	1.11 (0.83, 1.47)	0.481
25 to 42	188 (25.7)	168 (28.9)	0.93 (0.69, 1.23)	0.596	0.94 (0.70, 1.27)	0.703	0.95 (0.70, 1.28)	0.724	0.95 (0.71, 1.28)	0.759
43 to 59 (ref)	299 (40.9)	236 (40.5)	1		1		1		1	

For interpretation purposes, OR >1 indicates children are more likely to be introduced to solid, semi-solid or soft foods between 6-8 months and OR<1 indicates children are less likely.
Model 1 (N=1288) included child sex, child age, trial allocation as a priori covariates plus migration of HH member, MAHFP, HH size from unadjusted analysis with a p<0.2.
Model 2 (N=1300) included maternal age, maternal education, child sex, child age, trial allocation as a priori covariates plus father's education, antenatal visits from unadjusted analysis with a p<0.2 plus, HH size from Model 1 with p<0.05.
Model 3 (N=1300) included maternal age, maternal education, child sex, child age, trial allocation as a priori covariates plus HH size, antenatal visits from Model 2 with p<0.05.
Abbreviation: OR Odds Ratio, AOR Adjusted Odds Ratio

Supplementary table 9: Multivariable logistic regression analysis of factors associated with minimum dietary diversity among children aged 7-59 months

**Indicators**	**MDD achieved (N=846) n (%)**	**MDD not achieved (N=506) n (%)**	**Unadjusted OR (95% CI)**	***p* value**	**Model 1 AOR (95% CI)**	***p* value**	**Model 2 AOR (95% CI)**	***p* value**	**Model 3 AOR (95% CI)**	***p* value**
**HOUSEHOLD CHARACTERISTICS**										
Wealth Quintile										
Lowest (ref)	132 (16.2)	111 (23.0)	1		1		1		1	
Second	149 (18.3)	111 (23.0)	1.14 (0.79, 1.63)	0.491	1.19 (0.80, 1.76)	0.389	1.10 (0.76, 1.61)	0.609	1.16 (0.80, 1.68)	0.438
Middle	187 (22.9)	105 (21.7)	**1.55 (1.09, 2.21)**	**0.016**	**1.64 (1.09, 2.47)**	**0.018**	**1.53 (1.06, 2.22)**	**0.025**	**1.60 (1.11, 2.31)**	**0.012**
Fourth	164 (20.1)	79 (16.4)	**1.76 (1.21, 2.56)**	**0.003**	**1.89 (1.21, 2.96)**	**0.006**	**1.63 (1.08, 2.44)**	**0.019**	**1.79 (1.21, 2.66)**	**0.004**
Highest	184 (22.5)	77 (15.9)	**2.06 (1.41, 3.00)**	**<0.001**	**2.43 (1.51, 3.91)**	**<0.001**	**1.93 (1.28, 2.93)**	**0.002**	**2.12 (1.41, 3.17)**	**<0.001**
Months of adequate food provisioning (MAHFP)										
For up to 7 months	94 (11.2)	75 (14.9)	**0.69 (0.49, 0.98)**	**0.038**	1.06 (0.70, 1.60)	0.798				
For 8 to 11 months	202 (24.0)	110 (21.8)	1.09 (0.83, 1.45)	0.538	1.30 (0.94, 1.80)	0.109				
For 12 months (ref)	547 (64.9)	319 (63.3)	1		1					
Migration of at least one household member										
No (ref)	429 (51.0)	225 (44.8)	1		1					
Yes	412 (49.0)	277 (55.2)	**0.79 (0.63, 0.99)**	**0.041**	0.80 (0.62, 1.03)	0.082				
Household size										
1-5 members	187 (22.3)	124 (24.8)	0.82 (0.63, 1.08)	0.168	0.86 (0.64, 1.16)	0.331				
6-10 members (ref)	517 (61.6)	289 (57.7)	1		1					
>=11 members	135 (16.1)	88 (17.6)	0.87 (0.64, 1.19)	0.373	0.86 (0.61, 1.20)	0.373				
Healthcare access										
No (ref)	664 (85.8)	413 (86.8)	1							
Yes	110 (14.2)	63 (13.2)	1.07 (0.76, 1.51)	0.679						
Ethnicity/Caste										
Dalit/ Muslim (ref)	233 (27.5)	185 (36.6)	1		1					
Janjati/other terai caste	412 (48.7)	227 (44.9)	**1.50 (1.15, 1.95)**	**0.002**	1.24 (0.93, 1.65)	0.143				
Yadav/ Brahmin	201 (23.8)	94 (18.6)	**1.78 (1.28, 2.47)**	**0.001**	1.43 (0.99, 2.06)	0.060				
Religion										
Non-Hindu	98 (11.6)	60 (11.9)	0.94 (0.66, 1.34)	0.733						
Hindu (ref)	748 (88.4)	446 (88.1)	1							
**PARENTAL CHARACTERISTICS**										
Maternal age										
15-24 years (ref)	325 (38.5)	196 (38.7)	1				1		1	
25-34 years	467 (55.3)	262 (51.8)	1.07 (0.85, 1.36)	0.558			0.91 (0.62, 1.32)	0.608	0.89 (0.68, 1.15)	0.358
35-45 years	53 (6.3)	48 (9.5)	0.66 (0.42, 1.02)	0.060			0.68 (0.37,1.27)	0.229	**0.60 (0.38, 0.97)**	**0.036**
No of previous pregnancies										
One (ref)	276 (32.6)	154 (30.5)	1				1			
Two	238 (28.1)	128 (25.3)	1.04 (0.77, 1.41)	0.777			1.10 (0.76, 1.60)	0.602		
Three	162 (19.1)	94 (18.6)	0.96 (0.69, 1.33)	0.811			1.07 (0.67, 1.69)	0.788		
Four or more	170 (20.1)	129 (25.5)	0.73 (0.54, 1.00)	0.051			0.94 (0.57, 1.53)	0.791		
Maternal education										
Never went to school (ref)	670 (79.2)	427 (84.4)	1				1		1	

Indicators	MDD achieved (N=846) n (%)	MDD not achieved (N=506) n (%)	Unadjusted OR (95% CI)	*p* value	Model 1 AOR (95% CI)	*p* value	Model 2 AOR (95% CI)	*p* value	Model 3 AOR (95% CI)	*p* value
Primary	63 (7.4)	35 (6.9)	1.14 (0.73,1.77)	0.565			0.86 (0.54, 1.37)	0.524	0.91 (0.57, 1.45)	0.688
Secondary or above	113 (13.4)	44 (8.7)	**1.64 (1.13, 2.40)**	**0.010**			1.25 (0.82, 1.89)	0.303	1.31 (0.87, 1.98)	0.199
Father's education										
Never went to school (ref)	528 (62.4)	339 (67.0)	1				1			
Primary	102 (12.1)	59 (11.7)	1.10 (0.77, 1.58)	0.595			1.10 (0.76, 1.61)	0.610		
Secondary or above	216 (25.5)	108 (21.3)	1.31 (0.99, 1.72)	0.057			1.15 (0.85, 1.56)	0.367		
Antenatal visits										
None (ref)	250 (29.6)	185 (36.6)	1				1			
1-3 visits	411 (48.6)	231 (45.7)	1.31 (1.01, 1.69)	0.041			1.21 (0.91, 1.59)	0.183		
4+ visits	185 (21.9)	90 (17.8)	1.48 (1.07, 2.04)	0.019			1.37 (0.96, 1.96)	0.086		
Place of delivery										
Home (ref)	640 (77.0)	398 (80.4)	1							
Health facility	191 (23.0)	97 (19.6)	1.20 (0.90, 1.59)	0.211						
**CHILD CHARACTERISTICS**										
Child sex										
Male (ref)	455 (53.8)	268 (53.0)	1		1		1		1	
Female	391 (46.2)	238 (47.0)	0.97 (0.78, 1.22)	0.824	0.97 (0.76, 1.23)	0.810	0.97 (0.76, 1.23)	0.810	0.97 (0.77, 1.23)	0.813
Child age group in months at follow-up survey										
7 to 24	218 (25.8)	219 (43.3)	**0.40 (0.30, 0.52)**	**<0.001**	**0.39 (0.29, 0.52)**	**<0.001**	**0.36 (0.27, 0.48)**	**<0.001**	**0.36 (0.27, 0.48)**	**<0.001**
25 to 42	236 (27.9)	126 (24.9)	0.76 (0.57, 1.01)	0.062	0.74 (0.55, 1.01)	0.058	**0.70 (0.52, 0.95)**	**0.023**	**0.70 (0.52, 0.95)**	**0.023**
43 to 59 (ref)	392 (46.3)	161 (31.8)	1		1					

For interpretation purposes, OR >1 indicates children are more likely to achieve MDD and OR<1 indicates children are less likely.
Model 1 (N= 1284) included child sex, child age, trial allocation as a priori covariates plus wealth, MAHFP, migration of HH member, HH size, ethnicity from unadjusted analysis with a p<0.2.
Model 2 (N=1298) included maternal age, maternal education, child sex, child age, trial allocation as a priori covariates plus father's education, antenatal visits from unadjusted analysis with a p<0.2. plus, wealth from Model 1 with p<0.05.
Model 3 (N=1298) included maternal age, maternal education, child sex, child age, trial allocation as a priori covariates plus wealth from Model 2 with p<0.05.
Abbreviation: MDD Minimum Dietary Diversity, OR Odds Ratio, AOR Adjusted Odds Ratio

Supplementary table 10: Multivariable logistic regression analysis of factors associated with consumption of animal foods among children aged 7-59 months

**Indicators**	**Yes -fed animal foods (N=391) n (%)**	**No - not fed animal foods (N=961) n (%)**	**Unadjusted OR (95% CI)**	***p* value**	**Model 1 AOR (95% CI)**	***p* value**	**Model 2 AOR (95% CI)**	***p* value**	**Model 3 AOR (95% CI)**	***p* value**
**HOUSEHOLD CHARACTERISTICS**										
Wealth Quintile										
Lowest (ref)	79 (20.9)	164 (17.8)	1		1					
Second	83 (22.0)	177 (19.2)	0.97 (0.65, 1.44)	0.872	1.12 (0.73, 1.72)	0.607				
Middle	73 (19.3)	219 (23.8)	**0.67 (0.45, 0.99)**	**0.046**	0.85 (0.54, 1.34)	0.489				
Fourth	66 (17.5)	177 (19.2)	0.73 (0.49, 1.11)	0.142	1.05 (0.64, 1.72)	0.839				
Highest	77 (20.4)	184 (20.0)	0.79 (0.53, 1.18)	0.248	1.33 (0.79, 2.22)	0.282				
Months of adequate food provisioning (MAHFP)										
For up to 7 months	57 (14.6)	112 (11.7)	1.40 (0.97, 2.05)	0.084	1.24 (0.79, 1.96)	0.354				
For 8 to 11 months	90 (23.0)	222 (23.2)	1.21 (0.88, 1.66)	0.236	1.18 (0.83, 1.69)	0.356				
For 12 months (ref)	244 (62.4)	622 (65.1)	1		1					
Migration of at least one household member										
No (ref)	187 (47.9)	467 (49.0)	1							
Yes	203 (52.1)	486 (51.0)	1.03 (0.80, 1.33)	0.796						
Household size										
1-5 members	103 (26.7)	208 (21.8)	1.25 (0.93, 1.69)	0.134	1.30 (0.95, 1.77)	0.108				
6-10 members (ref)	231 (59.8)	575 (60.3)	1		1					
>=11 members	52 (13.5)	171 (17.9)	0.76 (0.53, 1.10)	0.147	0.82 (0.56, 1.21)	0.322				
Healthcare access										
No (ref)	309 (86.3)	768 (86.1)	1							
Yes	49 (13.7)	124 (13.9)	0.98 (0.67, 1.42)	0.914						
Ethnicity/Caste										
Dalit/ Muslim (ref)	158 (40.4)	260 (27.1)	1		1		1		1	
Janjati/other terai caste	166 (42.5)	473 (49.2)	**0.58 (0.44, 0.77)**	**<0.001**	**0.69 (0.47, 0.99)**	**0.045**	**0.68 (0.48, 0.96)**	**0.027**	**0.58 (0.43, 0.78)**	**<0.001**
Yadav/ Brahmin	67 (17.1)	228 (23.7)	**0.48 (0.34, 0.69)**	**<0.001**	**0.59 (0.37, 0.93)**	**0.023**	**0.57 (0.38, 0.87)**	**0.009**	**0.49 (0.33, 0.71)**	**<0.001**
Religion										
Non-Hindu	72 (18.4)	86 (8.9)	**2.16 (1.50, 3.13)**	**<0.001**	**1.78 (1.10, 2.86)**	**0.018**	1.54 (0.99, 2.40)	0.056		
Hindu (ref)	319 (81.6)	875 (91.9)	1		1		1			
**PARENTAL CHARACTERISTICS**										
Maternal age										
15-24 years (ref)	156 (40.0)	365 (38.0)	1				1		1	
25-34 years	213 (54.6)	516 (53.7)	0.98 (0.75, 1.26)	0.852			0.92 (0.62, 1.37)	0.691	0.89 (0.68, 1.71)	0.392
35-45 years	21 (5.4)	80 (8.3)	0.63 (0.37, 1.08)	0.093			0.64 (0.32, 1.28)	0.206	**0.52 (0.30, 0.90)**	**0.020**
No of previous pregnancies										
One (ref)	133 (34.0)	297 (30.9)	1				1			
Two	105 (26.9)	261 (27.2)	0.93 (0.67, 1.28)	0.649			0.95 (0.65, 1.41)	0.810		
Three	80 (20.5)	176 (18.3)	1.05 (0.74, 1.49)	0792			1.09 (0.68, 1.75)	0.728		
Four or more	73 (18.7)	226 (23.5)	0.72 (0.51, 1.02)	0.066			0.80 (0.48, 1.34)	0.403		

Indicators	Yes -fed animal foods (N=391) n (%)	No - not fed animal foods (N=961) n (%)	Unadjusted OR (95% CI)	*p* value	Model 1 AOR (95% CI)	*p* value	Model 2 AOR (95% CI)	*p* value	Model 3 AOR (95% CI)	*p* value
Maternal education										
Never went to school (ref)	321 (82.1)	776 (80.7)	1				1		1	
Primary	31 (7.9)	67 (7.0)	1.04 (0.65, 1.67)	0866			1.18 (0.72, 1.93)	0.521	1.19 (0.72, 1.95)	0.497
Secondary or above	39 (10.0)	118 (12.3)	0.81 (0.54, 1.21)	0.299			0.98 (0.64, 1.51)	0.937	0.98 (0.64, 1.50)	0.918
Father's education										
Never went to school (ref)	280 (71.6)	587 (61.1)	1				1		1	
Primary	38 (9.7)	123 (12.8)	0.69 (0.45, 1.03)	0.071			**0.65 (0.43, 0.99)**	**0.047**	**0.66 (0.43, 1.00)**	**0.050**
Secondary or above	73 (18.7)	251 (26.1)	**0.60 (0.44, 0.83)**	**0.002**			**0.64 (0.46, 0.89)**	**0.008**	**0.63 (0.45, 0.87)**	**0.006**
Antenatal visits										
None (ref)	118 (30.2)	317 (33.0)	1							
1-3 visits	200 (51.2)	442 (46.0)	1.19 (0.89, 1.58)	0.242						
4+ visits	73 (18.7)	202 (21.0)	0.93 (0.65, 1.33)	0.678						
Place of delivery										
Home (ref)	289 (75.7)	749 (79.3)	1							
Health facility	93 (24.3)	195 (20.7)	1.18 (0.87, 1.60)	0.289						
**CHILD CHARACTERISTICS**										
Child sex										
Male (ref)	204 (52.2)	519 (54.0)	1		1		1		1	
Female	187 (47.8)	442 (46.0)	1.07 (0.83, 1.37)	0.604	1.11 (0.85, 1.44)	0.450	1.10 (0.85, 1.43)	0.454	1.11 (0.86, 1.43)	0.426
Child age group in months at follow-up survey										
7 to 24	99 (25.3)	338 (35.2)	**0.58 (0.42, 0.78)**	**<0.001**	**0.61 (0.44, 0.84)**	**0.002**	**0.55 (0.40, 0.76)**	**<0.001**	**0.55 (0.40, 0.75)**	**<0.001**
25 to 42	115 (29.4)	247 (25.7)	0.97 (0.72, 1.31)	0.860	1.03 (0.75, 1.41)	0.862	0.98 (0.72, 1.34)	0.923	0.98 (0.72, 1.33)	0.879
43 to 59 (ref)	177 (45.3)	376 (39.1)	1		1		1		1	

For interpretation purposes, OR >1 indicates children are more likely to eat animal-source foods and OR<1 indicates children are less likely.
Model 1 (N= 1284) included child sex, child age, trial allocation as a priori covariates plus wealth, MAHFP, HH size, ethnicity, religion from unadjusted analysis with a p<0.2.
Model 2 (N=1350) included maternal age, maternal education, child sex, child age, trial allocation as a priori covariates plus parity, fathers' education from unadjusted analysis with a p<0.2 and ethnicity and religion from Model 1 with p<0.05.
Model 3 (N=1351) included maternal age, maternal education, child sex, child age, trial allocation as a priori covariates plus, fathers' education, ethnicity from Model 2 with p<0.05
Abbreviation: OR Odds Ratio, AOR Adjusted Odds Ratio

Supplementary table 11: Multivariable logistic regression analysis of factors associated with consumption of fruits and vegetables among children aged 7-59 months

Indicators	Yes - fed fruits and veg (N=1138) n (%)	No - not fed fruits and veg (N=214) n (%)	Unadjusted OR (95% CI)	*p* value	Model 1 AOR (95% CI)	*p* value	Model 2 AOR (95% CI)	*p* value	Model 3 AOR (95% CI)	*p* value
**HOUSEHOLD CHARACTERISTICS**										
Wealth Quintile										
Lowest (ref)	205 (18.7)	38 (18.7)	1							
Second	215 (19.6)	45 (22.2)	0.89 (0.55, 1.43)	0.629						
Middle	249 (22.7)	43 (21.2)	1.08 (0.67, 1.75)	0.736						
Fourth	207 (18.9)	36 (17.7)	1.09 (0.65, 1.78)	0.768						
Highest	220 (20.1)	41 (20.2)	1.00 (0.62, 1.63)	0.994						
Months of adequate food provisioning (MAHFP)										
For up to 7 months	141 (12.4)	28 (13.1)	0.95 (0.61, 1.50)	0.839						
For 8 to 11 months	267 (23.5)	45 (21.1)	1.15 (0.80, 1.67)	0.461						
For 12 months (ref)	726 (64.0)	140 (65.7)	1							
Migration of at least one household member										
No (ref)	562 (49.6)	92 (43.6)	1		1					
Yes	570 (50.4)	119 (56.4)	0.78 (0.58, 1.05)	0.106	0.80 (0.58, 1.10)	0.169				
Household size										
1-5 members	255 (22.6)	56 (26.4)	0.74 (0.52, 1.05)	0.101	0.74 (0.51, 1.08)	0.114				
6-10 members (ref)	693 (61.4)	113 (53.3)	1		1					
>=11 members	180 (16.0)	43 (20.3)	**0.68 (0.46, 1.01)**	**0.057**	0.79 (0.52, 1.20)	0.267				
Healthcare access										
No (ref)	901 (85.8)	176 (88.0)	1							
Yes	149 (14.2)	24 (12.0)	1.21 (0.76, 1.92)	0.427						
Ethnicity/Caste										
Dalit/ Muslim (ref)	344 (30.2)	74 (34.6)	1							
Janjati/other terai caste	542 (47.6)	97 (45.3)	1.24 (0.88, 1.74)	0.217						
Yadav/ Brahmin	252 (22.1)	43 (20.1)	1.31 (0.86, 2.00)	0.206						
Religion										
Non-Hindu	130 (11.4)	28 (13.1)	0.82 (0.52, 1.29)	0.398						
Hindu (ref)	1008 (88.6)	186 (86.9)	1							
**PARENTAL CHARACTERISTICS**										
Maternal age										
15-24 years (ref)	422 (37.1)	99 (46.3)	1				1		1	
25-34 years	633 (55.7)	96 (44.9)	**1.56 (1.14, 2.13)**	**0.005**			1.05 (0.65, 1.72)	0.830	1.21 (0.87, 1.68)	0.257
35-45 years	82 (7.2)	19 (8.9)	1.99 (0.57, 1.72)	0.964			0.71 (0.32, 1.57)	0.393	0.76 (0.42, 1.38)	0.368
No of previous pregnancies										
One (ref)	354 (31.1)	76 (35.7)	1				1			
Two	312 (27.4)	54 (25.4)	1.25 (0.85, 1.84)	0.249			1.16 (0.73, 1.86)	0.522		
Three	222 (19.5)	34 (16.0)	1.40 (0.90, 2.18)	0.133			1.31 (0.71, 2.42)	0.383		
Four or more	250 (22.0)	49 (23.0)	1.09 (0.73, 1.62)	0.670			1.10 (0.58, 2.08)	0.767		

Indicators	Yes - fed fruits and veg (N=1138) n (%)	No - not fed fruits and veg (N=214) n (%)	Unadjusted OR (95% CI)	*p* value	Model 1 AOR (95% CI)	*p* value	Model 2 AOR (95% CI)	*p* value	Model 3 AOR (95% CI)	*p* value
Maternal education										
Never went to school (ref)	920 (80.8)	177 (82.7)	1				1		1	
Primary	82 (7.2)	16 (7.5)	0.98 (0.56, 1.73)	0.957			0.80 (0.43, 1.46)	0.464	0.95 (0.52, 1.73)	0.870
Secondary or above	136 (12.0)	21 (9.8)	1.24 (0.76, 2.03)	0.388			1.16 (0.67, 1.99)	0.600	1.23 (0.73, 2.05)	0.437
Father's education										
Never went to school (ref)	722 (63.4)	145 (67.8)	1				1			
Primary	137 (12.0)	24 (11.2)	1.14 (0.71, 1.83)	0.597			1.04 (0.63, 1.73)	0.871		
Secondary or above	279 (24.5)	45 (21.0)	1.26 (0.88, 1.82)	0.212			1.47 (0.96, 2.24)	0.075		
Antenatal visits										
None (ref)	364 (32.0)	71 (33.2)	1							
1-3 visits	550 (48.3)	92 (43.0)	1.18 (0.84, 1.66)	0.351						
4+ visits	224 (19.7)	51 (23.8)	0.85 (0.57, 1.28)	0.441						
Place of delivery										
Home (ref)	877 (78.6)	161 (76.7)	1							
Health facility	239 (21.4)	49 (23.3)	0.89 (0.62, 1.27)	0.515						
**CHILD CHARACTERISTICS**										
Child sex										
Male (ref)	604 (53.1)	119 (55.6)	1		1		1		1	
Female	534 (46.9)	95 (44.4)	1.13 (0.83, 1.52)	0.439	1.23 (0.90, 1.68)	0.203	1.25 (0.91, 1.72)	0.160	1.23 (0.90, 1.69)	0.188
Child age group in months at follow-up survey										
7 to 24	307 (27.0)	130 (60.7)	**0.20 (0.13, 0.28)**	**<0.001**	**0.20 (0.13, 0.29)**	**<0.001**	**0.19 (0.13, 0.28)**	**<0.001**	**0.20 (0.13, 0.29)**	**<0.001**
25 to 42	321 (28.2)	41 (19.2)	0.66 (0.42, 1.04)	0.073	0.65 (0.41, 1.03)	0.068	0.65 (0.41, 1.03)	0.069	0.67 (0.43, 1.06)	0.090
43 to 59 (ref)	510 (44.8)	43 (20.1)	1		1		1		1	

For interpretation purposes, a OR >1 indicates children are more likely to eat fruits/ vegetables and OR<1 indicates children are less likely.
Model 1 (N= 1331) included child sex, child age, trial allocation as a priori covariates plus migration, HH size from unadjusted analysis with a p<0.2.
Model 2 (N=1350) included maternal age, maternal education, child sex, child age, trial allocation as a priori covariates plus parity from unadjusted analysis with a p<0.2.
Model 3 (N=1351) included maternal age, maternal education, child sex, child age, trial allocation as covariates.
Abbreviation: OR Odds Ratio, AOR Adjusted Odds Ratio
