# Supplementary material for: Feeding, caregiving practices, and developmental delay among children under five in lowland Nepal: a community-based cross-sectional survey
Source: BMC Public Health. 2022 Sep 10;22:1721. doi: 10.1186/s12889-022-13776-8 (PMC9464411; doi:10.1186/s12889-022-13776-8)
Supplement: Supplementary file 6 — Additional file 6: Supplementary Table 12. Multivariable logistic regression analysis of factors associated with access to three or more books among children aged 7–59. Supplementary Table 13. Multivariable logistic regression analysis of factors associated with access to two or more toys among children aged 7–59. Supplementary Table 14. Multivariable logistic regression analysis of factors associated with early stimulation and responsive caregiving by mother/father/other adults to children aged 24–59 months. Supplementary Table 15. Multivariable logistic regression analysis of factors associated with adequate supervision of children aged 7–59 months. Supplementary Table 16. Multivariable logistic regression analysis of factors associated with attendance of early childhood education by children aged 36–59 months. Supplementary Table 17. Multivariable logistic regression analysis of factors associated with developmentally on track according to ECDI score of children aged 36–59 months. [file 12889_2022_13776_MOESM6_ESM.pdf]

Supplementary table 12: Multivariable logistic regression analysis of factors associated with access to three or more books among children aged 7-59

Indicators	Have access (N=93) n (%)	Do not have access (N=1259) n (%)	Unadjusted OR (95% CI)	*p* value	Model 1 AOR (95% CI)	*p* value	Model 2 AOR (95% CI)	*p* value	Model 3 AOR (95% CI)	*p* value
**HOUSEHOLD CHARACTERISTICS**										
Wealth Quintile										
Lowest (ref)	17 (7.0)	226 (93.0)	1		1		1		1	
Second	11 (4.2)	249 (95.8)	0.66 (0.25, 1.73)	0.394	0.86 (0.29, 2.53)	0.780	0.52 (0.18, 1.51)	0.230	0.57 (0.20, 1.58)	0.277
Middle	13 (4.5)	279 (95.5)	0.63 (0.25, 1.55)	0.310	0.78 (0.26, 2.30)	0.654	0.45 (0.16, 1.28)	0.134	0.47 (0.17, 1.29)	0.145
Fourth	15 (6.2)	228 (93.8)	1.08 (0.44, 2.66)	0.874	1.24 (0.38, 4.01)	0.723	0.77 (0.26, 2.23)	0.626	0.84 (0.30, 2.31)	0.732
Highest	34 (13.0)	227 (87.0)	2.13 (0.94, 4.80)	0.068	2.66 (0.87, 8.17)	0.087	1.55 (0.59, 4.06)	0.376	1.69 (0.68, 4.22)	0.258
Months of adequate food provisioning (MAHFP)										
For up to 7 months	13 (7.7)	156 (92.3)	1.13 (0.49, 2.63)	0.775	2.51 (0.84, 7.48)	0.099				
For 8 to 11 months	13 (4.2)	299 (95.8)	**0.31 (0.14, 0.68)**	**0.003**	0.42 (0.16, 1.08)	0.073				
For 12 months (ref)	66 (7.7)	799 (92.3)	1		1					
Migration of at least one household member										
No (ref)	53 (8.1)	601 (91.9)	1		1					
Yes	40 (5.8)	649 (94.2)	0.58 (0.33, 1.01)	0.055	0.60 (0.32, 1.11)	0.103				
Household size										
1-5 members	25 (8.0)	286 (92.0)	1.02 (0.55, 1.90)	0.957						
6-10 members (ref)	57 (7.1)	749 (92.9)	1							
>=11 members	10 (4.5)	213 (95.5)	0.63 (0.27, 1.47)	0.284						
Health care access										
No (ref)	75 (7.0)	1002 (93.0)	1							
Yes	14 (8.1)	159 (91.9)	0.89 (0.41, 1.92)	0.759						
Ethnicity/Caste										
Dalit/ Muslim (ref)	21 (5.0)	397 (95.0)	1		1					
Janjati/other terai caste	39 (6.1)	600 (93.9)	1.51 (0.77, 2.99)	0.233	1.31 (0.59, 2.87)	0.507				
Yadav/ Brahmin	33 (11.2)	262 (88.8)	**2.20 (1.05, 4.61)**	**0.037**	1.87 (0.75, 4.66)	0.182				
Religion										
Non-Hindu	12 (7.6)	146 (92.4)	1.24 (0.55, 2.78)	0.602						
Hindu (ref)	81 (6.8)	1113 (93.2)	1							
**PARENTAL CHARACTERISTICS**										
Maternal age										
15-24 years (ref)	37 (7.1)	484 (92.9)	1				1			
25-34 years	53 (7.3)	676 (92.7)	1.04 (0.60, 1.79)	0.902			1.40 (0.71, 2.73)	0.328		
35-45 years	3 (3.0)	98 (97.0)	0.39 (0.10, 1.56)	0.182			0.63 (0.11, 3.62)	0.601		
No of previous pregnancies										
One (ref)	33 (7.7)	397 (92.3)	1							
Two	25 (6.8)	341 (93.2)	0.86 (0.43, 1.71)	0.670						
Three	21 (8.2)	235 (91.8)	1.09 (0.52, 2.26)	0.825						
Four or more	14 (4.7)	285 (95.3)	0.62 (0.28, 1.35)	0.228						

Indicators	Have access (N=93) n (%)	Do not have access (N=1259) n (%)	Unadjusted OR (95% CI)	*p* value	Model 1 AOR (95% CI)	*p* value	Model 2 AOR (95% CI)	*p* value	Model 3 AOR (95% CI)	*p* value
Maternal education										
Never went to school (ref)	66 (6.0)	1031 (94.0)	1				1		1	
Primary	8 (8.2)	90 (91.8)	1.36 (0.51, 3.59)	0.535			1.08 (0.37, 3.17)	0.895	1.04 (0.35, 3.04)	0.947
Secondary or above	19 (12.1)	138 (87.9)	**2.61 (1.26, 5.42)**	**0.010**			**2.86 (1.22, 6.70)**	**0.015**	**2.55 (1.11, 5.85)**	**0.027**
Father's education										
Never went to school (ref)	59 (6.8)	808 (93.2)	1							
Primary	11 (6.8)	150 (93.2)	0.83 (0.36, 1.96)	0.677						
Secondary or above	23 (7.1)	301 (92.9)	0.75 (0.40, 1.41)	0.376						
Antenatal visits										
None (ref)	20 (4.6)	415 (95.4)	1				1		1	
1-3 visits	40 (6.2)	602 (93.8)	1.61 (0.83, 3.16)	0.162			1.47 (0.67, 3.20)	0.332	1.50 (0.70, 3.19)	0.297
4+ visits	33 (12.0)	242 (88.0)	**2.89 (1.39, 6.00)**	**0.005**			**3.21 (1.33, 7.76)**	**0.010**	**3.05 (1.32, 7.07)**	**0.009**
Place of delivery										
Home (ref)	55 (5.3)	983 (94.7)	1				1			
Health facility	35 (12.2)	253 (87.8)	1.63 (0.91, 2.91)	0.102			1.08 (0.53, 2.23)	0.828		
**CHILD CHARACTERISTICS**										
Child sex										
Male (ref)	40 (5.5)	683 (94.5)	1		1		1		1	
Female	53 (8.4)	576 (91.6)	1.74 (1.02, 2.97)	0.043	1.93 (1.06, 3.52)	0.032	1.74 (0.94, 3.20)	0.076	1.81 (1.00, 3.29)	0.052
Child age group in months at follow-up survey										
7 to 24	37 (8.5)	400 (91.5)	1.15 (0.63, 2.08)	0.655	0.84 (0.43, 1.64)	0.603	1.10 (0.55, 2.20)	0.791	1.00 (0.52, 1.93)	0.999
25 to 42	14 (3.9)	348 (96.1)	**0.30 (1.38, 0.65)**	**0.002**	**0.21 (0.09, 0.50)**	**<0.001**	**0.23 (0.10, 0.54)**	**0.001**	**0.24 (0.10, 0.55)**	**0.001**
43 to 59 (ref)	42 (7.6)	511 (92.4)	1		1		1		1	

For interpretation purposes, OR >1 indicates children are more likely to have access to three or more books and OR<1 indicates children are less likely.
Model 1 (N= 1295) included wealth quintile, child sex, child age, trial allocation as a priori covariates plus MAHFP, migration of HH members, ethnicity from unadjusted analysis with a p<0.2.
Model 2 (N=1274) included wealth quintile, maternal education, child sex, child age, trial allocation as a priori covariates plus maternal age, antenatal visits, place of delivery from unadjusted analysis with a p<0.2.
Model 3 (N=1299) included wealth quintile, maternal education, child sex, child age, trial allocation as a priori covariates plus, antenatal visits from Model 2 with p<0.05.
Abbreviation: OR Odds Ratio, AOR Adjusted Odds Ratio

Supplementary table 13: Multivariable logistic regression analysis of factors associated with access to two or more toys among children aged 7-59

**Indicators**	**Have access (N=865) n (%)**	**Do not have access (N=487) n (%)**	**Unadjusted OR (95% CI)**	***p* value**	**Model 1 AOR (95% CI)**	***p* value**	**Model 2 AOR (95% CI)**	***p* value**
**HOUSEHOLD CHARACTERISTICS**								
Wealth Quintile								
Lowest (ref)	134 (55.1)	109 (44.9)	1		1		1	
Second	167 (64.2)	93 (35.8)	**1.53 (1.02, 2.30)**	**0.040**	**1.58 (1.03, 2.43)**	**0.037**	**1.53 (1.01, 2.32)**	**0.047**
Middle	194 (66.4)	98 (33.6)	**1.81 (1.22, 2.69)**	**0.003**	**1.92 (1.24, 2.97)**	**0.003**	**1.82 (1.21, 2.74)**	**0.004**
Fourth	160 (65.8)	83 (34.2)	**1.84 (1.21, 2.80)**	**0.004**	**1.98 (1.23, 3.19)**	**0.005**	**1.77 (1.14, 2.75)**	**0.010**
Highest	169 (64.8)	92 (35.2)	**1.83 (1.21, 2.76)**	**0.004**	**2.15 (1.32, 3.50)**	**0.002**	**1.77 (1.13, 2.76)**	**0.012**
Months of adequate food provisioning (MAHFP)								
For up to 7 months	98 (58.0)	71 (42.0)	**0.64 (0.43, 0.96)**	**0.032**	0.91 (0.56, 1.48)	0.710		
For 8 to 11 months	211 (67.6)	101 (32.4)	1.11 (0.80, 1.52)	0.534	1.23 (0.85, 1.76)	0.269		
For 12 months (ref)	553 (63.9)	313 (36.1)	1		1			
Migration of at least one household member								
No (ref)	417 (63.8)	237 (36.2)	1					
Yes	443 (64.3)	246 (35.7)	0.99 (0.77, 1.28)	0.947				
Household size								
1-5 members	193 (62.1)	118 (37.9)	0.89 (0.65, 1.20)	0.439				
6-10 members (ref)	516 (64.0)	290 (36.0)	1					
>=11 members	147 (65.9)	76 (34.1)	1.04 (0.73, 1.48)	0.818				
Health care access								
No (ref)	675 (62.7)	402 (37.3)	1					
Yes	116 (67.1)	57 (32.9)	1.18 (0.80, 1.74)	0.396				
Ethnicity/Caste								
Dalit/ Muslim (ref)	265 (63.4)	153 (36.6)	1					
Janjati/other terai caste	414 (64.8)	225 (35.2)	0.99 (0.74, 1.34)	0.972				
Yadav/ Brahmin	186 (63.1)	109 (36.9)	1.19 (0.83, 1.72)	0.338				
Religion								
Non-Hindu	102 (64.6)	56 (35.4)	1.08 (0.72, 1.62)	0.695				
Hindu (ref)	763 (63.9)	431 (36.1)	1					
**PARENTAL CHARACTERISTICS**								
Maternal age								
15-24 years (ref)	325 (62.4)	196 (37.6)	1					
25-34 years	475 (65.2)	254 (34.8)	1.07 (0.82, 1.39)	0.614				
35-45 years	64 (63.4)	37 (36.6)	0.88 (0.53, 1.45)	0.609				
No of previous pregnancies								
One (ref)	267 (62.1)	163 (37.9)	1					
Two	243 (66.4)	123 (33.6)	1.15 (0.83, 1.60)	0.405				
Three	157 (61.3)	99 (38.7)	0.85 (0.59, 1.21)	0.362				
Four or more	197 (65.9)	102 (34.1)	1.04 (0.73, 1.47)	0.833				

Indicators	Have access (N=865) n (%)	Do not have access (N=487) n (%)	Unadjusted OR (95% CI)	*p* value	Model 1 AOR (95% CI)	*p* value	Model 2 AOR (95% CI)	*p* value
Maternal education								
Never went to school (ref)	688 (62.7)	409 (37.3)	1				1	
Primary	67 (68.4)	31 (31.6)	1.21 (0.73, 1.99)	0.460			1.24 (0.74, 2.10)	0.414
Secondary or above	110 (70.1)	47 (29.9)	**1.57 (1.04, 2.35)**	**0.030**			1.37 (0.88, 2.13)	0.158
Father's education								
Never went to school (ref)	548 (63.2)	319 (36.8)	1					
Primary	101 (62.7)	60 (37.3)	1.01 (0.68, 1.50)	0.963				
Secondary or above	216 (66.7)	108 (33.3)	1.21 (0.90, 1.65)	0.210				
Antenatal visits								
None (ref)	266 (61.1)	169 (38.9)	1				1	
1-3 visits	422 (65.7)	220 (34.3)	**1.43 (1.06, 1.91)**	**0.018**			**1.38 (1.02, 1.88)**	**0.040**
4+ visits	177 (64.4)	98 (35.6)	1.21 (0.85, 1.74)	0.291			1.17 (0.79, 1.72)	0.427
Place of delivery								
Home (ref)	672 (64.7)	366 (35.3)	1					
Health facility	177 (61.5)	111 (38.5)	0.86 (0.63, 1.17)	0.345				
**CHILD CHARACTERISTICS**								
Child sex								
Male (ref)	474 (65.6)	249 (34.4)	1		1		1	
Female	391 (62.2)	238 (37.8)	**0.73 (0.57, 0.94)**	**0.016**	**0.73 (0.56, 0.95)**	**0.019**	**0.72 (0.55, 0.94)**	**0.014**
Child age group in months at follow-up survey								
7 to 24	245 (56.1)	192 (43.9)	**0.50 (0.37, 0.67)**	**<0.001**	**0.48 (0.35, 0.66)**	**<0.001**	**0.49 (0.36, 0.66)**	**<0.001**
25 to 42	239 (66.0)	123 (34.0)	0.87 (0.64, 1.20)	0.408	0.86 (0.62, 1.19)	0.365	0.87 (0.62, 1.20)	0.392
43 to 59 (ref)	381 (68.9)	172 (31.1)	1		1		1	

For interpretation purposes, OR >1 indicates children are more likely to have access to two or more toys and OR<1 indicates children are less likely.
Model 1 (N= 1295) included wealth quintile, child sex, child age, trial allocation as a priori covariates plus MAHFP covariates from unadjusted analysis with a p<0.2.
Model 2 (N=1299) included wealth quintile, maternal education, child sex, child age, trial allocation as a priori covariates plus antenatal visits from unadjusted analysis with a p<0.2.
Abbreviation: OR Odds Ratio, AOR Adjusted Odds Ratio

Supplementary table 14: Multivariable logistic regression analysis of factors associated with early stimulation and responsive caregiving by mother/father/other adults to children aged 24-59 months

Indicators	Yes- receive stimulation (N=136) n (%)	No- did not receive (N=804) n (%)	Unadjusted OR (95% CI)	*p* value	Model 1 AOR (95% CI)	*p* value	Model 2 AOR (95% CI)	*p* value
**HOUSEHOLD CHARACTERISTICS**								
Wealth Quintile								
Lowest (ref)	19 (10.6)	160 (89.4)	1		1		1	
Second	30 (16.5)	152 (83.5)	1.49 (0.73, 3.04)	0.270	1.66 (0.73, 3.74)	0.225	1.77 (0.78, 3.98)	0.169
Middle	25 (12.4)	177 (87.6)	1.02 (0.50, 2.11)	0.950	1.34 (0.57, 3.16)	0.501	1.24 (0.53, 2.90)	0.613
Fourth	19 (11.2)	150 (88.8)	1.11 (0.52, 2.39)	0.782	1.53 (0.62, 3.82)	0.358	1.50 (0.61, 3.67)	0.375
Highest	34 (20.4)	133 (79.6)	**2.20 (1.09, 4.43)**	**0.028**	2.35 (0.96, 5.77)	0.062	2.13 (0.87, 5.22)	0.100
Months of adequate food provisioning (MAHFP)								
For up to 7 months	12 (9.9)	109 (90.1)	0.66 (0.32, 1.39)	0.273				
For 8 to 11 months	39 (16.6)	196 (83.4)	1.12 (0.68, 1.84)	0.656				
For 12 months (ref)	84 (14.5)	496 (85.5)	1					
Migration of at least one household member								
No (ref)	79 (16.7)	393 (83.3)	1		1		1	
Yes	57 (12.3)	407 (87.7)	**0.47 (0.30, 0.74)**	**0.001**	**0.55 (0.33, 0.94)**	**0.028**	**0.52 (0.31, 0.90)**	**0.019**
Household size								
1-5 members	36 (16.7)	179 (83.3)	1.17 (0.71, 1.91)	0.539	1.44 (0.81, 2.58)	0.214	1.45 (0.81, 2.61)	0.214
6-10 members (ref)	81 (14.0)	496 (86.0)	1		1		1	
>=11 members	16 (11.7)	121 (88.3)	0.58 (0.30, 1.12)	0.107	**0.42 (0.18, 0.95)**	**0.037**	**0.41 (0.18, 0.94)**	**0.036**
Health care access								
No (ref)	96 (13.1)	637 (86.9)	1		1		1	
Yes	25 (21.9)	89 (78.1)	**1.89 (1.04, 3.43)**	**0.035**	**2.10 (1.04, 4.22)**	**0.038**	**2.04 (1.01, 4.13)**	**0.048**
Ethnicity/Caste								
Dalit/ Muslim (ref)	33 (11.6)	251 (88.4)	1		1			
Janjati/other terai caste	73 (16.1)	380 (83.9)	**1.75 (1.05, 2.92)**	**0.033**	1.49 (0.72, 3.09)	0.281		
Yadav/ Brahmin	30 (14.8)	173 (85.2)	1.83 (0.99, 3.41)	0.056	1.61 (0.66, 3.90)	0.294		
Religion								
Non-Hindu	12 (10.7)	100 (89.3)	0.57 (0.28, 1.17)	0.126	1.24 (0.45, 3.39)	0.678		
Hindu (ref)	124 (15.0)	704 (85.0)	1		1			
**PARENTAL CHARACTERISTICS**								
Maternal age								
15-24 years (ref)	37 (11.5)	286 (88.5)	1				1	
25-34 years	90 (16.5)	454 (83.5)	1.52 (0.95, 2.41)	0.080			**1.82 (1.03, 3.21)**	**0.040**
35-45 years	9 (12.5)	63 (87.5)	1.02 (0.42, 2.48)	0.965			1.42 (0.49, 4.12)	0.521
No of previous pregnancies								
One (ref)	38 (13.0)	254 (87.0)	1					
Two	39 (14.9)	222 (85.1)	0.98 (0.56, 1.72)	0.956				
Three	27 (15.3)	150 (84.7)	1.04 (0.57, 1.91)	0.892				
Four or more	32 (15.3)	177 (84.7)	0.99 (0.55, 1.78)	0.986				

Indicators	Yes-receive stimulation (N=136) n (%)	No- did not receive (N=804) n (%)	Unadjusted OR (95% CI)	*p* value	Model 1 AOR (95% CI)	*p* value	Model 2 AOR (95% CI)	*p* value
Maternal education								
Never went to school (ref)	97 (12.8)	660 (87.2)	1				1	
Primary	18 (26.1)	51 (73.9)	**2.35 (1.17, 4.71)**	**0.016**			**3.12 (1.34, 7.27)**	**0.008**
Secondary or above	21 (18.4)	93 (81.6)	**2.13 (1.15, 3.96)**	**0.017**			1.78 (0.81, 3.91)	0.148
Father's education								
Never went to school (ref)	85 (14.2)	512 (85.8)	1					
Primary	18 (15.8)	96 (84.2)	1.41 (0.73, 2.72)	0.311				
Secondary or above	33 (14.4)	196 (85.6)	1.02 (0.62, 1.69)	0.940				
Antenatal visits								
None (ref)	43 (13.8)	268 (86.2)	1					
1-3 visits	68 (15.1)	382 (84.9)	1.21 (0.74, 1.96)	0.448				
4+ visits	25 (14.0)	154 (86.0)	1.10 (0.59, 2.04)	0.757				
Place of delivery								
Home (ref)	108 (14.4)	643 (85.6)	1					
Health facility	24 (14.1)	146 (85.9)	1.11 (0.64, 1.93)	0.716				
**CHILD CHARACTERISTICS**								
Child sex								
Male (ref)	71 (13.8)	444 (86.2)	1		1		1	
Female	65 (15.3)	360 (84.7)	0.93 (0.61, 1.42)	0.731	1.07 (0.64,1.78)	0.809	1.05 (0.63, 1.76)	0.856
Child age group in months at follow-up survey								
24 to 35 (ref)	21 (9.6)	198 (90.4)	1		1		1	
36 to 47	31 (10.3)	269 (89.7)	1.18 (0.62, 2.26)	0.614	1.41 (0.68, 2.92)	0.351	1.23 (0.58, 2.57)	0.590
49 to 59	84 (20.0)	337 (80.0)	**2.81 (1.58, 5.00)**	**<0.001**	**3.30 (1.68, 6.47)**	**0.001**	**2.76 (1.40, 5.45)**	**0.004**

For interpretation purposes, OR >1 indicates children are more likely to have received early stimulation and responsive caregiving by mother/father/other adults and OR<1 indicates children are less likely.

Model 1 (N= 809) included wealth quintile, child sex, child age, trial allocation as a priori covariates plus migration of HH member, HH size, health care access, ethnicity, religion from unadjusted analysis with a p<0.2.

Model 2 (N=809) included wealth quintile, maternal education, child sex, child age, trial allocation as a priori covariates plus maternal age from unadjusted analysis with a p<0.2. plus, migration of HH member, HH size, health care access from Model 1 with p<0.05.

Abbreviation: OR Odds Ratio, AOR Adjusted Odds Ratio

Supplementary table 15: Multivariable logistic regression analysis of factors associated with adequate supervision of children aged 7-59 months

**Indicators**	**Yes-received adequate supervision (N=916) n (%)**	**No- did not receive (N=383) n (%)**	**Unadjusted OR (95% CI)**	***p* value**	**Model 1 AOR (95% CI)**	***p* value**	**Model 2 AOR (95% CI)**	***p* value**	**Model 3 AOR (95% CI)**	***p* value**
**HOUSEHOLD CHARACTERISTICS**										
Wealth Quintile										
Lowest (ref)	142 (61.7)	88 (38.3)	1		1		1		1	
Second	176 (69.0)	79 (31.0)	1.41 (0.94, 2.11)	0.092	1.30 (0.85, 1.99)	0.228	1.24 (0.81, 1.90)	0.325	1.25 (0.82, 1.91)	0.294
Middle	199 (69.8)	86 (30.2)	1.47 (1.00, 2.18)	0.052	1.27 (0.82, 1.97)	0.292	1.18 (0.78, 1.80)	0.436	1.19 (0.79, 1.79)	0.415
Fourth	169 (72.2)	65 (27.8)	**1.69 (1.11, 2.56)**	**0.014**	1.37 (0.84, 2.24)	0.204	1.12 (0.70, 1.79)	0.644	1.17 (0.75, 1.83)	0.498
Highest	189 (78.1)	53 (21.9)	**2.43 (1.58, 3.74)**	**<0.001**	**1.99 (1.17, 3.38)**	**0.011**	1.57 (0.94, 2.63)	0.082	1.46 (0.90, 2.38)	0.123
Months of adequate food provisioning (MAHFP)										
For up to 7 months	103 (65.2)	55 (34.8)	0.67 (0.45, 0.10)	0.045	1.07 (0.67, 1.71)	0.783				
For 8 to 11 months	214 (70.4)	90 (29.6)	0.88 (0.64, 1.21)	0.438	1.18 (0.82, 1.68)	0.368				
For 12 months (ref)	597 (71.8)	235 (28.2)	1		1					
Migration of at least one household member										
No (ref)	441 (70.4)	185 (29.6)	1							
Yes	468 (70.5)	196 (29.5)	0.99 (0.77, 1.27)	0.926						
Household size										
1-5 members	185 (63.1)	108 (36.9)	**1.64 (0.47, 0.87)**	**0.004**	**0.69 (0.50, 0.94)**	**0.020**	**0.57 (0.40, 0.80)**	**0.001**	**0.57 (0.40, 0.79)**	**0.001**
6-10 members (ref)	555 (71.5)	221 (28.5)	1		1		1		1	
>=11 members	168 (77.1)	50 (22.9)	1.27 (0.88, 1.85)	0.194	1.02 (0.69, 1.51)	0.924	1.01 (0.68, 1.52)	0.945	1.01 (0.68, 1.50)	0.974
Health care access										
No (ref)	724 (69.8)	313 (30.2)	1							
Yes	124 (76.5)	38 (23.5)	1.29 (0.86, 1.94)	0.217						
Ethnicity/Caste										
Dalit/ Muslim (ref)	265 (65.8)	138 (34.2)	1		1					
Janjati/other terai caste	447 (72.0)	174 (28.0)	1.46 (1.09, 1.96)	0.011	1.35 (0.98, 1.86)	0.063				
Yadav/ Brahmin	204 (74.2)	71 (25.8)	**1.61 (1.11, 2.33)**	**0.013**	1.46 (0.96, 2.21)	0.077				
Religion										
Non-Hindu	106 (70.7)	44 (29.3)	0.94 (0.63, 1.41)	0.764						
Hindu (ref)	810 (70.5)	339 (29.5)	1							
**PARENTAL CHARACTERISTICS**										
Maternal age										
15-24 years (ref)	378 (76.8)	114 (23.2)	1				1			
25-34 years	480 (67.9)	227 (32.1)	**0.57 (0.43, 0.75)**	**<0.001**			1.14 (0.73, 1.79)	0.561		
35-45 years	51 (57.6)	42 (42.4)	**0.34 (0.21, 0.55)**	**<0.001**			1.09 (0.54, 2.19)	0.807		
No of previous pregnancies										
One (ref)	314 (76.6)	96 (23.4)	1				1		1	
Two	267 (75.9)	85 (24.1)	0.89 (0.63, 1.27)	0.527			0.92 (0.59, 1.44)	0.731	1.04 (0.71, 1.51)	0.854
Three	166 (67.8)	79 (32.2)	**0.53 (0.36, 0.78)**	**0.001**			**0.52 (0.30, 0.90)**	**0.020**	**0.60 (0.40, 0.89)**	**0.011**
Four or more	169 (58.1)	122 (41.9)	**0.37 (0.26, 0.53)**	**<0.001**			**0.34 (0.19, 0.60)**	**<0.001**	**0.38 (0.26, 0.55)**	**<0.001**

Indicators	Yes-received adequate supervision (N=916) n (%)	No- did not receive (N=383) n (%)	Unadjusted OR (95% CI)	*p* value	Model 1 AOR (95% CI)	*p* value	Model 2 AOR (95% CI)	*p* value	Model 3 AOR (95% CI)	*p* value
Maternal education										
Never went to school (ref)	726 (68.6)	333 (31.4)	1				1		1	
Primary	72 (76.6)	22 (23.4)	1.68 (1.00, 2.84)	0.052			1.61 (0.91, 2.85)	0.104	1.62 (0.92, 2.84)	0.093
Secondary or above	118 (80.8)	28 (19.2)	**1.92 (1.22, 3.01)**	**0.005**			1.57 (0.94, 2.63)	0.086	**1.67 (1.02, 2.75)**	**0.043**
Father's education										
Never went to school (ref)	581 (69.8)	251 (30.2)	1				1			
Primary	105 (67.3)	51 (32.7)	0.93 (0.63, 1.37)	0.698			0.89 (0.59, 1.37)	0.608		
Secondary or above	230 (74.0)	81 (26.0)	1.23 (0.90, 1.67)	0.194			1.06 (0.74, 1.51)	0.742		
Antenatal visits										
None (ref)	272 (64.2)	152 (35.8)	1				1			
1-3 visits	450 (72.8)	168 (27.2)	**1.51 (1.13, 2.01)**	**0.005**			1.22 (0.89, 1.68)	0.214		
4+ visits	194 (75.5)	63 (24.5)	**1.70 (1.17, 2.46)**	**0.005**			1.15 (0.75, 1.77)	0.513		
Place of delivery										
Home (ref)	701 (69.5)	308 (30.5)	1				1			
Health facility	197 (74.1)	69 (25.9)	1.26 (0.91, 1.75)	0.159			0.93 (0.63, 1.36)	0.698		
**CHILD CHARACTERISTICS**										
Child sex										
Male (ref)	504 (71.9)	197 (28.1)	1		1		1		1	
Female	412 (68.9)	186 (31.1)	0.84 (0.65, 1.09)	0.185	0.81 (0.62, 1.60)	0.123	0.81 (0.61, 1.06)	0.128	0.81 (0.62, 1.07)	0.139
Child age group in months at follow-up survey										
7 to 24	311 (77.4)	91 (22.6)	**1.92 (1.41, 2.61)**	**<0.001**	**1.86 (1.34, 2.57)**	**<0.001**	**2.06 (1.45, 2.90)**	**<0.001**	**1.94 (1.39, 2.70)**	**<0.001**
25 to 42	248 (71.3)	100 (28.7)	1.34 (0.99, 1.83)	0.060	1.34 (0.97, 1.85)	0.074	1.38 (0.99, 1.92)	0.059	1.35 (0.97, 1.87)	0.073
43 to 59 (ref)	357 (65.0)	192 (35.0)	1		1		1		1	

For interpretation purposes, OR >1 indicates children are more likely to have adequate supervision and OR<1 indicates children are less likely.
Model 1 (N= 1231) included wealth quintile, child sex, child age, trial allocation as a priori covariates plus MAHP, HH size, ethnicity from unadjusted analysis with a p<0.2.
Model 2 (N=1212) included wealth quintile, maternal education, child sex, child age, trial allocation as a priori covariates plus maternal age, parity, fathers' education, antenatal visits, place of delivery from unadjusted analysis with a p<0.2. plus, HH size from Model 1 with p<0.05.
Model 3 (N=1235) included wealth quintile, maternal education, child sex, child age, trial allocation as a priori covariates plus, HH size, parity from Model 2 with p<0.05.
Abbreviation: OR Odds Ratio, AOR Adjusted Odds Ratio

Supplementary table 16: Multivariable logistic regression analysis of factors associated with attendance of early childhood education by children aged 36-59 months

**Indicators**	**Yes – access to ECE (N=190) n (%)**	**No- No access (N=527) n (%)**	**Unadjusted OR (95% CI)**	***p* value**	**Model 1 AOR (95% CI)**	***p* value**	**Model 2 AOR (95% CI)**	***p* value**	**Model 3 AOR (95% CI)**	***p* value**
**HOUSEHOLD CHARACTERISTICS**										
Wealth Quintile										
Lowest (ref)	30 (21.3)	111 (78.7)	1		1		1		1	
Second	31 (22.3)	108 (77.7)	0.99 (0.52, 1.89)	0.983	0.10 (0.47, 2.06)	0.975	0.96 (0.48, 1.93)	0.911	1.05 (0.53, 2.08)	0.888
Middle	35 (21.7)	126 (78.3)	1.02 (0.55, 1.91)	0.940	1.20 (0.57, 2.49)	0.633	0.98 (0.50, 1.94)	0.962	1.01 (0.51, 1.98)	0.981
Fourth	31 (25.2)	92 (74.8)	1.21 (0.63, 2.33)	0.569	1.50 (0.69, 3.28)	0.308	1.12 (0.53, 2.34)	0.767	1.18 (0.56, 2.45)	0.664
Highest	48 (41.7)	67 (58.3)	**2.79 (1.46, 5.34)**	**0.002**	**3.33 (1.51, 7.33)**	**0.003**	**2.80 (1.32, 5.97)**	**0.008**	**2.93 (1.39, 6.19)**	**0.005**
Months of adequate food provisioning (MAHFP)										
For up to 7 months	18 (19.6)	152 (77.6)	0.70 (0.36, 1.35)	0.287						
For 8 to 11 months	44 (22.4)	298 (70.1)	0.76 (0.48, 1.21)	0.246						
For 12 months (ref)	127 (29.9)	265 (70.1)	1							
Migration of at least one household member										
No (ref)	113 (29.9)	265 (70.1)	1		1					
Yes	77 (23.0)	258 (77.0)	**0.61 (0.41, 0.90)**	**0.013**	0.71 (0.44, 1.13)	0.144				
Household size										
1-5 members	41 (25.8)	118 (74.2)	1.05 (0.65, 1.68)	0.850	1.06 (0.60, 1.88)	0.829				
6-10 members (ref)	112 (25.3)	330 (74.7)	1		1					
>=11 members	34 (32.1)	72 (67.9)	1.45 (0.86, 2.47)	0.165	1.21 (0.65, 2.24)	0.547				
Health care access										
No (ref)	138 (24.6)	423 (75.4)	1		1					
Yes	29 (34.5)	55 (65.5)	1.66 (0.93, 2.95)	0.085	1.49 (0.77, 2.87)	0.232				
Ethnicity/Caste										
Dalit/ Muslim (ref)	54 (24.2)	169 (75.8)	1		1					
Janjati/other terai caste	89 (25.6)	258 (74.4)	1.15 (0.73, 1.81)	0.559	1.14 (0.64, 2.01)	0.663				
Yadav/ Brahmin	47 (32.0)	100 (68.0)	1.55 (0.89, 2.67)	0.118	1.47 (0.74, 2.92)	0.265				
Religion										
Non-Hindu	21 (23.1)	70 (76.9)	0.83 (0.46, 1.51)	0.548						
Hindu (ref)	169 (27.0)	457 (73.0)	1							
**PARENTAL CHARACTERISTICS**										
Maternal age										
15-24 years (ref)	55 (24.8)	167 (75.2)	1							
25-34 years	123 (28.0)	316 (72.0)	1.26 (0.82, 1.92)	0.288						
35-45 years	12 (21.8)	43 (78.2)	1.03 (0.46, 2.30)	0.947						
No of previous pregnancies										
One (ref)	58 (27.8)	151 (72.2)	1				1			
Two	57 (27.9)	147 (72.1)	1.08 (0.66, 1.77)	0.751			1.07 (0.61, 1.89)	0.813		
Three	40 (29.4)	96 (70.6)	1.22 (0.70, 2.11)	0.480			1.50 (0.80, 2.80)	0.202		
Four or more	34 (20.4)	133 (79.6)	0.69 (0.40, 1.19)	0.186			0.83 (0.44, 1.58)	0.578		

Indicators	Yes – access to ECE (N=190) n (%)	No- No access (N=527) n (%)	Unadjusted OR (95% CI)	*p* value	Model 1 AOR (95% CI)	*p* value	Model 2 AOR (95% CI)	*p* value	Model 3 AOR (95% CI)	*p* value
Maternal education										
Never went to school (ref)	147 (25.3)	433 (74.7)	1				1		1	
Primary	18 (31.6)	39 (68.4)	1.46 (0.73, 2.94)	0.289			1.39 (0.62, 3.12)	0.426	1.43 (0.65, 3.15)	0.381
Secondary or above	25 (31.2)	55 (68.8)	1.38 (0.77, 2.48)	0.282			0.95 (0.47, 1.94)	0.888	0.98 (0.49, 1.97)	0.957
Father's education										
Never went to school (ref)	114 (25.6)	332 (74.4)	1							
Primary	23 (23.0)	77 (77.0)	0.81 (0.46, 1.46)	0.490						
Secondary or above	53 (31.0)	118 (69.0)	1.27 (0.81, 1.99)	0.293						
Antenatal visits										
None (ref)	55 (21.7)	199 (78.3)	1				1			
1-3 visits	93 (27.4)	247 (72.6)	1.48 (0.96, 2.31)	0.079			1.33 (0.81, 2.19)	0.260		
4+ visits	42 (34.1)	81 (65.9)	**1.78 (1.02, 3.10)**	**0.041**			1.15 (0.58, 2.28)	0.680		
Place of delivery										
Home (ref)	138 (23.4)	451 (76.6)	1				1		1	
Health facility	46 (41.1)	66 (58.9)	**2.07 (1.26, 3.41)**	**0.004**			**1.94 (1.06, 3.57)**	**0.032**	**1.95 (1.10, 3.46)**	**0.022**
**CHILD CHARACTERISTICS**										
Child sex										
Male (ref)	113 (28.5)	283 (71.5)	1		1		1		1	
Female	77 (24.0)	244 (76.0)	0.89 (0.59, 1.32)	0.549	0.91 (0.57, 1.45)	0.679	0.74 (0.47, 1.17)	0.199	0.78 (0.49, 1.22)	0.272
Child age group in months at follow-up survey										
36 to 47 (ref)	48 (16.1)	251 (83.9)	1		1		1		1	
48 to 59	142 (34.0)	276 (66.0)	**3.53 (2.28, 5.48)**	**<0.001**	**3.91 (2.40, 6.38)**	**<0.001**	**3.85 (2.38, 6.23)**	**<0.001**	**3.87 (2.40, 6.24)**	**<0.001**

For interpretation purposes, a OR >1 indicates children are more likely to have attended early childhood education and OR<1 indicates children are less likely.
Model 1 (N= 611) included wealth quintile, child sex, child age, trial allocation as a priori covariates plus migration of HH member, health care access, HH size, ethnicity from unadjusted analysis with a p<0.2.
Model 2 (N=665) included wealth quintile, maternal education, child sex, child age, trial allocation as a priori covariates plus parity, antenatal visits, place of delivery from unadjusted analysis with a p<0.2.
Model 3 (N=665) included wealth quintile, maternal education, child sex, child age, trial allocation as a priori covariates plus place of delivery from Model 2 with p<0.05.
Abbreviation: OR Odds Ratio, AOR Adjusted Odds Ratio, ECE Early Childhood Education

Supplementary table 17: Multivariable logistic regression analysis of factors associated with developmentally on track according to ECDI score of children aged 36-59 months

Indicators	Yes- on tack (N=277) n (%)	No- not on track (N=444) n (%)	Unadjusted OR (95% CI)	*p* value	Model 1 AOR (95% CI)	*p* value	Model 2 AOR (95% CI)	*p* value	Model 3 AOR (95% CI)	*p* value
**HOUSEHOLD CHARACTERISTICS**										
Wealth Quintile										
Lowest (ref)	49 (34.3)	94 (65.7)	1		1		1		1	
Second	46 (33.1)	93 (66.9)	0.88 (0.51, 1.52)	0.636	0.95 (0.55, 1.65)	0.858	0.94 (0.54. 1.65)	0.836	0.96 (0.55, 1.67)	0.882
Middle	68 (42.0)	94 (58.0)	1.39 (0.83, 2.34)	0.211	1.51 (0.89, 2.54)	0.125	1.59 (0.93, 2.71)	0.089	1.61 (0.95, 2.73)	0.078
Fourth	46 (37.4)	77 (62.6)	0.91 (0.52, 1.60)	0.755	1.01 (0.57, 1.77)	0.983	1.10 (0.61, 1.96)	0.755	1.11 (0.62, 1.97)	0.725
Highest	48 (41.4)	68 (58.6)	1.29 (0.73, 2.27)	0.386	1.38 (0.78, 2.45)	0.274	1.57 (0.84, 2.92)	0.156	1.62 (0.88, 2.98)	0.120
Months of adequate food provisioning (MAHFP)										
For up to 7 months	36 (39.1)	56 (60.9)	1.27 (0.75, 2.16)	0.369						
For 8 to 11 months	77 (38.5)	123 (61.5)	1.02 (0.69, 1.51)	0.906						
For 12 months (ref)	163 (38.4)	262 (61.6)	1							
Migration of at least one household member										
No (ref)	139 (36.6)	241 (63.4)	1							
Yes	135 (40.1)	202 (59.9)	1.16 (0.82, 1.62)	0.400						
Household size										
1-5 members	72 (45.3)	87 (54.7)	1.29 (0.86, 1.94)	0.218						
6-10 members (ref)	165 (37.0)	281 (63.0)	1							
>=11 members	36 (34.0)	70 (66.0)	0.83 (0.51, 1.35)	0.452						
Health care access										
No (ref)	207 (36.8)	355 (63.2)	1							
Yes	36 (42.4)	49 (57.6)	1.25 (0.75, 2.09)	0.400						
Ethnicity/Caste										
Dalit/ Muslim (ref)	84 (37.3)	141 (62.7)	1							
Janjati/other terai caste	132 (37.8)	217 (62.2)	1.04 (0.70, 1.53)	0.853						
Yadav/ Brahmin	61 (41.5)	86 (58.5)	1.21 (0.75, 1.95)	0.444						
Religion										
Non-Hindu	35 (38.0)	57 (62.0)	0.87 (0.52, 1.46)	0.600						
Hindu (ref)	242 (38.5)	387 (61.5)	1							
**PARENTAL CHARACTERISTICS**										
Maternal age										
15-24 years (ref)	78 (35.1)	144 (64.9)	1				1			
25-34 years	175 (39.5)	268 (60.5)	1.29 (0.89, 1.86)	0.184			0.93 (0.53, 1.65)	0.813		
35-45 years	23 (41.8)	32 (58.2)	1.55 (0.79, 3.06)	0.202			1.04 (0.41, 2.64)	0.939		
No of previous pregnancies										
One (ref)	71 (33.8)	139 (66.2)	1				1			
Two	85 (41.7)	119 (58.3)	1.55 (1.00, 2.42)	0.050			1.61 (0.91, 2.86)	0.100		
Three	52 (37.7)	86 (62.3)	1.32 (0.80, 2.16)	0.273			1.39 (0.70, 2.75)	0.351		
Four or more	68 (40.5)	100 (59.5)	1.60 (1.00, 2.55)	0.048			1.73 (0.84, 3.54)	0.136		

Indicators	Yes- on tack (N=277) n (%)	No- not on track (N=444) n (%)	Unadjusted OR (95% CI)	*p* value	Model 1 AOR (95% CI)	*p* value	Model 2 AOR (95% CI)	*p* value	Model 3 AOR (95% CI)	*p* value
Maternal education										
Never went to school (ref)	230 (39.4)	354 (60.6)	1				1		1	
Primary	19 (33.3)	38 (66.7)	0.76 (0.40, 1.44)	0.402			0.63 (0.31, 1.26)	0.189	0.60 (0.30, 1.19)	0.141
Secondary or above	28 (35.0)	52 (65.0)	0.78 (0.46, 1.34)	0.372			0.72 (0.39, 1.32)	0.282	0.68 (0.38, 1.24)	0.207
Father's education										
Never went to school (ref)	170 (37.9)	279 (62.1)	1							
Primary	38 (37.6)	63 (62.4)	1.11 (0.68, 1.81)	0.687						
Secondary or above	69 (40.4)	102 (59.6)	1.06 (0.71, 1.58)	0.791						
Antenatal visits										
None (ref)	90 (35.3)	165 (64.7)	1				1			
1-3 visits	130 (38.1)	211 (61.9)	1.20 (0.82, 1.75)	0.347			1.18 (0.78, 1.77)	0.433		
4+ visits	57 (45.6)	68 (54.4)	1.58 (0.97, 2.58)	0.064			1.59 (0.93, 2.74)	0.091		
Place of delivery										
Home (ref)	220 (37.2)	372 (62.8)	1							
Health facility	49 (43.4)	64 (56.6)	1.20 (0.77,1.90)	0.422						
**CHILD CHARACTERISTICS**										
Child sex										
Male (ref)	140 (35.4)	256 (64.6)	1		1		1		1	
Female	137 (42.2)	188 (57.8)	1.40 (0.99, 1.97)	0.057	1.34 (0.94, 1.92)	0.107	1.38 (0.96, 1.99)	0.081	1.37 (0.95, 1.96)	0.088
Child age group in months at follow-up survey										
36 to 47 (ref)	103 (34.3)	197 (65.7)	1		1		1		1	
48 to 59	174 (41.3)	247 (58.7)	**1.45 (1.03, 2.04)**	**0.034**	1.44 (1.01, 2.05)	0.045	**1.45 (1.01, 2.09)**	**0.043**	**1.46 (1.02, 2.08)**	**0.037**

For interpretation purposes, a OR >1 indicates children are more likely to on track with their ECDI score and OR<1 indicates children are less likely.

Model 1 (N= 683) included wealth quintile, child sex, child age, trial allocation as a priori covariates. There were no HH level covariates from unadjusted analysis with a p<0.2.

Model 2 (N=682) included wealth quintile, maternal education, child sex, child age, trial allocation as a priori covariates plus maternal age, parity, antenatal visits from unadjusted analysis with a p<0.2.

Model 3 (N=683) included wealth quintile, maternal education, child sex, child age, trial allocation covariates.

Abbreviation: ECDI Early Childhood Development Index, OR Odds Ratio, AOR Adjusted Odds Ratio
