# Supplementary material for: Feeding, caregiving practices, and developmental delay among children under five in lowland Nepal: a community-based cross-sectional survey
Source: BMC Public Health. 2022 Sep 10;22:1721. doi: 10.1186/s12889-022-13776-8 (PMC9464411; doi:10.1186/s12889-022-13776-8)
Supplement: Supplementary file 7 — Additional file 7: Supplementary Table 18. Matrix summarising the multivariable analysis in the Model 3 for each indicator*. [file 12889_2022_13776_MOESM7_ESM.pdf]

Supplementary table 18: Matrix summarising the multivariable analysis in the Model 3 for each indicator*

	Infant and young child feeding practices indicators								Cognitive and socio-emotional caregiving practices indicators					
**Variables**	**Timely Initiation of breastfeeding**	**Colostrum feeding**	**No pre-lacteal feeding**	**Past 24 hours exclusive breastfeeding**	**Introduction of solid, semi-solid or soft foods**	**Minimum dietary diversity**	**Consumption of animal foods**	**Consumption of fruits and vegetables**	**Access to 3+ children’s books**	**Access to 2+ toys**	**Early stimulation and responsive caregiving**	**Adequate supervision**	**Participate in early childhood education**	**ECDI score**
**Household characteristics**														
Higher wealth quintile		↑		↑		↑			0	↑	0	0	↑	0
Higher MAHFP														
No migration											↓			
Household size**					↑						↓	↓		
Have access to health care				↓							↑			
Advantaged caste							↓							
Hindu religion														
**Parental characteristics**														
Older mothers	0	0	↓	0	↑	↓	↓	0			↑			
More than one child	↑											↓		
Higher maternal education	0	0	0	0	0	0	0	0	↑	0	↑	↑	0	0
Higher father’s education							↓							
One or more antenatal visits					↑				↑	↑				
Delivery at institution	↑	↑	↑										↑	
**Child characteristics**														
Female child	0	0	0	0	↑	0	0	0	0	↓	0	0	0	0
Older children***	↓	↓	↓	0	0	↑	↑	↑	↑	↑	↑	↓	↑	↑

* ↑ indicates positive (significant $p<0.05$) association**, 0** indicates null association**,** ↓ indicates negative (significant $p<0.05$) association, grey shading indicates overlapping factors in same direction

**** The household size reference group was 6-10 members

*** Reference group in child age variable for minimum dietary diversity, consumption of animal-source foods, consumption of vegetables/fruits, access to books, access to toys and adequate supervision were older children (43-59 months) and younger children for early stimulation and responsive caregiving (24-35 months), attendance of early childhood education and ECDI score (36-43 months). The age of children at recall for four breastfeeding indicators were in months as a continuous variable.
